# Supplementary material for: Factors associated with attendance at screening for breast cancer: a systematic review and meta-analysis
Source: BMJ Open. 2021 Nov 30;11(11):e046660. doi: 10.1136/bmjopen-2020-046660 (PMC8634222; doi:10.1136/bmjopen-2020-046660)
Supplement: Supplementary data [file bmjopen-2020-046660supp001.pdf]

NIHR | National Institute for Health Research

PROSPERO
International prospective register of systematic reviews

A systematic review to identify the worldwide predictors of breast screening uptake

Rebecca Crosby, Sian Williamson, Chris Stinton, Aileen Clarke, Sian Taylor-Phillips

Citation

Rebecca Crosby, Sian Williamson, Chris Stinton, Aileen Clarke, Sian Taylor-Phillips. A systematic review to identify the worldwide predictors of breast screening uptake. PROSPERO 2016 CRD42016051597 Available from:
https://www.crd.york.ac.uk/prospero/display_record.php?ID=CRD42016051597

Review question

What are the predictors of breast cancer screening uptake worldwide?

Searches

The electronic databases that will be searched to identify published studies are:
EMBASE (via Ovid), MEDLINE (via Ovid), CINAHL (The Cumulative Index to Nursing and Allied Health Literature), PsycINFO, Cochrane Library (Wiley) including the Cochrane Database of Systematic Reviews and Thomson Reuters Web of Science (all databases including Science Citation Index, Conference Proceedings and Science Citation Index Expanded and Social Sciences Index).
Reference lists of included papers and relevant reviews will be searched for papers that were not identified by the electronic search.
Experts in the field will be contacted to identify further significant papers.

Types of study to be included

Included:Any quantitative study type that mentions uptake rates of breast screening;Study must include at least one predictor of uptake to be included.Excluded:Case studies, editorials, letters and commentaries.

Condition or domain being studied

Breast screening. Predictors of uptake.

Participants/population

Inclusion:
Women of screening age (variable worldwide).
Exclusion:
Women with previously diagnosed breast cancer;
Women attending diagnostic screening;
Non-human studies.

Intervention(s), exposure(s)

Any intervention related to uptake of breast screening will be included in the review. Studies will be included where they mention uptake rates of breast screening - either current, previous or changes. The study must mention at least one predictor variable of uptake.

Comparator(s)/control

Not applicable.

Main outcome(s)

Worldwide predictors of uptake of breast screening.

* Measures of effect

The outcomes will be measured in terms of uptake, i.e. rate or percentages.

Additional outcome(s)

None.

* Measures of effect

Not applicable.

Data extraction (selection and coding)

A two-step process will be used to identify relevant studies at abstract and title stage and then at full text stage using pre-defined screening criteria. Two researchers will screening the titles and abstracts against inclusion and inclusion criteria independently using the results from the search. if a decision cannot be made on the title and abstract a full text review will be performed.
Where there are disagreements between the two researchers, a third reviewer will be contacted until a consensus is reached.
The full texts of the included studies will be obtained and undergo a second screen by two researchers and again any discrepancies resolved by the third reviewer.
Reasons for inclusion and exclusion will be stated where appropriate. The PRISMA flow diagram will be provided in the review.
Data extraction will take place after the full text review and will include:
General - authors, year, publication journal, study title, article type, stated aims, period of study;
Study characteristics - country, setting, screening programme style in this country;
Study design - cohort, case-control, prospective, retrospective, randomised controlled trial, etc.;
Participants - population;
Outcomes - primary and secondary outcomes definitions, validity of measures used, data collection method;
Predictors - number of predictors, type of predictors, definition of predictors;
overall results;
Overview - strengths and limitations of the study, Was the study blinded?; source(s) of research funding, potential conflicts of interest.
The domains involved in data extraction are broad and comprehensive due to the variability of the potential studies to be included within this review. A piloted data extraction form will be used by the two researchers to test. Any discrepancies will be discussed and a third reviewer will be involved where necessary to reach a consensus.

Risk of bias (quality) assessment

Extracted data will be stored in tabular format on Microsoft Access spreadsheet to complete the methodological quality assessment/risk of bias scoring.
Quality assessment of the included studies will be completed using the quality assessment tool.

Strategy for data synthesis

Descriptive analysis will be presented in tabular format to describe the included studies. Significant hetergeneity is expected to be found amongst the included papers considering the differences between screening programmes internationally. Therefore pooling data in a meta-regression would not be appropriate. Instead, a narrative synthesis will be adopted to explain and summarise results by predictor. This narrative synthesis will analyse the population characteristics, predictor variables and their effects on uptake rate.

Analysis of subgroups or subsets

None planned.

Contact details for further information

Rebecca Crosby
R.Crosby@warwick.ac.uk

Organisational affiliation of the review

University of Warwick

Review team members and their organisational affiliations

Miss Rebecca Crosby. University of Warwick
Miss Sian Williamson. University of Warwick
Dr Chris Stinton. University of Warwick
Professor Aileen Clarke. University of Warwick

Dr Sian Taylor-Phillips. University of Warwick

Type and method of review
Systematic review

Anticipated or actual start date
21 November 2016

Anticipated completion date
31 July 2017

Funding sources/sponsors
This systematic review presents independent research funded by the NIHR Collaboration for Leadership in Applied Health Research and Care West Midlands (CLAHRC-WM) initiative. The views expressed are those of the author(s) and not necessarily those of the NHS, the NIHR or the Department of Health.

Conflicts of interest
None known

Language
English

Country
England

Stage of review
Review Ongoing

Subject index terms status
Subject indexing assigned by CRD

Subject index terms
Breast Neoplasms; Developed Countries; Developing Countries; Early Detection of Cancer; Early Diagnosis; Healthcare Disparities; Humans; Mass Screening; Patient Acceptance of Health Care; Socioeconomic Factors

Date of registration in PROSPERO
17 November 2016

Date of first submission

Stage of review at time of this submission

Stage	Started	Completed
Preliminary searches	No	No
Piloting of the study selection process	No	No
Formal screening of search results against eligibility criteria	No	No
Data extraction	No	No
Risk of bias (quality) assessment	No	No
Data analysis	No	No

Revision note
Updated the prospero registration to be more accurate. Updated inclusion criteria to only include quantitative studies due to the number of results found after sifting.

The record owner confirms that the information they have supplied for this submission is accurate and complete and they understand that deliberate provision of inaccurate information or omission of data may be construed as scientific misconduct.

The record owner confirms that they will update the status of the review when it is completed and will add publication details in due course.

Versions

17 November 2016
23 June 2017

PROSPERO
This information has been provided by the named contact for this review. CRD has accepted this information in good faith and registered the review in PROSPERO. The registrant confirms that the information supplied for this submission is accurate and complete. CRD bears no responsibility or liability for the content of this registration record, any associated files or external websites.
