# Supplementary material for: Factors associated with attendance at screening for breast cancer: a systematic review and meta-analysis
Source: BMJ Open. 2021 Nov 30;11(11):e046660. doi: 10.1136/bmjopen-2020-046660 (PMC8634222; doi:10.1136/bmjopen-2020-046660)
Supplement: Supplementary data [file bmjopen-2020-046660supp002.pdf]

Supplementary file B: Search strategy

Database: Ovid MEDLINE(R) <1946 onwards>

Search Strategy:

----

1 exp Breast/ or breast*.mp.

2 (screen* or early detection or mammogra* or mass screening or screening program* or mammogra* screen* or direct to consumer or health screen*).mp. [mp=title, abstract, original title, name of substance word, subject heading word, floating sub-heading word, keyword heading word, organism supplementary concept word, protocol supplementary concept word, rare disease supplementary concept word, unique identifier, synonyms]

3 (uptake or adheren* or complian* or patient acceptance of healthcare or patient acceptance or patient access or attend*).mp. [mp=title, abstract, original title, name of substance word, subject heading word, floating sub-heading word, keyword heading word, organism supplementary concept word, protocol supplementary concept word, rare disease supplementary concept word, unique identifier, synonyms]

4 1 and 2 and 3

5 limit 4 to humans

6 limit 5 to english language

Database: Ovid MEDLINE(R) Daily Update, Ovid MEDLINE(R) Epub Ahead of Print and In-Process, In-Data-Review & Other Non-Indexed Citations

Search Strategy:

----

1 exp Breast/ or breast*.mp.

2 (screen* or early detection or mammogra* or mass screening or screening program* or mammogra* screen* or direct to consumer or health screen*).mp. [mp=title, abstract, original title, name of substance word, subject heading word, floating sub-heading word, keyword heading word, organism supplementary concept word, protocol supplementary concept word, rare disease supplementary concept word, unique identifier, synonyms]

3 (uptake or adheren* or complian* or patient acceptance of healthcare or patient acceptance or patient access or attend*).mp. [mp=title, abstract, original title, name of substance word, subject heading word, floating sub-heading word, keyword heading word, organism supplementary concept

word, protocol supplementary concept word, rare disease supplementary concept word, unique identifier, synonyms]

4 1 and 2 and 3

Database: Embase Classic+Embase <1947 onwards>

Search Strategy:

----

1 exp Breast/ or breast*.mp.

2 (screen* or early detection or mammogra* or mass screening or screening program* or mammogra* screen* or direct to consumer or health screen*).mp. [mp=title, abstract, heading word, drug trade name, original title, device manufacturer, drug manufacturer, device trade name, keyword, floating subheading word, candidate term word]

3 (uptake or adheren* or complian* or patient acceptance of healthcare or patient acceptance or patient access or attend*).mp. [mp=title, abstract, heading word, drug trade name, original title, device manufacturer, drug manufacturer, device trade name, keyword, floating subheading word, candidate term word]

4 1 and 2 and 3

5 limit 4 to humans

6 limit 5 to english language

Database: APA PsycInfo <1806 onwards>

Search Strategy:

----

1 breast.mp. or exp Breast/

2 (screen* or early detection or mammogra* or mass screening or screening program* or mammogra* screen* or direct to consumer or health screen*).mp. [mp=title, abstract, heading word, table of contents, key concepts, original title, tests & measures, mesh] (132904)

3 (uptake or adheren* or complian* or "patient acceptance of healthcare" or "patient acceptance" or attend*).mp. [mp=title, abstract, heading word, table of contents, key concepts, original title, tests & measures, mesh]

4 1 and 2 and 3

5 limit 4 to yr="2017 -Current"

6 limit 5 to human

7 limit 6 to english language

Database: Web of Science, 1900 onwards

TOPIC: (breast*) *AND* **TOPIC:** (screen* or "early detection" or mammogra* or "mass screening" or "screening program*" or "mammogra* screen*" or "direct to consumer" or "health screen") *AND* **TOPIC:** (uptake or adheren* or complian* or "patient acceptance of healthcare" or "patient acceptance" or "patient access" or attend*)

Database: CINAHL (Limiters - Publication Year: 1987-June 2019)

S1. TX breast’

S2. TX screen’ OR early detection OR TX mammogra’ Or TX mass screening OR TX screening program’ OR TX mammogra’ screen’ OR TX direct to consumer OR TX health screen’

S3. TX uptake OR TX adheren’ OR TX complian’ OR TX patient acceptance of health care OR TX patient acceptance OR TX patient access OR TX health screen’

S4. S1 AND S2 AND S3

Database: Cochrane Library

S1. TX breast’

S2. TX screen’ OR early detection OR TX mammogra’ Or TX mass screening OR TX screening program’ OR TX mammogra’ screen’ OR TX direct to consumer OR TX health screen’

S3. TX uptake OR TX adheren’ OR TX complian’ OR TX patient acceptance of health care OR TX patient acceptance OR TX patient access OR TX health screen’

S4. S1 AND S2 AND S3
