# Supplementary material for: Factors associated with attendance at screening for breast cancer: a systematic review and meta-analysis
Source: BMJ Open. 2021 Nov 30;11(11):e046660. doi: 10.1136/bmjopen-2020-046660 (PMC8634222; doi:10.1136/bmjopen-2020-046660)
Supplement: Supplementary data [file bmjopen-2020-046660supp004.pdf]

Breast cancer screening attendance factors: Systematic review

Supplementary file D: Characteristics of included studies

*indicates studies that could be included in meta-analysis or narrative analysis

Author	Publication year	Study country	N in analysis	Study start	Study end	Study design	Factors analysed	Odds ratio (95%CI)
Allgood	2016	UK	22828	unclear	unclear	RCT	NA	NA
Andersen*	2008	Denmark	22653	1999	2001	retrospective cohort	Previous result of mammogram	0.80 (0.62–1.03)
Barlow	2019	USA	305568	2012	2013	cohort	Age	NA
Berens*	2014	Germany	423649	2010	2011	cohort	Age	0.94 (0.93–0.96)
Blanchard*	2004	USA	18851	1985	2002	retrospective cohort	Non-English language spoken	0.33 (0.28–0.39)
							Member of majority racial/ethnic group	1.70 (1.52–1.89)
Bourmaud	2016	France	15844	2009	2009	RCT	NA	NA
Chan*	2014	Canada	504288	1999	2010	retrospective cohort	SES:	
							Medium vs low	1.35 (1.33–1.37)
							High vs low	1.76 (1.72–1.79)
							Medium vs high	1.30 (1.28–1.32)
							Diabetes	0.79 (0.78–0.80)
Chiarelli*	2003	Canada	125250	1990	1995	retrospective cohort	Age at initial screen	0.99 (0.96–1.02)
Chochinov*	2009	Canada	110240	2002	2004	retrospective cohort	Schizophrenia	0.58 (0.52–0.64)
DeBorde*	2018	France	4805390	2013	2014	cohort	SES:	
							Medium vs low	1.09 (1.09–1.10)
							High vs low	0.75 (0.74–0.75)
							Medium vs high	0.69 (0.68–0.69)
Douglas	2016	UK	NA	2012	2012	cohort	NA	NA

Mottram R, *et al. BMJ Open* 2022; 11:e046660. doi: 10.1136/bmjopen-2020-046660

Breast cancer screening attendance factors: Systematic review

Finney Rutten	2014	USA	62754	2004	2005	cohort	NA	NA
Gatrell	1998	UK	24000	1988	1995	cohort	NA	NA
Goldzahl	2018	France	26495	2015	2015	RCT	NA	NA
Hyndman	2000	Australia	5968	1991	1996	retrospective cohort	NA	NA
Jensen*	2012b/2015b	Denmark	144264	2008	2009	cohort	Education:	
							Medium vs low	1.45 (1.41–1.49)
							High vs low	1.31 (1.27–1.36)
							Medium vs high	0.91 (0.88–0.94)
							Housing tenure (homeowner vs non)	2.20 (2.14–2.26)
							Marital status (non-married/non-cohabiting vs married/cohabiting)	2.27 (2.21–2.33)
							Income:	
							Intermediate vs low	1.91 (1.85–1.97)
							High vs low	2.52 (2.44–2.60)
							Intermediate vs high	1.32 (1.27–1.36)
							Access to vehicle	0.33 (0.32– to 0.34)
							Employment status:	
							Outside workforce vs employed/self-employed	0.66 (0.64– to 0.68)
							Unemployed vs employed/self-employed	0.41 (0.40– to 0.43)
							Distance to screening centre	0.86 (0.84– to 0.88)

Breast cancer screening attendance factors: Systematic review

Katz*	2018	Israel	44318	2008	2014	cohort	Age	1.06 (1.00–1.12)
							Current smoker	0.72 (0.65–0.79)
							Limiting long-term illness	0.64 (0.61– to 0.66)
							60+ primary care visits during 6-year study period (vs <60)	2.70 (2.55–2.86)
							Depression	1.12 (1.02–1.23)
							Diabetes	1.25 (1.17–1.33)
							Heart disease	1.75 (1.61–1.91)
							BMI	0.95 (0.87–1.04)
Kee*	1993	Northern Ireland	600	1991	1991	cross-sectional	Negative attitude about breast screening	0.44 (0.35–0.55)
Lagerlund*	2002	Sweden	46041	1988	1997	cohort	Age	0.98 (0.90–1.06)
							Country of origin (immigrant vs non)	1.75 (1.62–1.90)
							Education:<br>Medium vs low<br>High vs low<br>Medium vs high	 1.20 (1.11–1.30)<br>1.16 (1.07–1.25)<br>0.96 (0.88–1.05)
							Housing tenure (homeowner vs non)	2.06 (1.92–2.21)

Mottram R, *et al. BMJ Open* 2022; 11:e046660. doi: 10.1136/bmjopen-2020-046660

Breast cancer screening attendance factors: Systematic review

							Income:	
							Intermediate vs low	1.96 (1.80–2.14)
							High vs low	1.61 (1.26–2.05)
							Intermediate vs high	0.82 (0.65–1.03)
							Number of childbirths:	
							0 vs 1–2	0.44 (0.40–0.48)
							3+ vs 1–2	0.81 (0.75–0.87)
Lagerlund*	2015	Sweden	46041	2005	2009	cohort	SES:	
							Medium vs low	2.35 (2.20–2.51)
							High vs low	3.59 (3.22–4.00)
							Medium vs high	1.53 (1.38–1.69)
Larsen*	2018	Denmark	91517	2008	2009	cohort	Age	0.95 (0.91–0.98)
							Country of origin (immigrant vs non)	2.24 (2.10–2.40)
							Education:	
							Medium vs low	1.44 (1.39–1.49)
							High vs low	1.26 (1.21–1.31)
							Medium vs high	0.87 (0.84–0.91)
							Income:	
							Intermediate vs low	2.09 (2.01–2.18)
							High vs low	2.87 (2.76–2.99)
							Intermediate vs high	1.37 (1.32–1.43)
							No comorbidities	1.53 (1.46–1.60)
Le*	2019	Norway	885979	1996	2015	cohort	Age	1.11 (1.09–1.12)
							Country of origin (immigrant vs non)	2.81 2.77–2.85)

Breast cancer screening attendance factors: Systematic review

							Marital status (non-married/non-cohabiting vs married/cohabiting)	1.58 (1.56–1.59)
							Income:	
							Intermediate vs low	1.78 (1.76–1.81)
							High vs low	1.69 (1.65–1.72)
							Intermediate vs high	0.95 (0.93–0.96)
							Receiving disability benefits	0.70 (0.70–0.71)
							Employment status (vs employed/self-employed):	
							Outside workforce	0.51 (0.50–0.51)
							Unemployed	0.47 (0.45–0.49)
							Not living in capital city	1.94 (1.91–1.97)
							Citizen of country	2.88 (2.82–2.94)
Leung*	2015	UK	27416	2008	20101	cohort	Residence (rural vs urban)	1.11 (1.04–1.19)
Lim*	2010	South Korea	3705246	2008	2008	cohort	Age	1.42 (1.41–1.43)
Luckman	2019	USA	10063	2010	2014	RCT	NA	NA
Makedonov*	2015	Canada	105665	2005	2011	case-control	Age at initial screen	1.05 (1.02–1.08)
							Physician years since graduation	1.03 (0.99–1.06)
Matson*	2001	Sweden	32605	1990	1994	cohort	SES:	
							Medium vs low	1.23 (1.17–1.30)
							High vs low	1.84 (1.73–1.96)
							Medium vs high	1.49 (1.41–1.59)
Maxwell*	2013	UK	253017	2005	2008	retrospective cohort	Previous result of mammogram	0.89 (0.83–0.95)

Mayer	2000	USA	1562	1995	1998	RCT	NA	NA
McCann*	2002	UK	113409	1989	1991	retrospective cohort	Previous result of mammogram	0.82 (0.76–0.89)
Meldrum	1994	UK	3083	1992	1993	RCT	NA	NA
Merrick	2015	USA	4427	2010	2012	RCT	NA	NA
Moss	2001	UK	210939	1996	unclear	retrospective cohort	NA	NA
O'Byrne*	2000	Australia	119502	1995	1996	retrospective cohort	No family history of breast cancer<br><br>Type of clinic (mobile vs fixed)<br><br>Never HRT use	0.90 (0.86–0.94)<br><br>0.93 (0.88–0.98)<br><br>1.13 (1.09–1.17)
Offman	2013	UK	12929	2010	2011	RCT	NA	NA
Oh*	2011	South Korea	2511976	2005	2008	retrospective cohort	Previous result of mammogram	0.88 (0.88–0.89)
Ore*	1997	Israel	736	1994	1994	RCT	Age	1.16 (0.83–1.62)
O'Reilly*	2012	Northern Ireland	37059	2001	2004	cohort	Age<br><br>Education:<br>Medium vs low<br>High vs low<br>Medium vs high<br><br>Housing tenure (homeowner vs non)<br><br>Marital status (non-married/non-cohabiting vs married/cohabiting)	0.72 (0.68–0.76)<br><br><br>1.05 (0.93–1.19)<br>1.16 (1.07–1.25)<br>1.10 (0.95–1.27)<br><br>2.14 (2.03–2.27)<br><br>1.72 (1.64–1.81)

Breast cancer screening attendance factors: Systematic review

							Residence (rural vs urban)	1.59 (1.50–1.68)
							No access to vehicle	0.43 (0.41–0.46)
							Long-term limiting illness	0.71 (0.68–0.75)
							Good general health	1.55 (1.46–1.64)
							Religion:<br>Catholic vs none<br>Protestant vs none	 1.40 (1.25–1.47)<br>1.57 (1.46–1.70)
Ouédraogo*	2014	France	13565	2010	2011	cohort	Age	1.02 (0.95–1.10)
							Residence (rural vs urban)	0.80 (0.75–0.86)
							SES:<br>Medium vs low<br>High vs low<br>Medium vs high	 1.08 (0.99–1.18)<br>1.21 (1.11–1.32)<br>1.12 (1.04–1.22)
							Distance to screening centre	0.85 (0.79–0.91)
Peeters*	1994	Netherlands	1863	1992	1992	RCT	Age	0.69 (0.57–0.84)
Pelfrene	1998	Belgium	40713	1992	1992	cohort	NA	NA
Pinckney	2003	USA	41844	1996	1997	retrospective cohort	NA	NA
Pornet*	2010	France	4865	2004	2006	cohort	Age	1.18 (1.04–1.35)
							SES:<br>Medium vs low<br>High vs low<br>Medium vs high	 1.24 (1.10–1.40)<br>1.40 (1.16–1.67)<br>1.12 (0.93–1.35)

							>0 GPs per 100,000 inhabitants	0.96 (0.85–1.08)
							>0 radiologists per 100,000 inhabitants	0.87 (0.72–1.05)
Renshaw*	2010	UK	742786	2004	2007	cohort	Age	1.00 (0.99–1.01)
							SES:	
							Medium vs low	1.59 (1.57–1.61)
							High vs low	2.01 (1.98–2.04)
							Medium vs high	1.26 (1.25–1.28)
							First invitation to screening	0.22 (0.21–0.22)
Richards	2001	UK	5732	1997	1998	RCT	NA	NA
Rodriguez	1995	Spain	1859	1989	1989	cohort	NA	NA
Scaf-Klomp	1995	Netherlands	6898	1975	1990	cohort	NA	NA
Segnan	1998	Italy	8069	1993	1993	RCT	NA	NA
Sim*	2012	Australia	582729	1995	2007	retrospective cohort	Previous result of mammogram	0.87 (0.84–0.89)
Simon	2001	USA	1718	1992	1993	RCT	NA	NA
St-Jacques*	2013	Canada	833856	2006	2008	cohort	Age	1.00 (0.99–1.01)
							SES:	
							Medium vs low	1.16 (1.15–1.18)
							High vs low	1.15 (1.13–1.16)
							Medium vs high	0.98 (0.97–0.99)
							Distance to screening centre	1.02 (1.01–1.03)
Sutradhar	2016	Canada	2389889	2001	2010	retrospective cohort	NA	NA

Breast cancer screening attendance factors: Systematic review

Szczepura*	2008	UK	86211	2001	2004	cohort	Age	0.81 (0.79–0.84)
							SES:	
							Medium vs low	1.81 (1.74–1.88)
							High vs low	2.14 (2.04–2.25)
							Medium vs high	1.19 (1.13–1.24)
Taplin	1994	USA	1322	unclear	unclear	RCT	NA	NA
Tatla*	2003	Canada	57201	1995	2005	retrospective cohort	Age at initial screen	0.93 (0.90–0.97)
							Previous result of mammogram	0.49 (0.46–0.52)
							Non-English language preferred	0.43 (0.41– to 0.46)
							Referral by health professional	1.05 (1.00– to 1.10)
Taylor*	1999	USA	82	1995	1996	RCT	Age	0.24 (0.05–1.13)
Taylor-Phillips*	2013	UK	11664	2012	2012	cohort	Age	1.34 (1.24–1.45)
							SES:	
							Medium vs low	2.16 (1.97–2.37)
							High vs low	2.84 (2.49–3.25)
							Medium vs high	1.32 (1.17–1.48)
							Previous attender	3.32 (3.05–3.61)
Vermeer*	2010	Netherlands	1279982	2007	2008	cohort	Country of origin (immigrant vs non)	2.79 (2.74–2.84)
Vidal	2014	Spain	12475	2011	2011	quasi-experimental	NA	NA
Visser	2005	Netherlands	825523	1995	2002	retrospective cohort	NA	NA

Mottram R, et al. BMJ Open 2022; 11:e046660. doi: 10.1136/bmjopen-2020-046660

Breast cancer screening attendance factors: Systematic review

von Euler-Chelpin*	2008	Denmark	73416	1991	1999	cohort	Age	0.65 (0.62–0.67)
							Country of origin (immigrant vs non)	1.82 (1.68–1.96)
							Education:	
							Medium vs low	1.24 (1.19–1.30)
							High vs low	0.76 (0.66–0.88)
							Medium vs high	0.61 (0.53–0.71)
							Marital status (non-married/non-cohabiting vs married/cohabiting)	2.18 (2.09–2.27)
Wilf-Miron	2011	Israel	157928	2008	2008	retrospective cohort	NA	NA
Williams	1989	UK	392	unclear	unclear	RCT	NA	NA
Yarnall	1993	USA	unclear	1985	1988	case-control	NA	NA
Zackrisson*	2004	Sweden	33627	1990	1993	cohort	Age	0.91 (0.88–1.08)
							Country of origin (immigrant vs non)	2.05 (1.93–2.17)
							Education:	
							Medium vs low	1.07 (1.00–1.14)
							High vs low	1.01 (0.94–1.08)
							Medium vs high	0.94 (0.87–1.02)
							Marital status (non-married/non-cohabiting vs married/cohabiting)	1.75 (1.67–1.84)
							Income:	
							Intermediate vs low	2.08 (1.96–2.19)

							High vs low	2.24 (2.10–2.39)
							Intermediate vs high	1.08 (1.02–1.14)
							Living in crowded housing conditions	0.29 (0.24– to 0.36)
Zidar	2015	Sweden	52541	2011	2012	cross-sectional	NA	NA

SES: socioeconomic status/deprivation
