# Supplementary material for: Factors associated with attendance at screening for breast cancer: a systematic review and meta-analysis
Source: BMJ Open. 2021 Nov 30;11(11):e046660. doi: 10.1136/bmjopen-2020-046660 (PMC8634222; doi:10.1136/bmjopen-2020-046660)
Supplement: Supplementary data [file bmjopen-2020-046660supp005.pdf]

Breast cancer screen attendance factors: Systematic review

Supplementary file E: Studies included in review but not in meta-analyses or narrative synthesis

Author	Publication year	N in analysis	Study design	Analysis status	Explanation
Allgood	2016	22828	RCT	Intervention was confounder	Control-group-only data not calculable for other factors
Barlow	2019	305568	cohort	No useable data	Age categories could not be pooled
Bourmaud	2016	15844	RCT	Intervention was confounder	Control-group-only data not calculable for other factors
Douglas	2016	NA	cohort	No useable data	Percentages reported, but not Ns per category
Finney Rutten	2014	62754	cohort	No useable factors	Age categories could not be pooled
Gatrell	1998	24000	cohort	No useable factor	Reports uptake by health provider characteristic(s)*
Goldzahl	2018	26495	RCT	Intervention was confounder	Control-group-only data not calculable for other factors
Hyndman	2000	5316	cohort	No useable data	Could not isolate data for different factors
Luckman	2019	10063	RCT	Intervention was confounder	Control-group data for age was reported, but categories could not be pooled
Mayer	2000	1562	RCT	Intervention was confounder	Control-group-only data not calculable for other factors
Meldrum	1994	3083	RCT	Intervention was confounder	Control-group-only data not calculable for other factors
Merrick	2015	4427	RCT	Intervention was	Control-group-only data not

Breast cancer screen attendance factors: Systematic review

				confounder	calculable for other factors
Moss	2001	210939	Cohort	No useable data	Age categories could not be pooled
Offman	2013	12929	RCT	Intervention was confounder	Control-group data for age was reported, but categories could not be pooled
Pelfrene	1998	40713	cohort	No useable data	Percentages reported, but not Ns per category
Pinckney	2003	41844	cohort	No useable data	Age data could not be pooled; attendance data by other factors could not be calculated
Richards	2001	5732	RCT	Intervention was confounder	Control-group-only data not calculable for other factors
Rodriguez	1995	1859	cohort	No useable data	Inadequate data reported
Scaf-Klomp	1995	6898	cohort	No useable data	Risk of double-counting participants; time-series data
Segnan	1998	8069	RCT	Intervention was confounder	Control-group-only data not calculable for other factors
Simon	2001	1718	RCT	Intervention was confounder	Control-group-only data not calculable for other factors
Sutradhar	2016	2389889	cohort	No useable data	Does not report data adequate for calculating ORs
Taplin	1994	1322	RCT	Intervention was confounder	Control-group-only data not calculable for other factors
Vidal	2014	12475	quasi-experimental	Intervention was confounder	Control-group-only data not calculable for other factors
Visser	2005	825523	Cohort	No useable	Country of origin

Breast cancer screen attendance factors: Systematic review

				factors	data could not be pooled
Wilf-Miron	2011	157928	cohort	No useable data	Ns and % attendance not reported
Williams	1989	392	RCT	Intervention was confounder	Control-group-only data not calculable for other factors
Yarnall	1993	unclear	case-control	No useable factor	Reports uptake by health provider characteristic(s)*
Zidar	2015	52541	cross-sectional	No useable data	Reports % non-attendance by age group, but no N per category

*Attendance was measured based on a characteristic or behaviour of the medical provider or facility, not a characteristic of the patient, for example, physician’s gender, health centre’s use of a special assessment form, or the social deprivation status of the health centre (rather than the patient).
