# Supplementary material for: Factors associated with attendance at screening for breast cancer: a systematic review and meta-analysis
Source: BMJ Open. 2021 Nov 30;11(11):e046660. doi: 10.1136/bmjopen-2020-046660 (PMC8634222; doi:10.1136/bmjopen-2020-046660)
Supplement: Supplementary data [file bmjopen-2020-046660supp006.pdf]

Supplementary file E: Funnel plots

Funnel Plots (note the central estimates come from fixed effects analyses, as these are the only way to assess bias, but means they will not match with the random effects estimates in the main paper)

Age

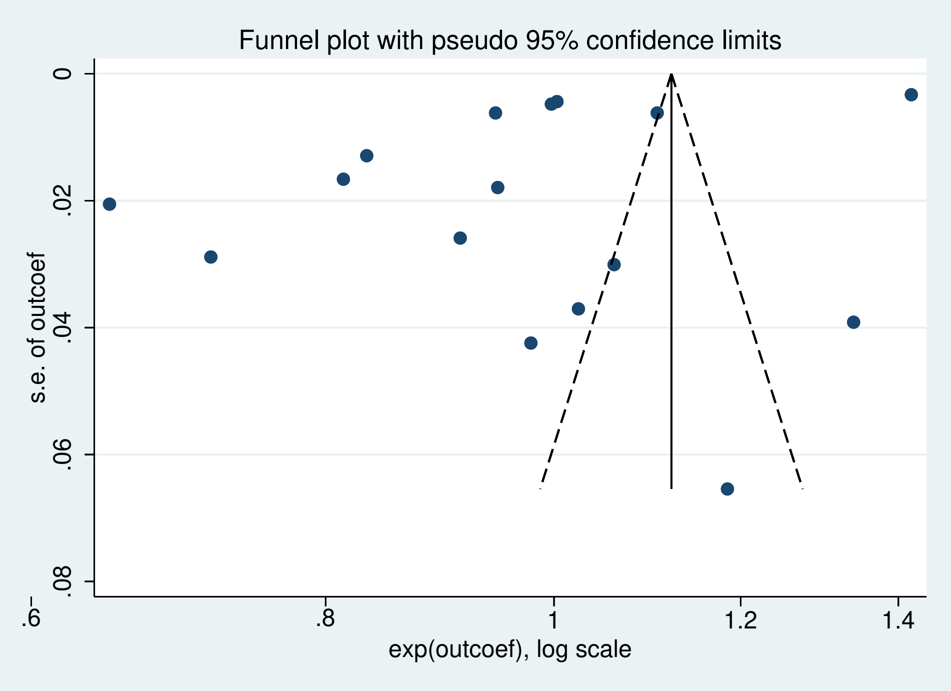

Homeowner

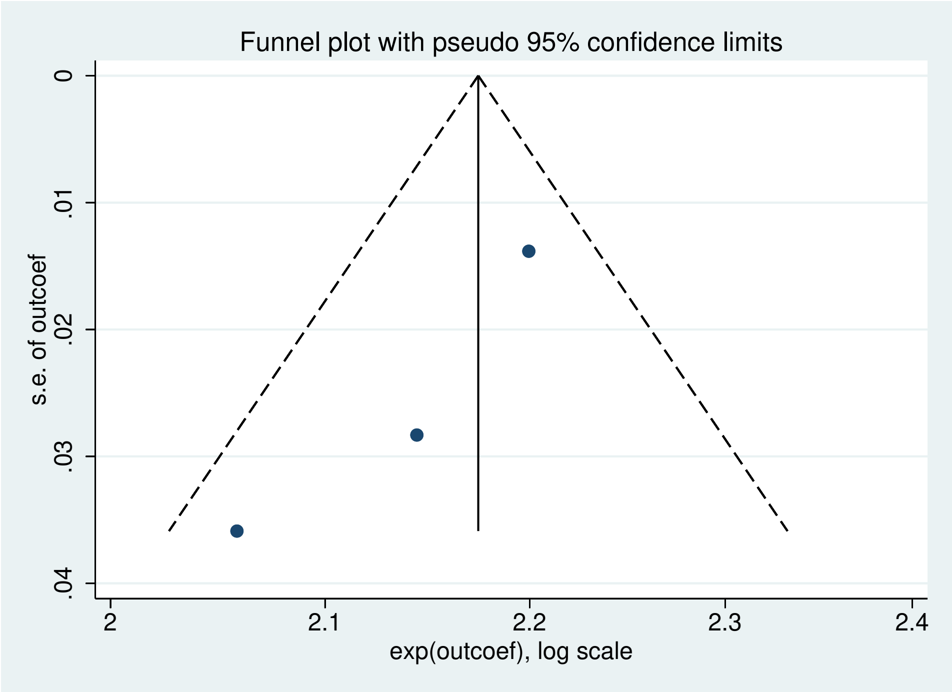

Marital status

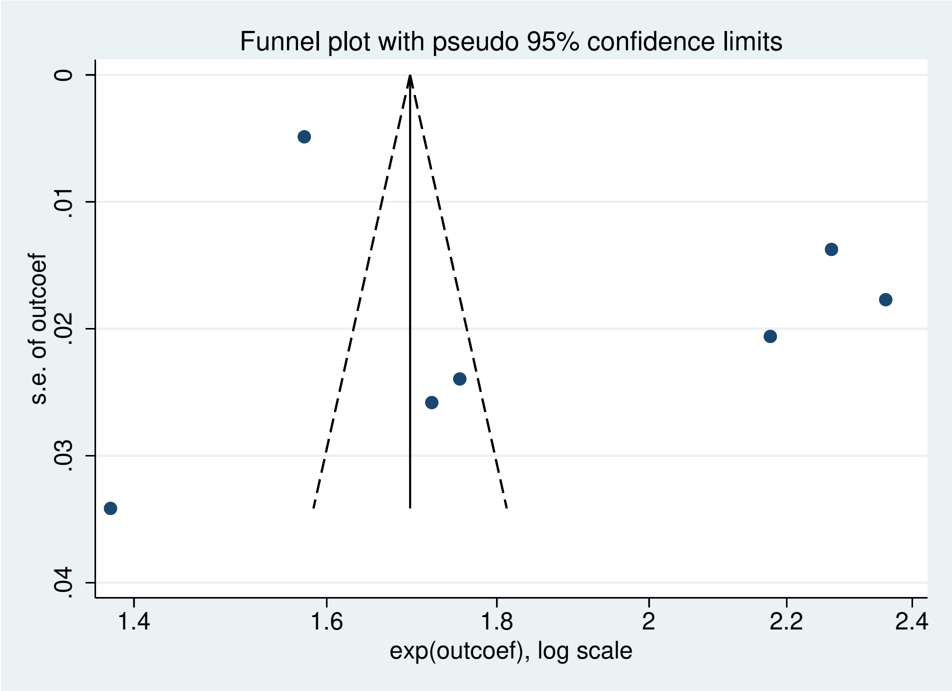

Immigrant status

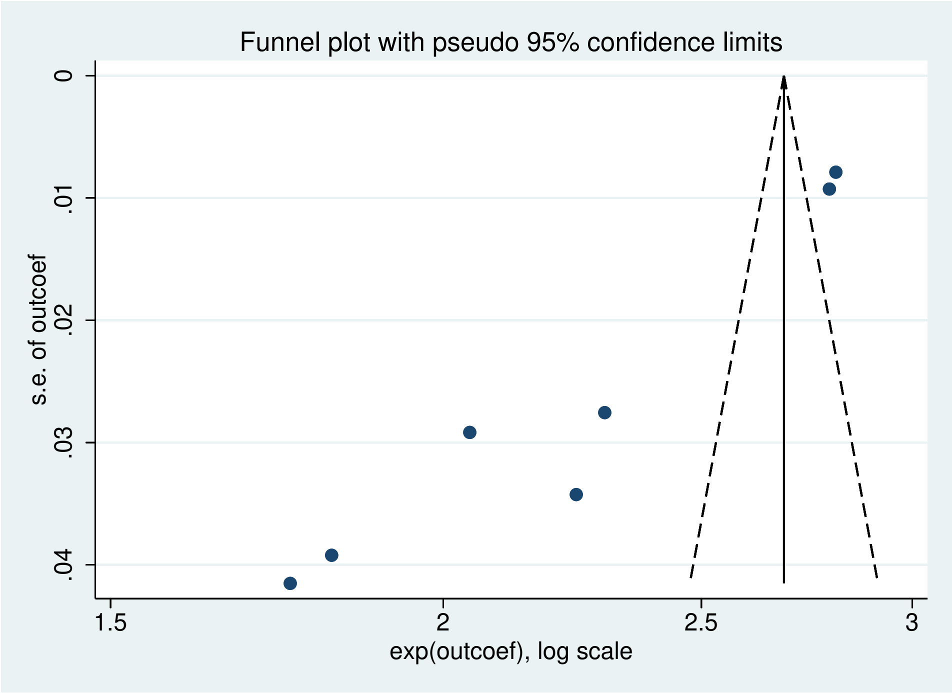

Reattendance

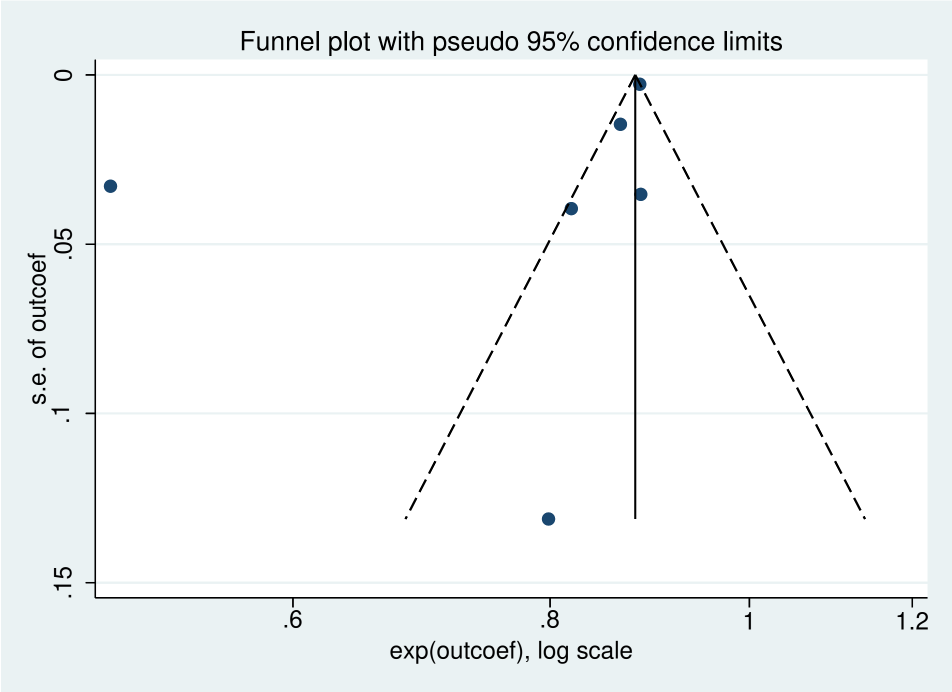

These graphs demonstrate the high level of heterogeneity present between the studies in these analyses.

Age is the only analysis where the studies disagree over the direction of attendance, however the disagreement is among larger studies, suggesting this is unlikely to be associated with biased reporting and instead down to the study heterogeneity. All other analyses, whilst having studies which disagree on the point estimate, have agreement as to which group is more or less likely to attend breast cancer screening.

Overall, we are not concerned about reporting bias.
