# Supplementary material for: Factors associated with attendance at screening for breast cancer: a systematic review and meta-analysis
Source: BMJ Open. 2021 Nov 30;11(11):e046660. doi: 10.1136/bmjopen-2020-046660 (PMC8634222; doi:10.1136/bmjopen-2020-046660)
Supplement: Author's manuscript [file bmjopen-2020-046660.draft_revisions.pdf]

BMJ Open is committed to open peer review. As part of this commitment we make the peer review history of every article we publish publicly available.

When an article is published we post the peer reviewers' comments and the authors' responses online. We also post the versions of the paper that were used during peer review. These are the versions that the peer review comments apply to.

The versions of the paper that follow are the versions that were submitted during the peer review process. They are not the versions of record or the final published versions. They should not be cited or distributed as the published version of this manuscript.

BMJ Open is an open access journal and the full, final, typeset and author-corrected version of record of the manuscript is available on our site with no access controls, subscription charges or pay-per-view fees (http://bmjopen.bmj.com).

If you have any questions on BMJ Open's open peer review process please email info.bmjopen@bmj.com

BMJ Open

Factors associated with attendance at breast cancer screening: a systematic review and meta-analysis

Journal:	*BMJ Open*
Manuscript ID	bmjopen-2020-046660
Article Type:	Original research
Date Submitted by the Author:	05-Nov-2020
Complete List of Authors:	Mottram, Rebecca; University of Warwick Warwick Medical School, Division of Health Sciences<br>Knerr, Wendy; University of Warwick Warwick Medical School, Division of Health Sciences<br>Gallacher, Daniel; University of Warwick Warwick Medical School, Division of Health Sciences<br>Fraser, Hannah; University of Warwick, Warwick Medical School<br>Al-Khudairy, Lena; University of Warwick Warwick Medical School, Division of Health Sciences<br>Ayorinde, Abimbola; University of Warwick Warwick Medical School, Division of Health Sciences<br>Williamson, Sian; University of Warwick Warwick Medical School, Division of Health Sciences<br>Nduka, Chidozie; University of Warwick Warwick Medical School, Division of Health Sciences<br>Uthman, Olalekan; University of Warwick Warwick Medical School, Division of Health Sciences<br>Johnson, Samantha ; University of Warwick, University of Warwick Library<br>Tsertsvadze, Alexander; University of Ottawa Faculty of Medicine, School of Epidemiology and Public Health<br>Stinton, Christopher; University of Warwick Warwick Medical School, Division of Health Sciences<br>Taylor-Phillips, Sian; University of Warwick Warwick Medical School, Division of Health Sciences<br>Clarke, Aileen; University of Warwick Warwick Medical School, Division of Health Sciences
Keywords:	Breast tumours < ONCOLOGY, Breast imaging < RADIOLOGY & IMAGING, Diagnostic radiology < RADIOLOGY & IMAGING, PUBLIC HEALTH

SCHOLARONE™
Manuscripts

I, the Submitting Author has the right to grant and does grant on behalf of all authors of the Work (as defined in the below author licence), an exclusive licence and/or a non-exclusive licence for contributions from authors who are: i) UK Crown employees; ii) where BMJ has agreed a CC-BY licence shall apply, and/or iii) in accordance with the terms applicable for US Federal Government officers or employees acting as part of their official duties; on a worldwide, perpetual, irrevocable, royalty-free basis to BMJ Publishing Group Ltd ("BMJ") its licensees and where the relevant Journal is co-owned by BMJ to the co-owners of the Journal, to publish the Work in this journal and any other BMJ products and to exploit all rights, as set out in our licence.

The Submitting Author accepts and understands that any supply made under these terms is made by BMJ to the Submitting Author unless you are acting as an employee on behalf of your employer or a postgraduate student of an affiliated institution which is paying any applicable article publishing charge ("APC") for Open Access articles. Where the Submitting Author wishes to make the Work available on an Open Access basis (and intends to pay the relevant APC), the terms of reuse of such Open Access shall be governed by a Creative Commons licence – details of these licences and which Creative Commons licence will apply to this Work are set out in our licence referred to above.

Other than as permitted in any relevant BMJ Author's Self Archiving Policies, I confirm this Work has not been accepted for publication elsewhere, is not being considered for publication elsewhere and does not duplicate material already published. I confirm all authors consent to publication of this Work and authorise the granting of this licence.

For peer review only

Factors associated with attendance at breast cancer screening: a systematic review and meta-analysis

Rebecca Mottram[1] (crosbyr4@gmail.com), Wendy Knerr[1] (wendyknerr@gmail.com), Daniel Gallacher[1] (D.Gallacher@warwick.ac.uk), Hannah Fraser[1] (H.Fraser@warwick.ac.uk), Lena Al-Khudairy[1] (Lena.al-Khudairy@warwick.ac.uk), Abimbola Ayorinde[1] (A.Ayorinde.1@warwick.ac.uk), Sian Williamson[1] (S.Z.Williamson@soton.ac.uk), Chidozie Nduka[1] (C.Nduka@warwick.ac.uk), Olalekan A. Uthman[1] (olalekan.uthman@warwick.ac.uk), Samantha Johnson[2] (Samantha.A.Johnson@warwick.ac.uk), Alexander Tsertsvadze[1 3] (a_tsertsvadze@hotmail.com), Chris Stinton[1] (C.Stinton@warwick.ac.uk), Sian Taylor-Phillips[1] (S.Taylor-Phillips@warwick.ac.uk), Aileen Clarke[1]*(Aileen.Clarke@warwick.ac.uk)

[1]Division of Health Sciences, University of Warwick Medical School, Gibbet Hill Campus, Coventry, CV4 7AL, UK.

[2]University of Warwick Library, University of Warwick, Coventry CV4 7AL, United Kingdom

[3]School of Epidemiology and Public Health, Faculty of Medicine, University of Ottawa, Ottawa, Ontario K1G 5Z3, Canada

*Corresponding author: Aileen Clarke, Warwick Medical School, University of Warwick, Coventry, CV4 7AL, 024761 50189, email:aileen.clarke@warwick.ac.uk

Word count: 3691

Keywords: Breast cancer; mammography; mass screening; systematic review

For peer review only

For peer review only - http://bmjopen.bmj.com/site/about/guidelines.xhtml

ABSTRACT

Objective Risks and benefits of population-based breast cancer screening are contested, and attendance varies. This comprehensive systematic review and meta-analysis assesses all patient-level factors associated with routine population breast screening attendance.

Design CINAHL, Cochrane Library, Embase, Medline, OVID, PsycINFO and Web of Science were searched to 26 June 2019. Studies of any design, reporting attendance in relation to at least one patient-level factor were included.

Data synthesis Independent reviewers performed screening, data extraction and quality appraisal. Odds ratios (OR) and 95% confidence intervals (CIs) were calculated for attendance for each factor and random-effects meta-analysis was undertaken where possible.

Results Of 19,776 studies, 331 were assessed at full text and 66 were included. Risk of bias was generally low. Increased attendance was associated with higher socioeconomic status (SES) (n = 10 studies; OR 1.46, 95% CI: 1.19–1.80); higher income (n = 5 studies; OR 1.96, 95% CI: 1.68–2.29); home ownership (n = 3 studies; OR 2.16, 95% CI: 2.08–2.23); being non-immigrant (n = 7 studies; OR 2.23, 95% CI: 2.00–2.48); being married/cohabiting (n = 7 studies; 1.86, 95% CI: 1.58–2.19); and medium (versus low) level of education (n = 6 studies; OR 1.24, 95% CI: 1.09–1.41). Women with previous false-positive results were less likely to re-attend (n = 6 studies; OR 0.77, 95% CI: 0.68–0.88). There were no differences by age group or by rural versus urban residence.

Conclusions Attendance is lower in women with lower income and SES, those who are immigrants, non-homeowners and those with previous false-positive results. Variations in service delivery and screening programmes may influence findings. Our findings are of univariable associations. Underlying causes of lower uptake should be investigated. Improving equity of access in breast screening appears to require concentrating on women facing practical, physical and financial barriers. (CRD42016051597)

For peer review only - http://bmjopen.bmj.com/site/about/guidelines.xhtml

ARTICLE SUMMARY

Strengths and limitations of this study

- Comprehensive systematic review of all patient-level factors associated with attendance at routine population-based breast cancer screening.
- Two reviewers independently conducted all study selection, data extraction and quality appraisal using QUIPS.
- Both observational and experimental designs were included, using control arms of quasi-experimental or randomized designs, and odds ratios were independently recalculated using each study's raw data.
- Limitations: heterogeneity is high partly due to the large size of studies. Studies were separately meta-analysed by study design, and sensitivity analysis was conducted for one study with an extreme effect size.
- Reporting of potential confounders and effect modifiers was highly variable in studies; this was partially mitigated by re-categorizing variables, such as education levels, to harmonize variables across studies where possible.

For peer review only - http://bmjopen.bmj.com/site/about/guidelines.xhtml

INTRODUCTION

Breast cancer is the most common cancer in females worldwide, with 2.1 million cases diagnosed in 2018.[1] In the UK the majority of breast cancer diagnoses (81%) occur in women over the age of 50.[2] Death rates from breast cancer in the UK have fallen from 42.5 per 100,000 women in 2004 to 33.3 in 2017.[3] Population-based breast cancer screening aims to reduce breast cancer mortality. However, there has recently been controversy about the balance of benefits and harms of breast screening.[4, 5] and breast screening programmes have become more aware of the need for promoting informed choice about breast screening.[6, 7]

Attendance at breast screening is not uniform among the eligible population.[8] Ross et al[9] describe attendance at screening as an individual decision (behavioural) which is affected by accessibility of services (structural) and by a woman's immediate surroundings (societal). Characteristics that have been associated with screening attendance can be grouped into a number of categories, related to sociodemographic factors; health status; health behaviours; accessibility and logistics; beliefs, attitudes and knowledge; simple intention to attend; and societal factors including health systems financing and organization.[5, 10-12]

Most reviews of factors associated with breast screening attendance have focused on individual factors.[e.g., 13, 14, 15] We aimed to provide a comprehensive systematic review of all patient-level characteristics associated with the uptake of population-based screening, as an update to a systematic review conducted in 2008.[5] The aim of this review is to inform population-wide breast screening programmes by bringing together all available evidence about who does and does not attend.

METHODS

Protocol and registration

The review was conducted in accordance with pre-specified methods documented in the protocol registered on the 22nd November 2016 in the PROSPERO International Prospective Register of Systematic Reviews database (Supplementary file A).[16]

For peer review only - http://bmjopen.bmj.com/site/about/guidelines.xhtml

Search and Information sources

The Cumulative Index to Nursing and Allied Health Literature (CINAHL), Cochrane Library, Embase, Medline, PsycINFO and Web of Science were searched from inception up to 26 June 2019. The search was developed in Medline using a combination of MeSH headings and free text terms, and adapted for use in the other databases (an example of the search strategy is available in Supplementary file B).

Reference lists of relevant reviews were searched for potentially relevant studies. Experts in the field were contacted to identify potentially relevant studies that had not been identified in the searches.

Eligibility criteria

Primary studies of any design were included if they reported attendance data from routine population-based mammography screening programmes in relation to at least one patient-level factor, and were written in English since 1987 following publication of the Forrest report[17] and the inception of population-based breast screening in the UK. Studies were excluded if they involved self-reported mammography uptake, opportunistic screening programmes, data for only a subgroup of the eligible population (e.g. women in a narrow age range, only immigrants or only rural women), or uptake data by number of invitations sent rather than number of women. Reviews, commentaries, opinions, letters, and non-empirical and qualitative studies were excluded.

Study selection and data extraction process

Pairs of reviewers screened titles and abstracts independently to identify potentially relevant studies. Two reviewers independently assessed full-text studies for formal inclusion/exclusion assessment against pre-defined eligibility criteria. Disagreements were resolved by a consensus between the two reviewers or by help of a third reviewer.

Data from included studies were extracted and then crosschecked by two reviewers independently. The data included the number of women who attended breast cancer screening and the number invited, and data on patient characteristics, including: sociodemographic factors (e.g. age, marital status, educational level, race/ethnicity,

For peer review only - http://bmjopen.bmj.com/site/about/guidelines.xhtml

immigration status); SES factors; beliefs, attitudes and socioemotional factors; health history and behaviours; logistic and accessibility factors (e.g. distance from screening centre).

Risk of bias of included studies

Risk of bias (RoB) of all included studies was appraised by two independent reviewers using the Quality in Prognosis Studies (QUIPS) tool.[18] The QUIPS tool covers six RoB domains (participation, attrition, prognostic factor, confounding factors, outcome measurement and analysis and reporting), each of which includes multiple items that are judged separately. A conclusive judgment for each RoB domain is reached and expressed on a three-grade scale (high, moderate or low RoB).

Synthesis of data

We used raw attendance data to calculate unadjusted odds ratios for each factor. A random effects model-based meta-analysis was conducted for an association between a factor of interest (dichotomous or more categories) and the dichotomous outcome (screening attendance) to generate Mantel-Haenszel odds ratios with 95% confidence intervals, when possible.[19]

In addition to the main meta-analyses, we conducted separate meta-analyses for a) observational studies whose samples were made up only of women who had previously attended screening (hereafter referred to as rescreening studies) and b) intervention studies (quasi-experimental and randomized controlled trials) that reported characteristics separately for intervention and control arms, recording only data for the control group, as their attendance would not be influenced by exposure to an intervention. We also conducted a sensitivity analysis to determine the impact of a study with an extreme effect size[20] on the meta-analysis of SES.

We summarized results narratively if there was inadequate quantitative data for meta-analysis or variables were reported in fewer than three studies.[19]

This review is reported according to PRISMA guidelines (Supplementary file C).[21]

RESULTS

Literature search

For peer review only - http://bmjopen.bmj.com/site/about/guidelines.xhtml

The process of study flow and reasons for exclusion are provided in Figure 1. In brief, the searches of electronic databases identified 11,949 unique publications (after deduplication), of which 11,618 were excluded at the level of abstract/title screening, leaving 331 records for full text review. Of the 331 full texts, 65 unique studies reported in 66 publications were included (see Supplementary file D for list of references).

Study characteristics

Characteristics of all included studies are listed in Supplementary file E. Of the 65 studies included: 48 were observational (43 retrospective cohort, 1 non-experimental, 2 cross-sectional and 2 case-control designs); and 17 were intervention studies (16 randomized controlled trials and 1 quasi-experimental). Sample sizes ranged from 82 to 4.8 million.

The studies were conducted in Europe (n = 40), North America (n = 17), Asia-Pacific (n = 5) and the Middle East (n = 3). The UK had the most studies (n = 16) followed by the USA (n = 12).

We were able to pool data from 28 observational studies (reported in 29 publications) on the attendance at screening in relation to nine factors (age, education, home ownership, immigration status, marital status, results of previous mammogram, rural/urban residence, SES and income). We were only able to pool data from 3 intervention studies, and only for one factor (age).

Thirty-four studies did not provide adequate data for meta-analysis; although four of these studies provided adequate data to calculate odds ratios and confidence intervals, and are narratively reported. The remaining 30 studies reported data that could not be analysed. (Reasons are detailed in Supplementary file F). In brief, 13 of the 30 studies were intervention trials, where data were not in the right format for us to use. The other 16 studies could not be analysed because uptake data were reported by health-provider characteristics rather than patient characteristics, or by number of invitations sent rather than by number of patients; or because the paper reported percentage uptake but not sample sizes per category.

Risk of bias

For peer review only - http://bmjopen.bmj.com/site/about/guidelines.xhtml

RoB across studies was generally low on all domains (Figure 2). For study participation, 68% of studies were considered at low RoB; for attrition, 88%; for outcome measurement, 94%; and for statistical analysis and reporting, 80%. For measurement of variables associated with attendance (prognostic factors), more than half (58%) of studies had a low RoB, while 23% had a high RoB, mostly due to SES being measured at the area level (e.g. neighbourhood) rather than at the individual level. Half of studies (50%) had a low RoB with regard to measuring potential confounders, with around one-quarter (27%) having a moderate risk and just over one-fifth (21%) having a high risk this domain.

Quantitative data analysis (meta-analyses)

Table 1 presents unadjusted odds ratio estimates with their 95% confidence intervals of attendance at breast screening for factors that were reported in 3 or more studies. The analyses gave I^2 values of around 99%, meaning that there was a high level of heterogeneity, except for the analysis of homeowners versus tenants, where the I^2 value was 38.9% (Table 1).

Table 1. Results of meta-analyses

Variables	Number of women (number of studies included)*	Odds ratio of attendance (unadjusted) (95% confidence interval)
**Age** (60—69 vs 50—59)**		
Observational studies†	7,178,063 (16)	0.97 (0.88–1.88)
Intervention studies	2681 (3)	0.78 (0.47–1.31)
**Education level**	421,706 (6)	
medium vs low		1.24 (1.09–1.41)
high vs low		1.11 (0.97–1.26)
**Housing tenure** (homeowner vs tenant/non-owner)	224,786 (3)	2.16 (2.08–2.23)
**Country of origin** (non-immigrants vs immigrants)	2,523,693 (7)	2.23 (2.00–2.48)

For peer review only - http://bmjopen.bmj.com/site/about/guidelines.xhtml

**Income**	1,200,716 (5)	
intermediate vs low		1.96 (1.68–2.29)
high vs low		2.18 (1.86–2.56)‡
**Marital Status** (married/cohabiting vs unmarried/non-cohabiting)	1,309,152 (7)	1.86 (1.58–2.19)
**Residence** (rural vs urban)	78,040(3)	1.12 (0.76–1.66)
**Previous result of mammogram** (rescreening studies only: false positive vs normal)	3,540,953 (6)	0.77 (0.68–0.88)
**Socioeconomic status (SES)**	6,618,903 (10)	
medium vs low		1.46 (1.19–1.80)
high vs low		1.68 (1.36–2.08)

**References for studies pooled for meta-analyses of observational studies are provided in forest plots on the following pages.*
***We focused on the age bands most commonly eligible in population-based programmes and did not analyse odds for those younger than age 50 or older than 69.*
†All results in this table are for observational studies except the data for age, which includes results for the separate meta-analysis of intervention studies.
‡The odds ratios and confidence intervals for income include all relevant observational studies. We also performed a sensitivity analysis by removing the large study from France by DeBorde et. al,[20] which found that women with high or medium SES were both more likely to attend compared to women of lower SES (OR 1.85 (95% CI: 1.53–2.22, p < 0.001), and OR 1.51 (95% CI: 1.26–1.82, p < 0.001).

We compared the odds of attending breast screening by the age bands most commonly eligible for national screening programmes (60—69 and 50—59). There was no significant difference in meta-analyses of observational studies (n = 16, p = 0.631, Figure 3) or intervention trials (n = 3, p = 0.354).

We grouped education data from 6 studies to approximate the United Nations Educational, Scientific and Cultural Organization (UNESCO) 3-level classification: low (≤10 years), middle (11–15 years) and high (>15 years). Compared to women with a low level of education, women with a medium level were more likely to attend (OR 1.24, 95% CI: 1.09–1.41, p = 0.001). Results from comparisons of women with a high level of education versus low or medium levels were not statistically significant (Figure 4).

For peer review only - http://bmjopen.bmj.com/site/about/guidelines.xhtml

The odds of attending breast screening were higher for homeowners than for tenants or non-owners (OR 2.16, 95% CI: 2.08–2.23, p < 0.001, Figure 5).

Meta-analysis of participants' country of origin showed that people born in the study country (non-immigrants) were more likely to attend than immigrants (OR 2.23, 95% CI: 2.00–2.48, p =0.000, Figure 6).

We meta-analysed attendance using two measures of SES. Data for overall SES from 10 studies was grouped into low, intermediate and high categories. Women with an intermediate or high SES were more likely to attend than those with a low SES (intermediate SES OR 1.46, 95% CI: 1.19–1.80, p < 0.001; high SES OR 1.68, 95% CI: 1.36–2.08, p < 0.001, Figure 7). One study from France (DeBorde)[20] (n = 4.8 million) reported that women with a higher SES were less likely to attend than those with either a low or intermediate SES. We conducted a sensitivity analysis excluding that study, but it made very little difference to the odds of attending: women with high or medium SES were both more likely to attend compared to women of lower SES (OR 1.85, 95% CI: 1.53–2.22, p < 0.001), and OR 1.51, 95% CI: 1.26–1.82, p < 0.001), respectively.

Data on income from 5 studies was grouped into low, intermediate and high categories. Women with an intermediate or high income were more likely to attend than those with low income (intermediate vs low income OR 1.96, 95% CI: 1.68–2.29, p < 0.000; high vs low OR 2.18, 95% CI: 1.86–2.56, p < 0.000; high vs intermediate OR 1.11, 95% CI: 0.95–1.30, p = 0.20, Figure 8). For both income and SES there was no significant difference between women at intermediate and high levels, indicating that there was no statistically significant dose response effect for higher SES or income.

Women who were married or cohabiting were more likely to attend than their unmarried or non-cohabiting counterparts (OR 1.86, 95% CI: 1.58–2.19, p <0.001, Figure 9).

We analysed data separately for studies with samples made up only of women who had previously attended breast screening (i.e. rescreening studies). Six of these studies reported data on attendance based on the results of a previous mammogram. Women who had previously received a false positive result were less likely to attend than those with a normal result (OR 0.77, 95% CI: 0.68–0.88, p <0.001, Figure 10).

For peer review only - http://bmjopen.bmj.com/site/about/guidelines.xhtml

There was no statistically significant difference in attendance among women living in rural compared to urban areas (n = 3 studies, OR 1.12, 95% CI: 0.76–1.66, p = 0.557).

Narrative synthesis

Factors that could not be meta-analysed (because they were reported in fewer than three studies or could not be pooled) are reported in Table 2 with odds ratios.

These studies include a variety of factors associated with reduced attendance clustered around sociodemographic, accessibility and logistics factors (living in crowded housing and being unemployed, receiving disability benefits, lack of access to a vehicle), immigration status (being born in another country), and spoken language not English.

Associations with women's health status, behaviours, attitudes and knowledge showed a mixed picture. There was some evidence that good general health, lack of comorbidity and not taking hormone replacement therapy (HRT) were all associated with higher attendance, but studies also reported higher attendance among women with a higher numbers of previous clinic visits, depression, diabetes and heart disease. A previous negative attitude to breast screening, limiting long-term illness, non-work-related stress, low levels of social and emotional support and connection, fewer than one and more than two live births, and current smoking were associated with lower attendance.

Factors that appeared to have no association with attendance included body mass index (BMI), number of siblings, and service provision factors. No difference in women's attendance was found according to availability of general practitioners or radiologists or physician years since graduation, and there were mixed results according to distance to screening centre.

Table 2. Likelihood of attending screening by factors not suitable for meta-analysis in observational studies

Variable	N*	Included studies	Odds ratio (95% confidence interval)
**Less likely to attend**			
No access to vehicle	113773	Jensen 2012b	0.33 (0.32–0.34)
	37059	O'Reilly 2012	0.43 (0.41–0.46)

For peer review only - http://bmjopen.bmj.com/site/about/guidelines.xhtml

Negative attitude about breast screening	600	Kee 1993	0.44 (0.35–0.55)
Receiving disability benefits	885979	Le 2019	0.70 (0.70–0.71)
First invitation to screening	742786	Renshaw 2010	0.22 (0.21–0.22)
Spoken/preferred language not English	18851	Blanchard 2004	0.33 (0.28–0.39)
	57201	Tatla 2003	0.43 (0.41–0.46)
Long-term limiting illness	37059	O'Reilly 2012	0.71 (0.68–0.75)
	113773	Jensen 2015b	0.64 (0.61–0.66)
Smoking (current)	25120	Katz 2018	0.72 (0.65–0.79)
Living in crowded housing conditions	31948	Zackrisson 2004	0.29 (0.24–0.36)
Employment status			
Outside workforce vs employed/self-employed	173158	Le 2019	0.51 (0.50–0.51)
	113773	Jensen 2012b	0.66 (0.64–0.68)
Unemployed vs employed/self-employed	173158	Le 2019	0.47 (0.45–0.49)
	113773	Jensen 2012b	0.41 (0.40–0.43)
Number of childbirths	46041	Lagerlund 2002	
0 vs 1–2			0.44 (0.40–0.48)
3+ vs 1–2			0.81 (0.75–0.87)
No family history of BC	119502	O'Byrne 2000	0.90 (0.86–0.94)
Type of clinic (mobile vs fixed)	119502	O'Byrne 2000	0.93 (0.88–0.98)
Often lonely (vs seldom/never)	11382	Lagerlund 2014	0.74 (0.68–0.79)
Low level of social participation	11380	Lagerlund 2014	0.79 (0.72–0.86)
Low level of social anchorage (e.g. sense of belonging–rootedness in community)	11402	Lagerlund 2014	0.77 (0.68–0.87)
Low level of instrumental support (e.g. access to practical help /support from other people–such as during illness)	11388	Lagerlund 2014	0.85 (0.78–0.93)
Low level of emotional support	11395	Lagerlund 2014	0.78 (0.71–0.85)
Low level of control/mastery over important things in life	11350	Lagerlund 2014	0.69 (0.64–0.75)

For peer review only

Experience non-work-related stress	11378	Lagerlund 2014	0.65 (0.60–0.71)
Cohabiting arrangements (vs with partner only)	11402	Lagerlund 2014	
Living alone			0.62 (0.57–0.68)
Living with partner + children			0.81 (0.74–0.90)
With children only			0.44 (0.38–0.52)
With parents or other			0.61 (0.41–0.92)
Number of children (vs 2 children)	11386	Lagerlund 2014	
1 child			0.82 (0.74–0.91)
3+ children			0.72 (0.65–0.79)
**More likely to attend**			
No comorbidities	76520	Larsen 2018	1.53 (1.46–1.60)
60+ primary care visits during 6-year study period (vs <60)	43968	Katz 2018	2.70 (2.55–2.86)
Depression	38823	Katz 2018	1.12 (1.02–1.23)
Diabetes	9849	Katz 2018	1.25 (1.17–1.33)
Good general health	37059	O'Reilly 2012	1.55 (1.46–1.64)
Heart disease	6501	Katz 2018	1.75 (1.61–1.91)
Not living in capital city (Oslo)	885979	Le 2019	1.94 (1.91–1.97)
Previous attender	11664	Taylor-Phillips 2013	3.32 (3.05–3.61)
Citizen of country	885979	Le 2019	2.88 (2.82–2.94)
Member of majority racial/ethnic group	51241	Beaber 2019	1.19 (1.09–1.28)
	17997	Blanchard 2004	1.70 (1.52–1.89)
Religion			
Catholic vs none	37140	O'Reilly 2012	1.40 (1.25–1.47)
Protestant vs none		O'Reilly 2012	1.57 (1.46–1.70)
Never HRT use	119502	O'Byrne 2000	1.13 (1.09–1.17)
Referral by health professional	56420	Tatla 2003	1.05 (1.00–1.10)
**No difference in attendance or mixed results**			

For peer review only - http://bmjopen.bmj.com/site/about/guidelines.xhtml

BMI	46352	Katz 2018	0.95 (0.87–1.04)
>0 GPs per 100,000 inhabitants	4865	Pornet 2010	0.96 (0.85–1.08)
>0 radiologists per 100,000 inhabitants	4865	Pornet 2010	0.87 (0.72–1.05)
Distance to screening centre	108396	Jensen 2012b	0.86 (0.84–0.88)
	833856	St-Jacques 2013	1.02 (1.01–1.03)
	13565	Ouédraogo 2014	0.85 (0.79–0.91)
No siblings	11409	Lagerlund 2014	0.93 (0.82–1.04)
No children (vs 2 children)	11386	Lagerlund 2014	0.93 (0.82–1.05)
Physician years since graduation	105575	Makedonov 2015	1.03 (0.99–1.06)

**Reflects the number of participants analysed for each factor, which can differ for different factors in the same study depending on data availability.*

DISCUSSION

We undertook a comprehensive review of the current evidence on patient-level factors associated with breast screening attendance. Meta-analyses were performed, where appropriate, to determine the strength of association.

Main findings

In line with other systematic reviews,[13] we found that in general higher SES status, higher income and home ownership (compared to renting) predicted breast screening attendance. However, it appears that women with a higher SES or income were not more likely to attend than those with an intermediate level. We hypothesize that women with a higher SES may be more likely to use alternative screening services (i.e. opportunistic or privately funded screening) compared to women with a low or intermediate SES, thus their attendance would not be apparent in studies using data from national screening programmes. This was suggested as a limitation by many of the included studies in this review, most notably the large study from France[20] (n = 4.8 million), which was the only study to find that women with a higher SES were less likely to attend than those with either a low or intermediate SES. The authors of that study note the high levels of opportunistic screening available to women with a high SES in France. We conducted a sensitivity analysis excluding that study, but it made very little difference to the odds ratios for attendance.

For peer review only - http://bmjopen.bmj.com/site/about/guidelines.xhtml

A medium level of education was also associated with screening attendance when compared to a low level, but a higher level of education was not associated with increased attendance compared to either medium or lower levels. As with the analyses of SES, it is possible that women with the highest levels of education are more likely to use alternative screening services not reflected in data from public screening programmes.

We hypothesized that some variation in relation to education or SES might be due to changes in women's attitudes to breast screening as a result of concerns about its overall benefits,[22, 23] perhaps related to the informed-choice agenda.[7] However, we found no population screening studies investigating this.

Our results also support previous research indicating that marital status is associated with attendance at mammography,[22-26] with women who were married or cohabiting more likely to attend than their unmarried or non-cohabiting counterparts. Previous literature indicates lower uptake among women from minority-ethnic backgrounds.[27, 28] While our data were not sufficient to meta-analyse ethnicity, we did find that immigrant women were less likely to attend screening than non-immigrants.

We did not find a significant effect of age, despite other literature suggesting that older women have lower rates of attendance.[29, 30] There was very high heterogeneity here, with individual large studies finding highly statistically significant results in both directions. We hypothesized that attendance may be higher among older women because they have been invited to breast screening for at least two decades, attendance may have become more routine in this cohort, and perhaps also less likely to be affected by recent debates around the risks and benefits of screening. To explore this, we did a post-hoc analysis of the effect of age on attendance by the year of study completion. We found that older women were more likely to attend compared to younger women in more recent studies (i.e. those completed since 2010), but that the opposite was true in older studies, particularly those published before 2005.

There were no significant differences in attendance by rural versus urban residence.

Women who received a false-positive result at a previous screening were less likely to attend than those with a normal result, confirming previous findings.[31]

For peer review only - http://bmjopen.bmj.com/site/about/guidelines.xhtml

Strengths and limitations

This review had many strengths. The large number of studies included (n = 65) represents a comprehensive overview of available evidence. Studies included in the meta-analysis were judged to have a low risk of bias on most domains and included large numbers of women. At least two reviewers were involved at all stages to reduce the risk of errors and bias. In addition, we excluded studies that relied on self-reported attendance and those that sampled population subgroups.

The main limitation of this review is significant between-study heterogeneity. Although we used random effect models throughout, our results should be considered in light of this. Heterogeneity may in part be due to differences between health systems and the organization of breast screening, as well as differences in the culture and attitudes of the populations served. We conducted sensitivity analysis to determine the impact of a very large study with an extreme effect size[20] on the meta-analysis of SES. For some outcomes (such as age) the heterogeneity encompasses studies with highly significant results in both directions, and here the results of the meta-analysis should be interpreted with great caution. For other variables (such as re-attendance after false positive results) the high I^2 simply reflects that there were very large studies with very small confidence intervals, which all had point estimates of different magnitude in the same direction. Here the meta-analysis results show a consistent effect, with some disagreement between studies on the exact size of effect.

A second limitation is that we extracted univariable associations with uptake. In practice, many of the variables investigated will be highly correlated, and there will be complex interactions and confounding which we have not accounted for. While some studies did report multivariable models, these were varied in structure, methods and variables included, so would have been difficult to combine in any meaningful way. We were therefore unable to undertake multivariable meta-regression analysis, examining the effects of individual attendance factors on overall attendance.

For the studies included in the narrative analysis, large numbers of women were also often involved, but these studies should be treated with caution as they are potentially subject to bias. The risk of confounding was found to be high in these studies using the QUIPs tool.

For peer review only - http://bmjopen.bmj.com/site/about/guidelines.xhtml

However, confounding is inherent in the design of population-based observational and especially ecological designs. Our studies may also suffer from publication bias, which cannot be estimated with corroboratory evidence. We have not included health insurance (or lack of health insurance) as a factor in the narrative analysis because of the problems of comparison between countries.

CONCLUSIONS

A wide variety of factors affect a woman's decision to attend breast screening. Our main findings are that there was a strong relationship with SES, income and education. Based on our current findings, if screening programmes wish to improve equity of access to breast screening services they should concentrate on women facing practical, physical and financial barriers, such as those with lower SES and education levels and also those who have received previous false-positive results. In addition, private and alternative facilities should harmonize data collection to ensure the adequate characterization of screening behaviour.

We found no evidence relating to the effect of the informed choice agenda on breast screening attendance. Ideally, equity of opportunity to make an informed choice needs to be coupled with equity of access once a woman has made the decision. Research in this area would need to systematically measure attitudes and beliefs relating to informed choice across population-based studies. We consider this an important area for future primary research and investigation.

DEVIATIONS FROM STUDY PROTOCOL

To assess RoB, the QUIPS tool was used rather than the Quality Assessment Tool; and for data synthesis, despite significant heterogeneity, meta-analysis was possible for some predictors. In addition, we clarified our inclusion criteria to include only studies with data from routine population-based mammography screening programmes in order to ensure generalizability.

AUTHOR CONTRIBUTIONS

RM conceived the study as part of her PhD Dissertation, and it was further refined in collaboration with AC and STP. AC, CS and WK further developed the inclusion and exclusion criteria. SW undertook database searches and AC, HF, RM, LAK and WK reviewed titles and

For peer review only - http://bmjopen.bmj.com/site/about/guidelines.xhtml

abstracts. Each study retained for full-text review was reviewed by RM and WK. Discrepancies regarding inclusion and exclusion were resolved by AC and CS. RM and WK did data extraction, and data were checked by OU and CN. Studies were critically appraised by AA, AT, CS and WK. Meta-analyses were conducted by DG. Thematic synthesis was done by AC, STP and WK. All authors contributed to the manuscript and approved the final version.

FUNDING

This research was funded by the National Institute for Health Research Collaboration for Leadership in Applied Health Research and Care West Midlands (NIHR CLAHRC WM), now recommissioned as NIHR Applied Research Collaboration West Midlands (NIHR ARC WM). Rebecca Mottram, Lena Al-Khudairy, Sian Taylor-Phillips, Sian Williamson, Hannah Fraser, Chris Stinton, and Aileen Clarke were all supported by the NIHR CLAHRC WM and Wendy Knerr, Abimbola Ayorinde, Aileen Clarke and Lena Al-Khudairy are all partly supported by the NIHR ARC WM.

Olalekan Uthman is supported by the NIHR using Official Development Assistance (ODA) funding. Sian Taylor-Phillips is supported by an NIHR Career Development Fellowship (CDF-2016-09-018). The views expressed in this publication are those of the author(s) and not necessarily those of the UK National Health Service (NHS), the NIHR or the Department of Health and Social Care.

COMPETING INTERESTS

Dr. Mottram reports personal fees from the National Institute for Health Research (NIHR) Centre for Leadership in Applied Research and Health Care (CLARHC) West Midlands during the conduct of the study. Dr. Al-Khudairy reports grants from the NIHR during the conduct of the study. Dr. Taylor-Phillips reports grants from NIHR outside the submitted work. Dr. Clarke reports grants from the NIHR for the NIHR Applied Research Centre (ARC) West Midlands and previously from the NIHR CLARHC West Midlands, which supported her and researchers working on this project. Dr. Clarke also received grants from Public Health England (PHE) outside the submitted work. All other authors report no competing interests.

For peer review only

REFERENCES

1. Bray F, Ferlay J, Soerjomataram I, et al. Global cancer statistics 2018: GLOBOCAN estimates of incidence and mortality worldwide for 36 cancers in 185 countries. *CA Cancer J Clin*. 2018;68(6):394-424.
2. CancerResearch;. Breast cancer incidence (invasive) statistics [Website]. CRUK; 2017 [cited 2017 July]. Available from: http://www.cancerresearchuk.org/health-professional/cancer-statistics/statistics-by-cancer-type/breast-cancer/incidence-invasive#heading-Two.
3. ONS. Incidence of, and mortality from breast cancer in England, 2004 to 2017. Excel spreadsheet. Office for National Statistics; 2018 4 June 2018.
4. Marmot MG, Altman DG, Cameron DA, et al. The benefits and harms of breast cancer screening: an independent review. *Br J Cancer*. 2013;108(11):2205-40.
5. Schueler KM, Chu PW, Smith-Bindman R. Factors associated with mammography utilization: a systematic quantitative review of the literature. *J Womens Health (Larchmt)*. 2008;17(9):1477-98.
6. Reder M, Berens E-M, Spallek J, et al. Development of the Informed Choice in Mammography Screening Questionnaire (IMQ): factor structure, reliability, and validity. *BMC Psychology*. 2019;7(1):17.
7. Martínez-Alonso M, Carles-Lavila M, Pérez-Lacasta MJ, et al. Assessment of the effects of decision aids about breast cancer screening: a systematic review and meta-analysis. *BMJ Open*. 2017;7(10):e016894.
8. Coyle C, Kinnear H, Rosato M, et al. Do women who intermittently attend breast screening differ from those who attend every invitation and those who never attend? *J Med Screen*. 2012;19(2):98-103.
9. Ross NA, Rosenberg MW, Pross DC, et al. Contradictions in women's health care provision: A case study of attendance for breast cancer screening. *Soc Sci Med*. 1994;39(8):1015-25.
10. Crosby R. Predictors of uptake of screening mammography. Coventry: University of Warwick; 2018.
11. Aarts MJ, Voogd AC, Duijm LEM, et al. Socioeconomic inequalities in attending the mass screening for breast cancer in the south of the Netherlands-associations with stage at diagnosis and survival. *Breast Cancer Res Treat*. 2011;128(2):517-25.
12. Ackerson K, Preston SD. A decision theory perspective on why women do or do not decide to have cancer screening: systematic review. *J Adv Nurs*. 2009;65(6):1130-40.
13. Smith D, Thomson K, Bambra C, et al. The breast cancer paradox: A systematic review of the association between area-level deprivation and breast cancer screening uptake in Europe. *Cancer Epidemiol*. 2019;60:77-85.
14. Mathioudakis AG, Salakari M, Pylkkanen L, et al. Systematic review on women's values and preferences concerning breast cancer screening and diagnostic services. *Psychooncology*. 2019;28(5):939-47.
15. Bhargava S, Moen K, Qureshi SA, et al. Mammographic screening attendance among immigrant and minority women: a systematic review and meta-analysis. *Acta Radiol*. 2018;59(11):1285-91.
16. Crosby R, Williamson S, Stinton C, et al. A systematic review to identify the worldwide predictors of breast screening uptake PROSPERO International prospective register of systematic reviews: University of York; 2016 [Available from: http://www.crd.york.ac.uk/PROSPERO/display_record.asp?ID=CRD42016051597.
17. Forrest P. Breast cancer screening. Report to the Health Ministers of England Wales Scotland and N Ireland by a working group chaired by Professor Sir Patrick Forrest. HMSO; 1986.
18. Hayden JA, van der Windt DA, Cartwright JL, et al. Assessing bias in studies of prognostic factors. *Ann Intern Med*. 2013;158(4):280-6.
19. Valentine J, Pigott T, Rothstein H. How many studies do you need? A primer on statistical power for meta-analysis. *Journal of Educational and Behavioural Statistics*. 2010;35(2):215-47.

For peer review only - http://bmjopen.bmj.com/site/about/guidelines.xhtml

20. Deborde T, Chatignoux E, Quintin C, et al. Breast cancer screening programme participation and socioeconomic deprivation in France. *Prev Med*. 2018;115:53-60.
21. Liberati A, Altman DG, Tetzlaff J, et al. The PRISMA Statement for Reporting Systematic Reviews and Meta-Analyses of Studies That Evaluate Health Care Interventions: Explanation and Elaboration. *PLoS Med*. 2009;6(7):e1000100.
22. Aro AR, Koning HJ, Absetz P, et al. Psychosocial predictors of first attendance for organised mammography screening. J Med Screen [Internet]. 1999; 6(2):[82-8 pp.]. Available from: http://onlinelibrary.wiley.com/o/cochrane/clcentral/articles/380/CN-00166380/frame.html.
23. Scaf-Klomp W, van Sonderen FL, Stewart R, et al. Compliance after 17 years of breast cancer screening. *J Med Screen*. 1995;2(4):195-9.
24. Ulcickas Yood M, McCarthy BD, Lee NC, et al. Patterns and characteristics of repeat mammography among women 50 years and older. *Cancer Epidemiol Biomarkers Prev*. 1999;8(7):595-9.
25. Lagerlund M, Sparén P, Thurfjell E, et al. Predictors of non-attendance in a population-based mammography screening programme; socio-demographic factors and aspects of behaviour. *Eur J Can Prev*. 2000;9.
26. Jensen LF, Pedersen AF, Andersen B, et al. Social support and non-participation in breast cancer screening: a Danish cohort study. *Journal of Public Health*. 2016;38(2):335-42.
27. Thomas VN, Saleem T, Abraham R. Barriers to effective uptake of cancer screening among Black and minority ethnic groups. *Int J Palliat Nurs*. 2005;11(11):562, 4-71.
28. Dryden R, Williams B, McCowan C, et al. What do we know about who does and does not attend general health checks? Findings from a narrative scoping review. *BMC Public Health*. 2012;12(1):723.
29. Merrick EL, Hodgkin D, Horgan CM, et al. Testing novel patient financial incentives to increase breast cancer screening. *Am J Manag Care*. 2015;21(11):771-9.
30. Lagerlund M, Maxwell AE, Bastani R, et al. Sociodemographic predictors of non-attendance at invitational mammography screening--a population-based register study (Sweden). *Cancer Causes Control*. 2002;13(1):73-82.
31. Long H, Brooks JM, Harvie M, et al. How do women experience a false-positive test result from breast screening? A systematic review and thematic synthesis of qualitative studies. *Br J Cancer*. 2019;121(4):351-8.

For peer review only

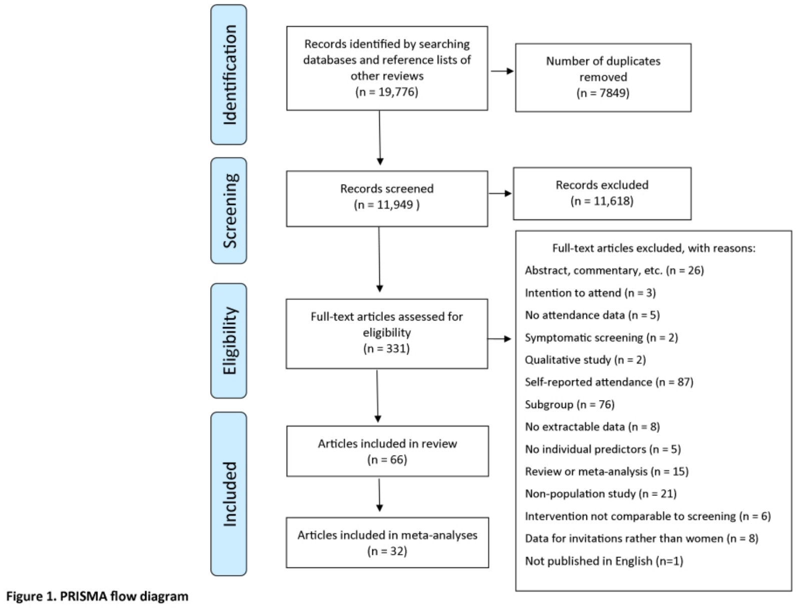

Figure 1. Prisma flow diagram

1237x874mm (72 x 72 DPI)

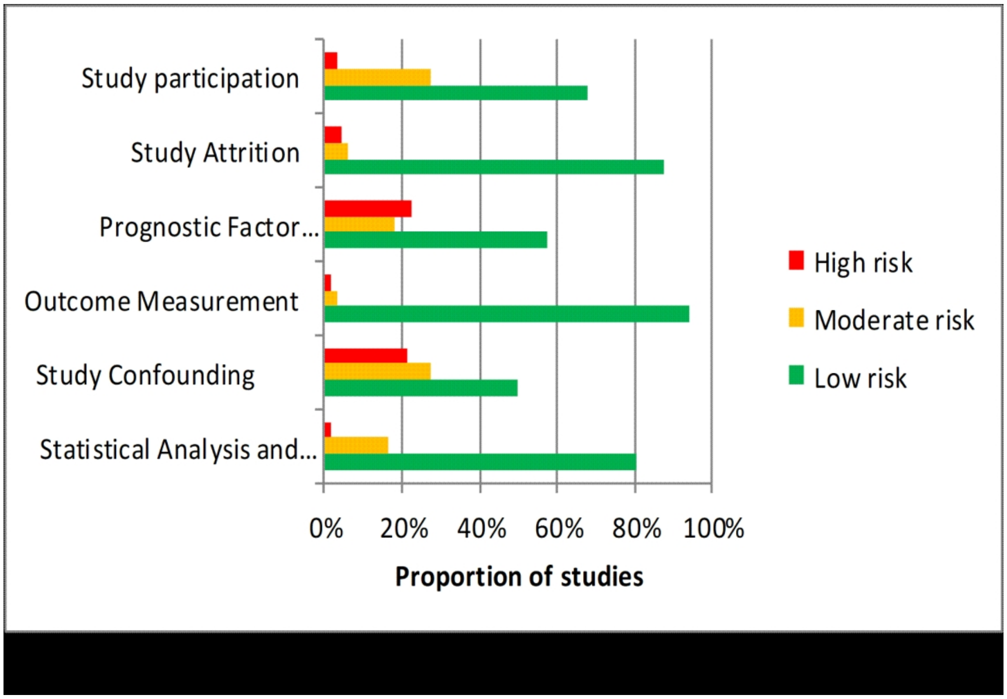

Figure 2. Risk of bias scores

650x449mm (72 x 72 DPI)

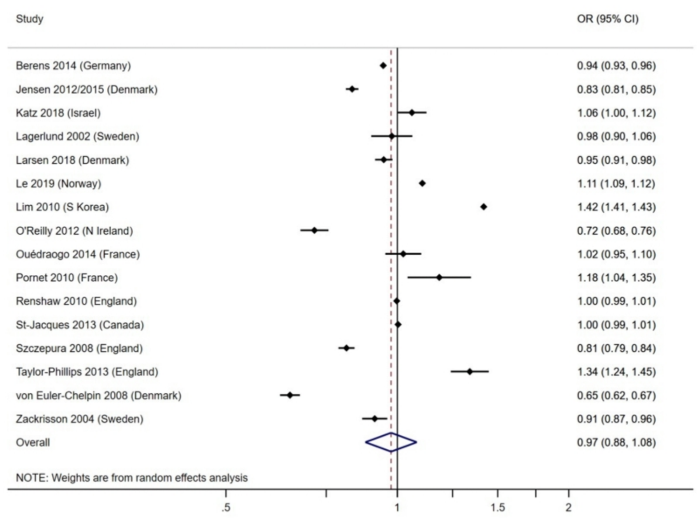

Figure 3. Meta-analysis of attendance by age: OR, age 60–69 vs 50–59 (I^2 = 99.8%, overall p = 0.631)
OR=odds ratio; CI=confidence interval

Figure 3. Meta-analysis of attendance by age

1237x874mm (72 x 72 DPI)

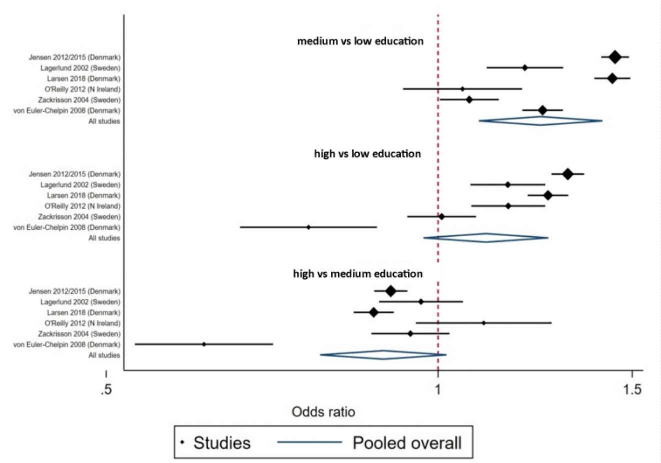

Figure 4. Meta-analysis of attendance by educational level: medium vs low OR 1.24, 95% CI: 1.09–1.41, p = 0.001; high vs low = OR 1.10, 95% CI: 0.97–1.26, p = 0.133
OR=odds ratio; CI=confidence interval

Figure 4. Meta-analysis of attendance by educational level

1237x874mm (72 x 72 DPI)

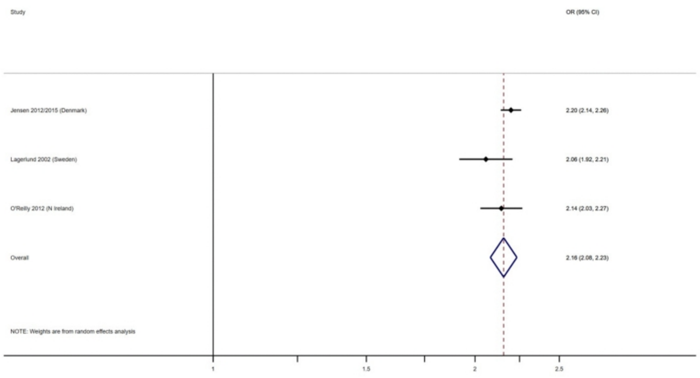

Figure 5. Meta-analysis of attendance by housing tenure: OR, homeowner vs tenant (I^2 = 38.9%, overall p <0.001)
OR=odds ratio; CI=confidence interval

Figure 5. Meta-analysis of attendance by housing tenure

1237x874mm (72 x 72 DPI)

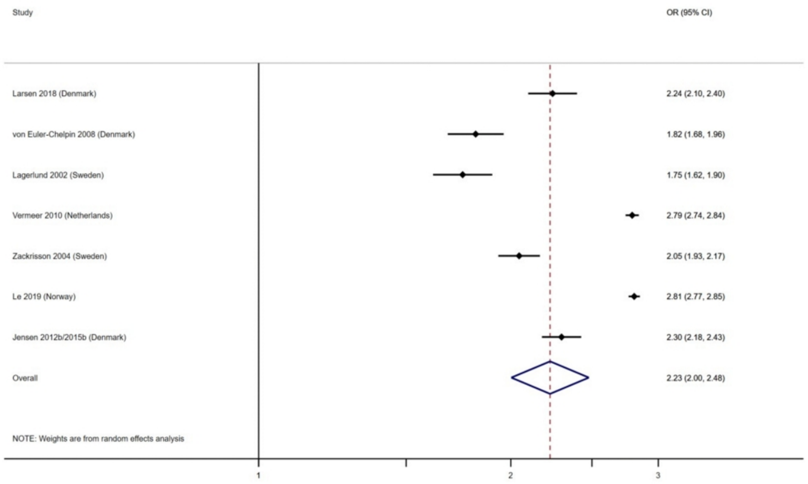

Figure 6. Meta-analysis of attendance by immigrant status: odds ratio, non-immigrant vs immigrant (I^2 = 98.5%, overall p = <0.000)
OR=odds ratio; CI=confidence interval

Figure 6. Meta-analysis of attendanceby immigrant status

1237x874mm (72 x 72 DPI)

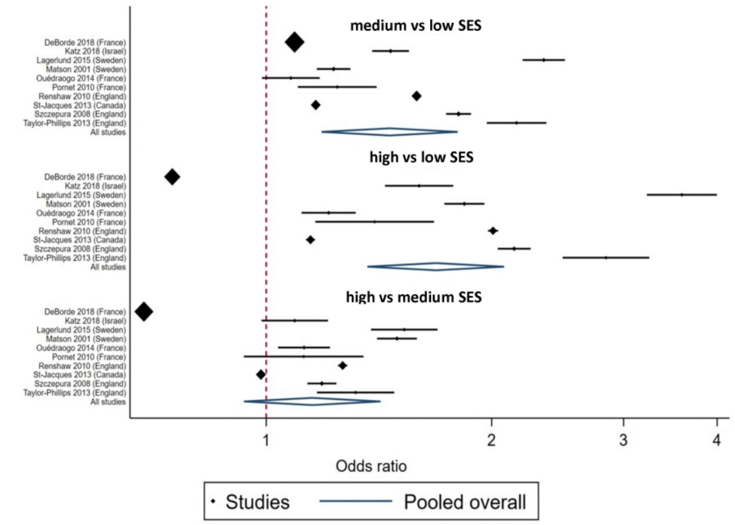

Figure 7. Meta-analysis of attendance by socioeconomic status: medium vs low OR 1.46, 95% CI: 1.19–1.80, p < 0.001; high vs low OR 1.68, 95% CI: 1.36–2.08, p < 0.001)
SES=socioeconomic status; OR=odds ratio; CI=confidence interval

Figure 7. Meta-analysis of attendance by socioeconomic status

1237x874mm (72 x 72 DPI)

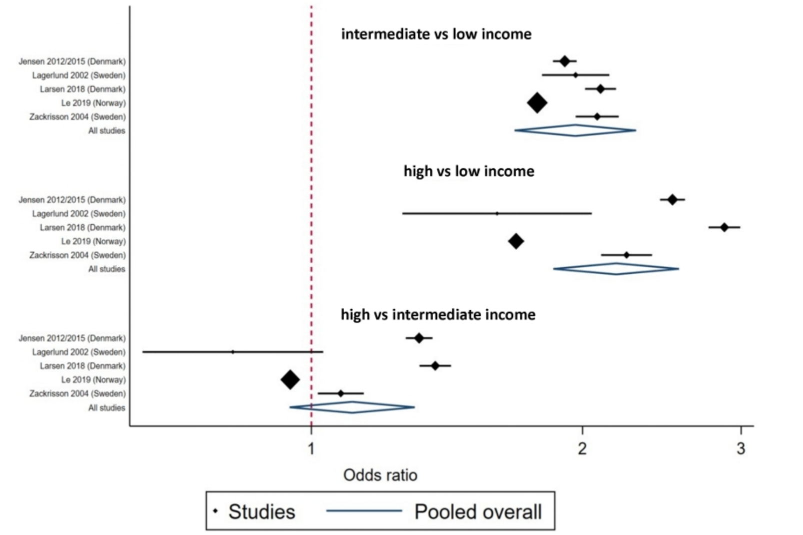

Figure 8. Meta-analysis of attendance by income: intermediate vs low OR 1.96, 95% CI: 1.68–2.29, p < 0.000; high vs low OR 2.18, 95% CI: 1.86–2.56, p < 0.000
OR=odds ratio; CI=confidence interval

Figure 8. Meta-analysis of attendance by income

1237x874mm (72 x 72 DPI)

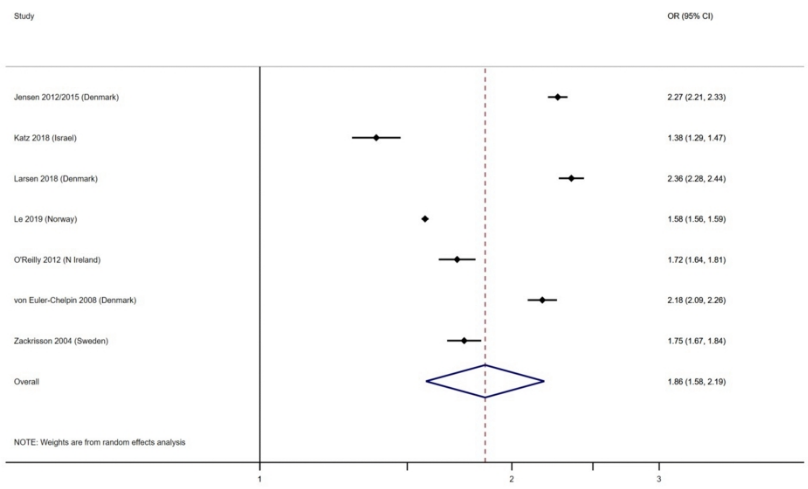

Figure 9. Meta-analysis of attendance by marital status: OR, married/cohabiting vs unmarried/non-cohabiting women (I^2 = 99.5%, overall $p < 0.001$)
OR=odds ratio; CI=confidence interval

Figure 9. Meta-analysis of attendance by marital status

1237x874mm (72 x 72 DPI)

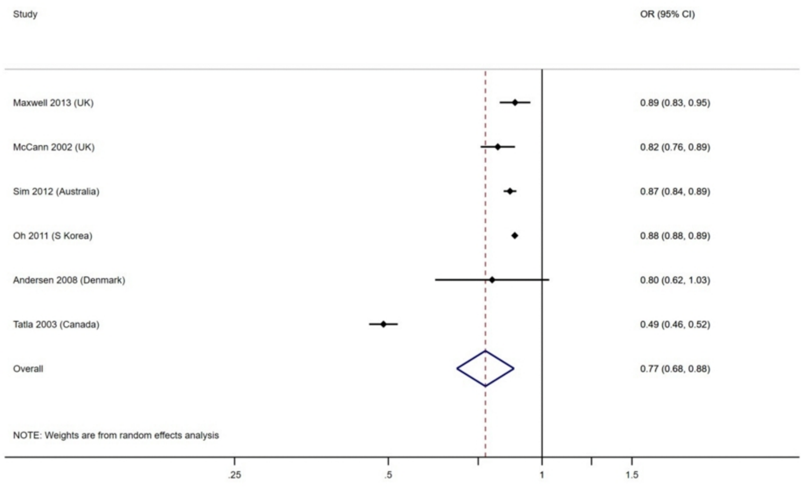

Figure 10. Meta-analysis of re-attendance by result of previous screen: OR, false-positive vs normal results (I^2 = 98.5%, p <0.001)
OR=odds ratio; CI=confidence interval

Figure 10. Meta-analysis of attendance by results of previous screen

1237x874mm (72 x 72 DPI)

NIHR | National Institute for Health Research

PROSPERO
International prospective register of systematic reviews

A systematic review to identify the worldwide predictors of breast screening uptake

Rebecca Crosby, Sian Williamson, Chris Stinton, Aileen Clarke, Sian Taylor-Phillips

Citation

Rebecca Crosby, Sian Williamson, Chris Stinton, Aileen Clarke, Sian Taylor-Phillips. A systematic review to identify the worldwide predictors of breast screening uptake. PROSPERO 2016 CRD42016051597 Available from:
https://www.crd.york.ac.uk/prospero/display_record.php?ID=CRD42016051597

Review question

What are the predictors of breast cancer screening uptake worldwide?

Searches

The electronic databases that will be searched to identify published studies are:
EMBASE (via Ovid), MEDLINE (via Ovid), CINAHL (The Cumulative Index to Nursing and Allied Health Literature), PsycINFO, Cochrane Library (Wiley) including the Cochrane Database of Systematic Reviews and Thomson Reuters Web of Science (all databases including Science Citation Index, Conference Proceedings and Science Citation Index Expanded and Social Sciences Index).
Reference lists of included papers and relevant reviews will be searched for papers that were not identified by the electronic search.
Experts in the field will be contacted to identify further significant papers.

Types of study to be included

Included:Any quantitative study type that mentions uptake rates of breast screening;Study must include at least one predictor of uptake to be included.Excluded:Case studies, editorials, letters and commentaries.

Condition or domain being studied

Breast screening. Predictors of uptake.

Participants/population

Inclusion:
Women of screening age (variable worldwide).
Exclusion:
Women with previously diagnosed breast cancer;
Women attending diagnostic screening;
Non-human studies.

Intervention(s), exposure(s)

Any intervention related to uptake of breast screening will be included in the review. Studies will be included where they mention uptake rates of breast screening - either current, previous or changes. The study must mention at least one predictor variable of uptake.

Comparator(s)/control

Not applicable.

Main outcome(s)

Worldwide predictors of uptake of breast screening.

* Measures of effect

The outcomes will be measured in terms of uptake, i.e. rate or percentages.

Additional outcome(s)

None.

For peer review only

* Measures of effect

Not applicable.

Data extraction (selection and coding)

A two-step process will be used to identify relevant studies at abstract and title stage and then at full text stage using pre-defined screening criteria. Two researchers will screening the titles and abstracts against inclusion and inclusion criteria independently using the results from the search. if a decision cannot be made on the title and abstract a full text review will be performed.
Where there are disagreements between the two researchers, a third reviewer will be contacted until a consensus is reached.
The full texts of the included studies will be obtained and undergo a second screen by two researchers and again any discrepancies resolved by the third reviewer.
Reasons for inclusion and exclusion will be stated where appropriate. The PRISMA flow diagram will be provided in the review.
Data extraction will take place after the full text review and will include:
General - authors, year, publication journal, study title, article type, stated aims, period of study;
Study characteristics - country, setting, screening programme style in this country;
Study design - cohort, case-control, prospective, retrospective, randomised controlled trial, etc.;
Participants - population;
Outcomes - primary and secondary outcomes definitions, validity of measures used, data collection method;
Predictors - number of predictors, type of predictors, definition of predictors;
overall results;
Overview - strengths and limitations of the study, Was the study blinded?; source(s) of research funding, potential conflicts of interest.
The domains involved in data extraction are broad and comprehensive due to the variability of the potential studies to be included within this review. A piloted data extraction form will be used by the two researchers to test. Any discrepancies will be discussed and a third reviewer will be involved where necessary to reach a consensus.

Risk of bias (quality) assessment

Extracted data will be stored in tabular format on Microsoft Access spreadsheet to complete the methodological quality assessment/risk of bias scoring.
Quality assessment of the included studies will be completed using the quality assessment tool.

Strategy for data synthesis

Descriptive analysis will be presented in tabular format to describe the included studies. Significant hetergeneity is expected to be found amongst the included papers considering the differences between screening programmes internationally. Therefore pooling data in a meta-regression would not be appropriate. Instead, a narrative synthesis will be adopted to explain and summarise results by predictor. This narrative synthesis will analyse the population characteristics, predictor variables and their effects on uptake rate.

Analysis of subgroups or subsets

None planned.

Contact details for further information

Rebecca Crosby
R.Crosby@warwick.ac.uk

Organisational affiliation of the review

University of Warwick

Review team members and their organisational affiliations

Miss Rebecca Crosby. University of Warwick
Miss Sian Williamson. University of Warwick
Dr Chris Stinton. University of Warwick
Professor Aileen Clarke. University of Warwick

For peer review only

NIHR | National Institute for Health Research

PROSPERO
International prospective register of systematic reviews

Dr Sian Taylor-Phillips. University of Warwick

Type and method of review
Systematic review

Anticipated or actual start date
21 November 2016

Anticipated completion date
31 July 2017

Funding sources/sponsors
This systematic review presents independent research funded by the NIHR Collaboration for Leadership in Applied Health Research and Care West Midlands (CLAHRC-WM) initiative. The views expressed are those of the author(s) and not necessarily those of the NHS, the NIHR or the Department of Health.

Conflicts of interest
None known

Language
English

Country
England

Stage of review
Review Ongoing

Subject index terms status
Subject indexing assigned by CRD

Subject index terms
Breast Neoplasms; Developed Countries; Developing Countries; Early Detection of Cancer; Early Diagnosis; Healthcare Disparities; Humans; Mass Screening; Patient Acceptance of Health Care; Socioeconomic Factors

Date of registration in PROSPERO
17 November 2016

Date of first submission

Stage of review at time of this submission

Stage	Started	Completed
Preliminary searches	No	No
Piloting of the study selection process	No	No
Formal screening of search results against eligibility criteria	No	No
Data extraction	No	No
Risk of bias (quality) assessment	No	No
Data analysis	No	No

Revision note
Updated the prospero registration to be more accurate. Updated inclusion criteria to only include quantitative studies due to the number of results found after sifting.

For peer review only

NIHR | National Institute for Health Research

PROSPERO
International prospective register of systematic reviews

The record owner confirms that the information they have supplied for this submission is accurate and complete and they understand that deliberate provision of inaccurate information or omission of data may be construed as scientific misconduct.

The record owner confirms that they will update the status of the review when it is completed and will add publication details in due course.

Versions

17 November 2016
23 June 2017

PROSPERO
This information has been provided by the named contact for this review. CRD has accepted this information in good faith and registered the review in PROSPERO. The registrant confirms that the information supplied for this submission is accurate and complete. CRD bears no responsibility or liability for the content of this registration record, any associated files or external websites.

For peer review only

Supplementary table 1: Search strategy used in Medline EMBASE

#	Searches
**1**	Breast*.mp. or exp breast/
**2**	(screen* or “early detection” or mammogram* or “mass screening” or “screening program*” or “direct to consumer” or “health screen*”).mp. [mp=title, abstract, heading word, drug trade name, original title, device manufacturer, drug manufacturer, device trade name, keyword, floating subheading]
**3**	(uptake or adheren* or complian* or “patient acceptance of healthcare” or “patient acceptance” or “patient access” or attend*).mp. [mp=title, abstract, heading word, drug trade name, original title, device manufacturer, drug manufacturer, device trade name, keyword, floating subheading]
**4**	1 and 2 and 3
**5**	Limit 4 to humans
**6**	Limit 5 to English language
**7**	Limit 6 to yr=”1987-Current”

For peer review only

PRISMA 2009 Checklist

Section/topic	#	Checklist item	Reported on page #
**TITLE**			
Title	1	Identify the report as a systematic review, meta-analysis, or both.	1
**ABSTRACT**			
Structured summary	2	Provide a structured summary including, as applicable: background; objectives; data sources; study eligibility criteria, participants, and interventions; study appraisal and synthesis methods; results; limitations; conclusions and implications of key findings; systematic review registration number.	2
**INTRODUCTION**			
Rationale	3	Describe the rationale for the review in the context of what is already known.	4
Objectives	4	Provide an explicit statement of questions being addressed with reference to participants, interventions, comparisons, outcomes, and study design (PICOS).	5
**METHODS**			
Protocol and registration	5	Indicate if a review protocol exists, if and where it can be accessed (e.g., Web address), and, if available, provide registration information including registration number.	4
Eligibility criteria	6	Specify study characteristics (e.g., PICOS, length of follow-up) and report characteristics (e.g., years considered, language, publication status) used as criteria for eligibility, giving rationale.	5
Information sources	7	Describe all information sources (e.g., databases with dates of coverage, contact with study authors to identify additional studies) in the search and date last searched.	5
Search	8	Present full electronic search strategy for at least one database, including any limits used, such that it could be repeated.	Supplementary file B
Study selection	9	State the process for selecting studies (i.e., screening, eligibility, included in systematic review, and, if applicable, included in the meta-analysis).	5
Data collection process	10	Describe method of data extraction from reports (e.g., piloted forms, independently, in duplicate) and any processes for obtaining and confirming data from investigators.	5–6
Data items	11	List and define all variables for which data were sought (e.g., PICOS, funding sources) and any assumptions and simplifications made.	5–6
Risk of bias in individual studies	12	Describe methods used for assessing risk of bias of individual studies (including specification of whether this was done at the study or outcome level), and how this information is to be used in any data synthesis.	6
Summary measures	13	State the principal summary measures (e.g., risk ratio, difference in means).	6
Synthesis of results	14	Describe the methods of handling data and combining results of studies, if done, including measures of consistency (e.g., I^2) for each meta-analysis.	6

For peer review only - http://bmjopen.bmj.com/site/about/guidelines.xhtml

PRISMA 2009 Checklist

Section/topic	#	Checklist item	Reported on page #
Risk of bias across studies	15	Specify any assessment of risk of bias that may affect the cumulative evidence (e.g., publication bias, selective reporting within studies).	6
Additional analyses	16	Describe methods of additional analyses (e.g., sensitivity or subgroup analyses, meta-regression), if done, indicating which were pre-specified.	6
**RESULTS**			
Study selection	17	Give numbers of studies screened, assessed for eligibility, and included in the review, with reasons for exclusions at each stage, ideally with a flow diagram.	7, Figure 1
Study characteristics	18	For each study, present characteristics for which data were extracted (e.g., study size, PICOS, follow-up period) and provide the citations.	Supplementary files D and E
Risk of bias within studies	19	Present data on risk of bias of each study and, if available, any outcome level assessment (see item 12).	8, 13, 14, Figure 2
Results of individual studies	20	For all outcomes considered (benefits or harms), present, for each study: (a) simple summary data for each intervention group (b) effect estimates and confidence intervals, ideally with a forest plot.	Table 1, Figures 3–10, Table 2
Synthesis of results	21	Present results of each meta-analysis done, including confidence intervals and measures of consistency.	Table 1
Risk of bias across studies	22	Present results of any assessment of risk of bias across studies (see Item 15).	8
Additional analysis	23	Give results of additional analyses, if done (e.g., sensitivity or subgroup analyses, meta-regression [see Item 16]).	10, 13–14
**DISCUSSION**			
Summary of evidence	24	Summarize the main findings including the strength of evidence for each main outcome; consider their relevance to key groups (e.g., healthcare providers, users, and policy makers).	13–14
Limitations	25	Discuss limitations at study and outcome level (e.g., risk of bias), and at review-level (e.g., incomplete retrieval of identified research, reporting bias).	14–15
Conclusions	26	Provide a general interpretation of the results in the context of other evidence, and implications for future research.	15–16
**FUNDING**			
Funding	27	Describe sources of funding for the systematic review and other support (e.g., supply of data); role of funders for the systematic review.	16–17

From: Moher D, Liberati A, Tetzlaff J, Altman DG, The PRISMA Group (2009). Preferred Reporting Items for Systematic Reviews and Meta-Analyses: The PRISMA Statement. PLoS Med 6(7): e1000097. doi:10.1371/journal.pmed1000097

For peer review only - http://bmjopen.bmj.com/site/about/guidelines.xhtml

Breast cancer screen attendance factors: Systematic review

Supplementary file D: Studies included in systematic review

Allgood PC, Maxwell AJ, Hudson S, Offman J, Hutchison G, Beattie C, et al. A randomised trial of the effect of postal reminders on attendance for breast screening. British journal of cancer. 2016; 114(2):171-6.

Andersen SB, Vejborg I, von Euler-Chelpin M. Participation behaviour following a false positive test in the Copenhagen mammography screening programme. Acta oncologica (Stockholm, Sweden). 2008; 47(4):550-5.

Barlow WE, Beaber EF, Geller BM, Kamineni A, Zheng Y, Haas JS, et al. Evaluating Screening Participation, Follow-up, and Outcomes for Breast, Cervical, and Colorectal Cancer in the PROSPR Consortium. Journal of the National Cancer Institute. 2020; 112(3):238-46.

Berens EM, Stahl L, Yilmaz-Aslan Y, Sauzet O, Spallek J, Razum O. Participation in breast cancer screening among women of Turkish origin in Germany - a register-based study. BMC women's health. 2014; 14:24.

Blanchard K, Colbert JA, Puri D, Weissman J, Moy B, Kopans DB, et al. Mammographic screening: patterns of use and estimated impact on breast carcinoma survival. Cancer. 2004; 101(3):495-507.

Bourmaud A, Soler-Michel P, Oriol M, Regnier V, Tinquaut F, Nourissat A, et al. Decision aid on breast cancer screening reduces attendance rate: results of a large-scale, randomized, controlled study by the DECIDEO group. Oncotarget. 2016; 7(11):12885-92.

Chiarelli AM, Mai V, Moravan V, Halapy E, Majpruz V, Tatla RK. False-Positive Result and Reattendance in the Ontario Breast Screening Program. Journal of Medical Screening. 2003; 10(3):129-33.

Deborde T, Chatignoux E, Quintin C, Beltzer N, Hamers FF, Rogel A. Breast cancer screening programme participation and socioeconomic deprivation in France. Preventive Medicine. 2018; 115:53-60.

Douglas E, Waller J, Duffy SW, Wardle J. Socioeconomic inequalities in breast and cervical screening coverage in England: are we closing the gap? Journal of Medical Screening. 2016; 23(2):98-103.

Finney Rutten LJ, Ebbert JO, Jacobson DJ, Squiers LB, Fan C, Breitkopf CR, et al. Changes in U.S. Preventive Services Task Force recommendations: effect on mammography screening in Olmsted County, MN 2004-2013. Preventive medicine. 2014; 69:235-8.

Gatrell A, Garnett S, Rigby J, Maddocks A, Kirwan M. Uptake of screening for breast cancer in South Lancashire. Public Health. 1998; 112(5):297-301.

Goldzahl L, Hollard G, Jusot F. Increasing breast-cancer screening uptake: A randomized controlled experiment. J Health Econ. 2018; 58:228-52.

Hyndman JC, Holman CD, Dawes VP. Effect of distance and social disadvantage on the response to invitations to attend mammography screening. J Med Screen. 2000; 7(3):141-5.

For peer review only

Jensen LF, Pedersen AF, Andersen B, Vedsted P. Identifying specific non-attending groups in breast cancer screening--population-based registry study of participation and socio-demography. BMC Cancer. 2012; 12:518, and Jensen LF, Pedersen AF, Andersen B, Vestergaard M, Vedsted P. Non-participation in breast cancer screening for women with chronic diseases and multimorbidity: a population-based cohort study. BMC Cancer. 2015; 15:798.

Katz D, Tengekyon AJ, Kahan NR, Calderon-Margalit R. Patient and physician characteristics affect adherence to screening mammography: A population-based cohort study. Plos One. 2018; 13(3).

Kee F, Telford AM, Donaghy P, O'Doherty A. Enhancing mammography uptake: who do women listen to? European journal of cancer prevention : the official journal of the European Cancer Prevention Organisation (ECP). 1993; 2(1):37-42.

Lagerlund M, Maxwell AE, Bastani R, Thurfjell E, Ekbom A, Lambe M. Sociodemographic predictors of non-attendance at invitational mammography screening--a population-based register study (Sweden). Cancer causes & control: CCC. 2002; 13(1):73-82.

Lagerlund M, Sontrop JM, Zackrisson S. Psychosocial factors and attendance at a population-based mammography screening program in a cohort of Swedish women. BMC women's health. 2014:33.

Lagerlund M, Merlo J, Vicente RP, Zackrisson S. Does the Neighborhood Area of Residence Influence Non-Attendance in an Urban Mammography Screening Program? A Multilevel Study in a Swedish City. PLoS One. 2015; 10(10):e0140244.

Larsen SH, Virgilsen LF, Kristiansen BK, Andersen B, Vedsted P. Strong association between cervical and breast cancer screening behaviour among Danish women; A register-based cohort study. Prev Med Rep. 2018; 12:349-54.

Le M, Hofvind S, Tsuruda K, Braaten T, Bhargava S. Lower attendance rates in BreastScreen Norway among immigrants across all levels of socio-demographic factors: a population-based study. Journal of Public Health (Germany). 2019; 27(2):229-40.

Leung J, Macleod C, McLaughlin D, Woods LM, Henderson R, Watson A, et al. Screening mammography uptake within Australia and Scotland in rural and urban populations. Prev Med Rep. 2015; 2:559-62.

Lim SM, Lee HY, Choi KS, Jun JK, Park EC, Kim Y, et al. Trends of mammography use in a national breast cancer screening program, 2004-2008. Cancer research and treatment : official journal of Korean Cancer Association. 2010; 42(4):199-202.

Luckmann R, Costanza ME, White MJ, Frisard CF, Rosal M, Sama S, et al. A 4-year randomized trial comparing three outreach interventions to promote screening mammograms. Transl Behav Med. 2019; 9(2):328-35.

Makedonov I, Gu S, Paszat LF. Organized breast screening improves reattendance compared to physician referral: A case control study. BMC Cancer. 2015; 15 (1) (no pagination)(315).

For peer review only

Matson S, Andersson I, Berglund G, Janzon L, Manjer J. Nonattendance in mammographic screening: a study of intraurban differences in Malmo, Sweden, 1990-1994. Cancer detection and prevention. 2001; 25(2):132-7.

Maxwell AJ, Beattie C, Lavelle J, Lyburn I, Sinnatamby R, Garnett S, et al. The effect of false positive breast screening examinations on subsequent attendance: retrospective cohort study. J Med Screen. 2013; 20(2):91-8.

Mayer JA, Lewis EC, Slymen DJ, Dullum J, Kurata H, Holbrook A, et al. Patient reminder letters to promote annual mammograms: a randomized controlled trial. Preventive Medicine. 2000; 31(4):315-22.

McCann J, Stockton D, Godward S. Impact of false-positive mammography on subsequent screening attendance and risk of cancer. Breast Cancer Research. 2002; 4 (5) (no pagination)(R11).

Meldrum P, Turnbull D, Dobson HM, Colquhoun C, Gilmour WH, McIlwaine GM. Tailored written invitations for second round breast cancer screening: a randomised controlled trial. J Med Screen. 1994; 1(4):245-8.

Merrick EL, Hodgkin D, Horgan CM, Lorenz LS, Panas L, Ritter GA, et al. Testing novel patient financial incentives to increase breast cancer screening. Am J Manag Care. 2015; 21(11):771-9.

Moss SM, Brown J, Garvican L, Coleman DA, Johns LE, Blanks RG, et al. Routine breast screening for women aged 65-69: results from evaluation of the demonstration sites. British journal of cancer. 2001; 85(9):1289-94.

O'Byrne AM, Kavanagh AM, Ugoni A, Diver F. Predictors of non-attendance for second round mammography in an Australian mammographic screening programme. J Med Screen. 2000; 7(4):190-4.

Offman J, Wilson M, Lamont M, Birke H, Kutt E, Marriage S, et al. A randomised trial of weekend and evening breast screening appointments. British journal of cancer. 2013; 109(3):597-602.

Oh D, Jung DW, Jun JK, Jung KW, Lee HY, Park EC, et al. On-schedule mammography rescreening in the National Cancer Screening Program for breast cancer in Korea. Asian Pacific journal of cancer prevention : APJCP. 2011; 12(11):2865-70.

Ore L, Hagoel L, Shifroni G, Rennert G. Compliance with mammography screening in Israeli women: the impact of a pre-scheduled appointment and of the letter-style. Israel journal of medical sciences. 1997; 33(2):103-11.

O'Reilly D, Kinnear H, Rosato M, Mairs A, Hall C. Using record linkage to monitor equity and variation in screening programmes. BMC medical research methodology. 2012; 12:59.

Ouédraogo S, Dabakuyo-Yonli TS, Roussot A, Pornet C, Sarlin N, Lunaud P, et al. European transnational ecological deprivation index and participation in population-based breast cancer screening programmes in France. Preventive Medicine. 2014; 63:103-8.

For peer review only

Breast cancer screen attendance factors: Systematic review

Peeters PH, Beckers CG, Hogervorst JM, Collette HJ. Effect on breast cancer screening response in The Netherlands of inviting women for an additional scientific investigation. Journal of epidemiology and community health. 1994; 48(2):175-7.

Pelfrene E, Bleyen L, De Backer G. Uptake in breast cancer screening: A sociogeographical analysis. The European Journal of Public Health. 1998; 8.

Pinckney RG, Geller BM, Burman M, Littenberg B. Effect of false-positive mammograms on return for subsequent screening mammography. The American journal of medicine. 2003; 114(2):120-5.

Pornet C, Dejardin O, Morlais F, Bouvier V, Launoy G. Socioeconomic and healthcare supply statistical determinants of compliance to mammography screening programs: A multilevel analysis in Calvados, France. Cancer Epidemiology. 2010; 34(3):309-15.

Renshaw C, Jack RH, Dixon S, Moller H, Davies EA. Estimating attendance for breast cancer screening in ethnic groups in London. BMC public health. 2010; 10:157.

Richards SH, Bankhead C, Peters TJ, Austoker J, Hobbs FD, Brown J, et al. Cluster randomised controlled trial comparing the effectiveness and cost-effectiveness of two primary care interventions aimed at improving attendance for breast screening. J Med Screen. 2001; 8(2):91-8.

Rodriguez C, Plasencia A, Schroeder DG. Predictive factors of enrollment and adherence in a breast cancer screening program in Barcelona (Spain). Social Science & Medicine. 1995; 40(8):1155-60.

Romaire MA, Bowles EJ, Anderson ML, Buist DS. Comparative effectiveness of mailed reminder letters on mammography screening compliance. Prev Med. 2012; 55(2):127-30.

Scaf-Klomp W, van Sonderen FL, Stewart R, van Dijck JA, van den Heuvel WJ. Compliance after 17 years of breast cancer screening. Journal of medical screening. 1995; 2(4):195-9.

Segnan N, Senore C, Giordano L, Ponti A, Ronco G. Promoting participation in a population screening program for breast and cervical cancer: a randomized trial of different invitation strategies. Tumori. 1998; 84(3):348-53.

Sim MJ, Siva SP, Ramli IS, Fritschi L, Tresham J, Wylie EJ. Effect of false-positive screening mammograms on rescreening in Western Australia. The Medical journal of Australia. 2012; 196(11):693-5.

Simon MS, Gimotty PA, Moncrease A, Dews P, Burack RC. The effect of patient reminders on the use of screening mammography in an urban health department primary care setting. Breast cancer research and treatment. 2001; 65(1):63-70.

St-Jacques S, Philibert MD, Langlois A, Daigle JM, Pelletier E, Major D, et al. Geographic access to mammography screening centre and participation of women in the Quebec Breast Cancer Screening Programme. Journal of epidemiology and community health. 2013; 67(10):861-7.

Sutradhar R, Gu S, Glazier RH, Paszat LF. The association between visiting a primary care provider and uptake of periodic mammograms as women get older. J Med Screen. 2016; 23(2):83-8.

For peer review only

Breast cancer screen attendance factors: Systematic review

Szczepura A, Price C, Gumber A. Breast and bowel cancer screening uptake patterns over 15 years for UK south Asian ethnic minority populations, corrected for differences in socio-demographic characteristics. BMC public health. 2008; 8:346.

Taplin SH, Anderman C, Grothaus L, Curry S, Montano D. Using physician correspondence and postcard reminders to promote mammography use. American journal of public health. 1994; 84(4):571-4.

Tatla RK, Paszat LF, Bondy SJ, Chen Z, Chiarelli AM, Mai V. Socioeconomic status & returning for a second screen in the Ontario breast screening program. Breast (Edinburgh, Scotland). 2003; 12(4):237-46.

Taylor V, Thompson B, Lessler D, Yasui Y, Montano D, Johnson KM, et al. A clinic-based mammography intervention targeting inner-city women. Journal of general internal medicine. 1999; 14(2):104-11.

Taylor-Phillips S, O'Sullivan E, Kearins O, Parsons H, Clarke A. The effects of a UK review of Breast Cancer Screening on Uptake: an observational before/after study. J Med Screen. 2013; 20(2):86-90.

Vermeer B, Van den Muijsenbergh ME. The attendance of migrant women at the national breast cancer screening in the Netherlands 1997-2008. European journal of cancer prevention : the official journal of the European Cancer Prevention Organisation (ECP). 2010; 19(3):195-8.

Vidal C, Garcia M, Benito L, Mila N, Binefa G, Moreno V. Use of text-message reminders to improve participation in a population-based breast cancer screening program. Journal of medical systems. 2014; 38(9):118.

Visser O, van Peppen AM, Ory FG, van Leeuwen FE. Results of breast cancer screening in first generation migrants in Northwest Netherlands. European journal of cancer prevention : the official journal of the European Cancer Prevention Organisation (ECP). 2005; 14(3):251-5.

von Euler-Chelpin M, Olsen AH, Njor S, Vejborg I, Schwartz W, Lynge E. Socio-demographic determinants of participation in mammography screening. International journal of cancer. 2008; 122(2):418-23.

Wilf-Miron R, Peled R, Yaari E, Vainer A, Porath A, Kokia E. The association between socio-demographic characteristics and adherence to breast and colorectal cancer screening: Analysis of large sub populations. BMC Cancer. 2011; 11(1):376.

Williams EM, Vessey MP. Randomised trial of two strategies offering women mobile screening for breast cancer. BMJ (Clinical research ed). 1989; 299(6692):158-9.

Yarnall KS, Michener JL, Broadhead WE, Tse CK. Increasing compliance with mammography recommendations: health assessment forms. The Journal of family practice. 1993; 36(1):59-64.

Zackrisson S, Lindström M, Moghaddassi M, Andersson I, Janzon L. Social predictors of non-attendance in an urban mammographic screening programme: A multilevel analysis. Scandinavian Journal of Social Medicine. 2007; 35(5):548-54.

For peer review only

Breast cancer screen attendance factors: Systematic review

Zidar MN, Larm P, Tillgren P, Akhavan S. Non-attendance of mammographic screening: the roles of age and municipality in a population-based Swedish sample. Intern. 2015; 14:157.

For peer review only

Breast cancer screen attendance factors: Systematic review

Supplementary file E: Characteristics of included studies

*indicates studies that could be included in meta-analysis or narrative analysis

Author	Publication year	Study country	N in analysis	Study start	Study end	Study design
Allgood	2016	UK	22828	unclear	unclear	RCT
Andersen*	2008	Denmark	22653	1999	2001	retrospective cohort
Barlow	2019	USA	305568	2012	2013	cohort
Berens*	2014	Germany	423649	2010	2011	cohort
Blanchard*	2004	USA	18851	1985	2002	retrospective cohort
Bourmaud	2016	France	15844	2009	2009	RCT
Chiarelli*	2003	Canada	125250	1990	1995	retrospective cohort
DeBorde*	2018	France	4805390	2013	2014	cohort
Douglas	2016	UK	NA	2012	2012	cohort
Gatrell	1998	UK	24000	1988	1995	cohort
Goldzahl	2018	France	26495	2015	2015	RCT
Hyndman	2000	Australia	5968	1991	1996	retrospective cohort
Jensen*	2012b/2015b	Denmark	144264	2008	2009	cohort
Katz*	2018	Israel	44318	2008	2014	cohort
Kee*	1993	Northern Ireland	600	1991	1991	cross-sectional
Lagerlund*	2002	Sweden	46041	1988	1997	cohort
Lagerlund	2014	Sweden	11400	1992	2009	cohort
Lagerlund*	2015	Sweden	46041	2005	2009	cohort
Larsen*	2018	Denmark	91517	2008	2009	cohort
Le*	2019	Norway	885979	1996	2015	cohort
Leung*	2015	UK	27416	2008	20101	cohort
Lim*	2010	South Korea	3705246	2008	2008	cohort
Luckman	2019	USA	10063	2010	2014	RCT
Makedonov*	2015	Canada	105665	2005	2011	case-control
Matson*	2001	Sweden	32605	1990	1994	cohort
Maxwell*	2013	UK	253017	2005	2008	retrospective cohort
Mayer	2000	USA	1562	1995	1998	RCT
McCann*	2002	UK	113409	1989	1991	retrospective cohort
Meldrum	1994	UK	3083	1992	1993	RCT
Merrick	2015	USA	4427	2010	2012	RCT
Moss	2001	UK	210939	1996	unclear	retrospective cohort
O'Byrne*	2000	Australia	119502	1995	1996	retrospective cohort
Offman	2013	UK	12929	2010	2011	RCT
Oh*	2011	South Korea	2511976	2005	2008	retrospective cohort
Ore*	1997	Israel	736	1994	1994	RCT
O'Reilly*	2012	Northern Ireland	37059	2001	2004	cohort
Ouédraogo*	2014	France	13565	2010	2011	cohort
Peeters*	1994	Netherlands	1863	1992	1992	RCT

For peer review only

Breast cancer screen attendance factors: Systematic review

Pelfrene	1998	Belgium	40713	1992	1992	cohort
Pinckney	2003	USA	41844	1996	1997	retrospective cohort
Pornet*	2010	France	4865	2004	2006	cohort
Renshaw*	2010	UK	742786	2004	2007	cohort
Richards	2001	UK	5732	1997	1998	RCT
Rodriquez	1995	Spain	1859	1989	1989	cohort
Romaire	2012	USA	48583	2005	2009	pretest-posttest
Rutten	2014	USA	62754	2004	2005	cohort
Scaf-Klomp	1995	Netherlands	6898	1975	1990	cohort
Segnan	1998	Italy	8069	1993	1993	RCT
Sim*	2012	Australia	582729	1995	2007	retrospective cohort
Simon	2001	USA	1718	1992	1993	RCT
St-Jacques*	2013	Canada	833856	2006	2008	cohort
Sutradhar	2016	Canada	2389889	2001	2010	retrospective cohort
Szczepura*	2008	UK	86211	2001	2004	cohort
Taplin	1994	USA	1322	unclear	unclear	RCT
Tatla*	2003	Canada	57201	1995	2005	retrospective cohort
Taylor*	1999	USA	82	1995	1996	RCT
Taylor-Phillips*	2013	UK	11664	2012	2012	cohort
Vermeer*	2010	Netherlands	1279982	2007	2008	cohort
Vidal	2014	Spain	12475	2011	2011	quasi-experimental
Visser	2005	Netherlands	825523	1995	2002	retrospective cohort
von Euler-Chelpin*	2008	Denmark	73416	1991	1999	cohort
Wilf-Miron	2011	Israel	157928	2008	2008	retrospective cohort
Williams	1989	UK	392	unclear	unclear	RCT
Yarnall	1993	USA	unclear	1985	1988	case-control
Zackrisson*	2004	Sweden	33627	1990	1993	cohort
Zidar	2015	Sweden	52541	2011	2012	cross-sectional

For peer review only

Breast cancer screen attendance factors: Systematic review

Supplementary file F: Studies included in review but not in meta-analyses or narrative synthesis

Author	Publication year	N in analysis	Study design	Analysis status	Explanation
Allgood	2016	22828	RCT	Intervention was confounder	Control-group-only data not calculable for other factors
Barlow	2019	305568	cohort	No useable data	Age categories could not be pooled
Bourmaud	2016	15844	RCT	Intervention was confounder	Control-group-only data not calculable for other factors
Douglas	2016	NA	cohort	No useable data	Percentages reported, but not Ns per category
Gatrell	1998	24000	cohort	No useable factor	Reports uptake by health provider characteristic(s)*
Goldzahl	2018	26495	RCT	Intervention was confounder	Control-group-only data not calculable for other factors
Lagerlund	2014	11400	cohort	Include in Table 2??	??
Luckman	2019	10063	RCT	Intervention was confounder	Control-group data for age was reported, but categories could not be pooled
Mayer	2000	1562	RCT	Intervention was confounder	Control-group-only data not calculable for other factors
Meldrum	1994	3083	RCT	Intervention was confounder	Control-group-only data not calculable for other factors
Merrick	2015	4427	RCT	Intervention was confounder	Control-group-only data not calculable for other factors
Moss	2001	210939	Cohort	No useable data	Age categories could not be pooled
Offman	2013	12929	RCT	Intervention was	Control-group data for age was

For peer review only

				confounder	reported, but categories could not be pooled
Pelfrene	1998	40713	cohort	No useable data	Percentages reported, but not Ns per category
Pinckney	2003	41844	cohort	No useable data	Age data could not be pooled; attendance data by other factors could not be calculated
Richards	2001	5732	RCT	Intervention was confounder	Control-group-only data not calculable for other factors
Rodriquez	1995	1859	cohort	No useable data	Inadequate data reported
Romaire	2012	48583	non-experimental	No useable data	Reports attendance by type of invitation letter, based on N of letters sent (not N of women)
Rutten	2014	62754	cohort	No useable factors	Age categories could not be pooled
Scaf-Klomp	1995	6898	cohort	No useable data	Risk of double-counting participants; time-series data
Segnan	1998	8069	RCT	Intervention was confounder	Control-group-only data not calculable for other factors
Simon	2001	1718	RCT	Intervention was confounder	Control-group-only data not calculable for other factors
Sutradhar	2016	2389889	cohort	No useable data	Does not report data adequate for calculating ORs
Taplin	1994	1322	RCT	Intervention was confounder	Control-group-only data not calculable for other factors
Vidal	2014	12475	quasi-experimental	Intervention was	Control-group-only data not

For peer review only

				confounder	calculable for other factors
Visser	2005	825523	Cohort	No useable factors	Country of origin data could not be pooled
Wilf-Miron	2011	157928	cohort	No useable data	Ns and % attendance not reported
Williams	1989	392	RCT	Intervention was confounder	Control-group-only data not calculable for other factors
Yarnall	1993	unclear	case-control	No useable factor	Reports uptake by health provider characteristic(s)*
Zidar	2015	52541	cross-sectional	No useable data	Reports % non-attendance by age group, but no N per category

*Attendance was measured based on a characteristic or behaviour of the medical provider or facility, not a characteristic of the patient, for example, physician's gender, health centre's use of a special assessment form, or the social deprivation status of the health centre (rather than the patient).

For peer review only

Factors associated with attendance at screening for breast cancer: a systematic review and meta-analysis

Journal:	*BMJ Open*
Manuscript ID	bmjopen-2020-046660.R1
Article Type:	Original research
Date Submitted by the Author:	08-Jun-2021
Complete List of Authors:	Mottram, Rebecca; University of Warwick Warwick Medical School, Division of Health Sciences<br>Knerr, Wendy; University of Warwick Warwick Medical School, Division of Health Sciences<br>Gallacher, Daniel; University of Warwick Warwick Medical School, Division of Health Sciences<br>Fraser, Hannah; University of Warwick, Warwick Medical School<br>Al-Khudairy, Lena; University of Warwick Warwick Medical School, Division of Health Sciences<br>Ayorinde, Abimbola; University of Warwick Warwick Medical School, Division of Health Sciences<br>Williamson, Sian; University of Warwick Warwick Medical School, Division of Health Sciences<br>Nduka, Chidozie; University of Warwick Warwick Medical School, Division of Health Sciences<br>Uthman, Olalekan; University of Warwick Warwick Medical School, Division of Health Sciences<br>Johnson, Samantha ; University of Warwick, University of Warwick Library<br>Tsertsvadze, Alexander; University of Ottawa Faculty of Medicine, School of Epidemiology and Public Health<br>Stinton, Christopher; University of Warwick Warwick Medical School, Division of Health Sciences<br>Taylor-Phillips, Sian; University of Warwick Warwick Medical School, Division of Health Sciences<br>Clarke, Aileen; University of Warwick Warwick Medical School, Division of Health Sciences
**Primary Subject Heading**:	Diagnostics
Secondary Subject Heading:	Oncology
Keywords:	Breast tumours < ONCOLOGY, Breast imaging < RADIOLOGY & IMAGING, Diagnostic radiology < RADIOLOGY & IMAGING, PUBLIC HEALTH

I, the Submitting Author has the right to grant and does grant on behalf of all authors of the Work (as defined in the below author licence), an exclusive licence and/or a non-exclusive licence for contributions from authors who are: i) UK Crown employees; ii) where BMJ has agreed a CC-BY licence shall apply, and/or iii) in accordance with the terms applicable for US Federal Government officers or employees acting as part of their official duties; on a worldwide, perpetual, irrevocable, royalty-free basis to BMJ Publishing Group Ltd ("BMJ") its licensees and where the relevant Journal is co-owned by BMJ to the co-owners of the Journal, to publish the Work in this journal and any other BMJ products and to exploit all rights, as set out in our licence.

The Submitting Author accepts and understands that any supply made under these terms is made by BMJ to the Submitting Author unless you are acting as an employee on behalf of your employer or a postgraduate student of an affiliated institution which is paying any applicable article publishing charge ("APC") for Open Access articles. Where the Submitting Author wishes to make the Work available on an Open Access basis (and intends to pay the relevant APC), the terms of reuse of such Open Access shall be governed by a Creative Commons licence – details of these licences and which Creative Commons licence will apply to this Work are set out in our licence referred to above.

Other than as permitted in any relevant BMJ Author's Self Archiving Policies, I confirm this Work has not been accepted for publication elsewhere, is not being considered for publication elsewhere and does not duplicate material already published. I confirm all authors consent to publication of this Work and authorise the granting of this licence.

For peer review only

Factors associated with attendance at screening for breast cancer: a systematic review and meta-analysis

Rebecca Mottram[1] (crosbyr4@gmail.com), Wendy Knerr[1] (wendyknerr@gmail.com), Daniel Gallacher[1] (D.Gallacher@warwick.ac.uk), Hannah Fraser[1] (H.Fraser@warwick.ac.uk), Lena Al-Khudairy[1] (Lena.al-Khudairy@warwick.ac.uk), Abimbola Ayorinde[1] (A.Ayorinde.1@warwick.ac.uk), Sian Williamson[1] (S.Z.Williamson@soton.ac.uk), Chidozie Nduka[1] (C.Nduka@warwick.ac.uk), Olalekan A. Uthman[1] (olalekan.uthman@warwick.ac.uk), Samantha Johnson[2] (Samantha.A.Johnson@warwick.ac.uk), Alexander Tsertsvadze[1 3] (a_tsertsvadze@hotmail.com), Chris Stinton[1] (C.Stinton@warwick.ac.uk), Sian Taylor-Phillips[1] (S.Taylor-Phillips@warwick.ac.uk), Aileen Clarke[1]*(Aileen.Clarke@warwick.ac.uk)

[1]Division of Health Sciences, University of Warwick Medical School, Gibbet Hill Campus, Coventry, CV4 7AL, UK.

[2]University of Warwick Library, University of Warwick, Coventry CV4 7AL, United Kingdom

[3]School of Epidemiology and Public Health, Faculty of Medicine, University of Ottawa, Ottawa, Ontario K1G 5Z3, Canada

*Corresponding author: Aileen Clarke, Warwick Medical School, University of Warwick, Coventry, CV4 7AL, 024761 50189, email:aileen.clarke@warwick.ac.uk

Word count: 3779

Keywords: Breast cancer; mammography; mass screening; systematic review

For peer review only

ABSTRACT

Objective Attendance at population-based breast cancer (mammographic) screening varies. This comprehensive systematic review and meta-analysis assesses all identified patient-level factors associated with routine population breast screening attendance.

Design CINAHL, Cochrane Library, Embase, Medline, OVID, PsycINFO and Web of Science were searched for studies of any design, published January 1987–June 2019, and reporting attendance in relation to at least one patient-level factor.

Data synthesis Independent reviewers performed screening, data extraction and quality appraisal. Odds ratios (OR) and 95% confidence intervals (CIs) were calculated for attendance for each factor and random-effects meta-analysis was undertaken where possible.

Results Of 19,776 studies, 331 were assessed at full text and 66 studies (n=22,150,922) were included. Risk of bias was generally low. In meta-analysis increased attendance was associated with higher socioeconomic status (SES) (n = 11 studies; OR 1.45, 95% CI: 1.20–1.75); higher income (n = 5 studies; OR 1.96, 95% CI: 1.68–2.29); home ownership (n = 3 studies; OR 2.16, 95% CI: 2.08–2.23); being non-immigrant (n = 7 studies; OR 2.23, 95% CI: 2.00–2.48); being married/cohabiting (n = 7 studies; OR 1.86, 95% CI: 1.58–2.19); and medium (versus low) level of education (n = 6 studies; OR 1.24, 95% CI: 1.09–1.41). Women with previous false-positive results were less likely to re-attend (n = 6 studies; OR 0.77, 95% CI: 0.68–0.88). There were no differences by age group or by rural versus urban residence.

Conclusions Attendance was lower in women with lower SES, those who were immigrants, non-homeowners and those with previous false-positive results. Variations in service delivery, screening programmes and study populations may influence findings. Our findings are of univariable associations. Underlying causes of lower uptake such as practical, physical, psychological or financial barriers should be investigated. (CRD42016051597)

Ethics approval statement No ethics approval was required for this type of study.

For peer review only - http://bmjopen.bmj.com/site/about/guidelines.xhtml

ARTICLE SUMMARY

Strengths and limitations of this study

- Comprehensive systematic review of all identified patient-level factors associated with attendance at routine population-based breast cancer (mammographic) screening.
- Two reviewers independently conducted all study selection, data extraction and quality appraisal using QUIPS.
- Both observational and experimental designs were included, using control arms of quasi-experimental or randomized designs, and odds ratios were independently recalculated using each study's raw data.
- Limitations: heterogeneity is high partly due to the large size of studies. Studies were separately meta-analysed by study design, and sensitivity analysis was conducted for one study with an extreme effect size.
- Reporting of potential confounders and effect modifiers was highly variable in studies; this was partially mitigated by re-categorizing variables, such as education levels, to harmonize variables across studies where possible.

For peer review only

For peer review only - http://bmjopen.bmj.com/site/about/guidelines.xhtml

INTRODUCTION

Breast cancer was the most commonly diagnosed cancer worldwide in 2020, with 2.3 million cases, and the most common cause of cancer death in women.[1] Breast cancer incidence is higher in more developed countries (Europe, Australia, New Zealand and North America; 55.9 cases per 100,000 population) than in less developed countries (29.7 per 100,000), while the reverse is true of death rates (12.4 vs 15.0 per 100,000, respectively).[1] In the EU, mortality rates decreased 18.7% between the period 2005–2009 to 2019 from 16.44 to (predicted) 13.36 per 100,000.[2]

Population-based mammographic screening aims to reduce breast cancer mortality. However, there has been controversy about the balance of benefits and harms of breast screening[3] and breast screening programmes have become more aware of the need for promoting informed choice.[4, 5]

Attendance at breast screening is not uniform among the eligible population.[6] Ross et al[7] described attendance at screening as an individual decision (behavioural) which is affected by accessibility of services (structural) and by a woman's immediate surroundings (societal). Characteristics that have been associated with screening attendance can be grouped into a number of categories related to sociodemographic factors; health status; health behaviours; accessibility and logistics; beliefs, attitudes and knowledge; simple intention to attend; and societal factors including health systems financing and organization.[8-11]

Most reviews of factors associated with breast screening attendance have focused on individual factors.[e.g. 12, 13, 14] We aimed to provide a comprehensive systematic review of all identified patient-level characteristics associated with the uptake of population-based mammographic screening, to inform screening programmes of the available evidence about who does and does not attend.

METHODS

Protocol and registration

For peer review only - http://bmjopen.bmj.com/site/about/guidelines.xhtml

The review was conducted in accordance with pre-specified methods documented in the protocol registered on the 22nd of November 2016 in the PROSPERO International Prospective Register of Systematic Reviews database (Supplementary file A).[15]

Search and Information sources

The Cumulative Index to Nursing and Allied Health Literature (CINAHL), Cochrane Library, Embase, Medline, PsycINFO and Web of Science were searched for studies published between the 1st of January 1987 and 26th June 2019. The search was developed in Medline using a combination of MeSH headings and free text terms and adapted for use in the other databases (the search strategy is available in Supplementary file B).

Reference lists of relevant reviews were searched for potentially relevant studies. Experienced researchers with prior studies in the field were contacted to identify other potentially relevant studies that had not been identified in the searches.

Eligibility criteria

Primary studies of any design were included if they reported attendance data from routine population-based mammography screening programmes in relation to at least one patient-level factor, and were written in English between January 1987 and June 2019. Studies were excluded if they involved self-reported mammography uptake, opportunistic screening programmes, data for only a subgroup of the eligible population (e.g. only women in a narrow age range, only immigrants or only rural women), or uptake data by number of invitations sent rather than number of women. Reviews, commentaries, opinions, letters, and non-empirical and qualitative studies were excluded.

Study selection and data extraction process

Pairs of reviewers screened titles and abstracts independently to identify potentially relevant studies with third reviewer cross-check. Two reviewers independently assessed full-text studies for formal inclusion/exclusion assessment against pre-defined eligibility criteria with third reviewer cross-check. Disagreements were resolved by a consensus between the two reviewers or by help of a third reviewer.

Data from included studies were extracted and then crosschecked by two reviewers independently. The data included the number of women who attended mammographic

For peer review only - http://bmjopen.bmj.com/site/about/guidelines.xhtml

screening and the number invited, and data on patient characteristics, including: sociodemographic factors, such as age, marital status, educational level, race/ethnicity, immigration status and socioeconomic status (SES, which was measured in two ways, a) with various composite indices of deprivation that included factors such as housing density, employment, education, social support, car ownership, and crime prevalence, and b) based on household income); beliefs, attitudes and socioemotional factors; health history and behaviours; logistic and accessibility factors (e.g. distance from screening centre).

Risk of bias of included studies

Risk of bias (RoB) of all included studies was appraised by two independent reviewers using the Quality in Prognosis Studies (QUIPS) tool.[16] The QUIPS tool covers six RoB domains (participation, attrition, prognostic factor, confounding factors, outcome measurement and analysis and reporting), each of which includes multiple items that are judged separately. A conclusive judgment for each RoB domain is reached and expressed on a three-grade scale (high, moderate or low RoB).

Synthesis of data

We used raw attendance data to calculate unadjusted odds ratios for each factor. A random effects model-based meta-analysis was conducted for an association between a factor of interest (dichotomous or more categories) and the dichotomous outcome (screening attendance) to generate Mantel-Haenszel odds ratios with 95% confidence intervals, when possible.[17] Random effects models were used to allow for heterogeneity in the effects of the factors considered to vary across the different studies.

In addition to the main meta-analyses, we conducted separate meta-analyses for a) observational studies whose samples were made up only of women who had previously attended screening (hereafter referred to as rescreening studies) and b) intervention studies (quasi-experimental and randomized controlled trials) that reported characteristics separately for intervention and control arms, recording only data for the control group, as their attendance would not be influenced by exposure to an intervention. We also conducted a sensitivity analysis to determine the impact of a study with an extreme effect size[18] on the meta-analysis of SES.

For peer review only - http://bmjopen.bmj.com/site/about/guidelines.xhtml

For peer review only

We summarized results narratively if there were inadequate quantitative data for meta-analysis, if variables were reported in fewer than three studies,[17] or if the data from multiple studies were highly variable and therefore could not be meaningfully pooled.

This review is reported according to PRISMA guidelines (Supplementary file C).[19] All analyses were conducted in Stata v16.

Patient and public involvement

Public contributors were involved in design and informed of ongoing progress and findings as part of the West Midlands Centres for Leadership in Applied Health Research. Results were reported back to the contributors as part of the wider dissemination activities of the relevant theme in the Centres for Leadership in Applied Health Research.

RESULTS

Literature search

The process of study flow and reasons for exclusion are provided in Figure 1. In brief, the searches of electronic databases identified 11,949 unique publications (after deduplication), published between January 1987 and June 2019, of which 11,618 were excluded at the level of abstract/title screening, leaving 331 records for full text review. Of the 331 full texts, 66 unique studies reported in 67 publications were included.[18, 20-87]

Study characteristics

Characteristics of all included studies are listed in Supplementary file D. Of the 66 studies: 49 were observational (45 retrospective cohort, 2 cross-sectional and 2 case-control designs); and 17 were intervention studies (16 randomized controlled trials and 1 quasi-experimental). Sample sizes ranged from 82 to 4.8 million.

The studies were conducted in Europe (n = 40), North America (n = 18), Asia-Pacific (n = 5) and the Middle East (n = 3). The UK had the most studies (n = 16) followed by the USA (n = 11).

We were able to pool data from 31 observational studies (reported in 32 publications) on the attendance at screening in relation to nine factors (age, education, home ownership, immigration status, marital status, results of previous mammogram, rural/urban residence,

For peer review only - http://bmjopen.bmj.com/site/about/guidelines.xhtml

SES and income) (Table 1). We were only able to pool data from three intervention studies, and only for one factor (age).

Adequate data for meta-analysis was not provided for 35 studies; although six of these studies provided adequate data to calculate odds ratios and confidence intervals, and are narratively reported in Table 2. The remaining 29 studies reported data that could not be analysed. (Reasons are detailed in Supplementary file E). In brief, 14 of the 29 studies were intervention trials, where data were not in the right format for us to use. The other 15 studies could not be analysed because uptake data were reported by health-provider characteristics rather than patient characteristics; because the paper reported percentage uptake but not sample sizes per category; or because data for different factors were not reported separately.

Risk of bias

RoB across studies was generally low on all domains (Figure 2). For study participation, 71% of studies were considered at low RoB; for attrition, 91%; for outcome measurement, 97%; and for statistical analysis and reporting, 83%. For measurement of variables associated with attendance (prognostic factors), more than half (61%) of studies had a low RoB, while 23% had a high RoB, mostly due to SES being measured at the area level (e.g. neighbourhood) rather than at the individual level. More than half of studies (53%) had a low RoB with regard to measuring potential confounders, with around one-quarter (27%) having a moderate risk and just over one-fifth (21%) having a high risk.

Quantitative data analysis (meta-analyses)

Table 1 presents unadjusted odds ratio estimates with their 95% confidence intervals of attendance at breast screening for factors that were reported in three or more studies. The analyses gave I^2 values of around 99%, meaning that there was a high level of heterogeneity, except for the analysis of homeowners versus tenants, where the I^2 value was 38.9% (Table 1).

Table 1. Results of meta-analyses*

Variables	Number of women (number of studies included)**	% uptake	Odds ratio of attendance (unadjusted): range | overall (95% confidence interval)

For peer review only - http://bmjopen.bmj.com/site/about/guidelines.xhtml
For peer review only

**Age** (60—69 vs 50—59) †			
Observational studies	5,065,779 (16)	56% vs 55%	0.65–1.42 | 0.97 (0.88–1.08)
Intervention studies	2343 (3)	52% vs 57%	0.24–1.16 | 0.78 (0.47–1.31)
Rescreening studies (age at initial screen)	271,641 (3)	74% vs 74%	0.93–1.05 | 0.99 (0.93–1.06)
**Education level**	550,646 (6)		
medium vs low		83% vs 77%	1.05–1.45 | 1.24 (1.09–1.41)
high vs low		81% vs 77%	0.76–1.31 | 1.11 (0.97–1.26)
high vs medium		81% vs 83%	0.61–1.10 | 0.89 (0.78–1.02)
**Housing tenure** (homeowner vs tenant/non-owner)	223,293 (3)	84% vs 70%	2.06–2.20 | 2.16 (2.08–2.23)
**Country of origin** (non-immigrants vs immigrants)	2,409,902 (7)	81% vs 60%	1.75–2.81 | 2.23 (2.00–2.48)
**Income**	1,193,238 (5)		
intermediate vs low		77% vs 66%	1.78–2.09 | 1.96 (1.68–2.29)
high vs low		80% vs 66%	1.61_2.87 | 2.18 (1.86–2.56)
high vs intermediate		80% vs 77%	0.81–1.37 | 1.11 (0.95–1.30)
**Marital Status** (married/cohabiting vs unmarried/non-cohabiting)	1,293,753 (7)	80% vs 69%	1.38–2.36 | 1.86 (1.58–2.19)
**Residence** (rural vs urban)	65,641(3)	74% vs 65%	0.80–1.59 | 1.12 (0.76–1.66)
**Previous result of mammogram** (rescreening studies only: false positive vs normal)	3,540,953 (6)	60% vs 68%	0.49–0.89 | 0.78 (0.68–0.88)
**Socioeconomic status (SES)**	6,600,283 (11)		
medium vs low		56% vs 48%	1.08–2.35 | 1.45 (1.20–1.75)
high vs low		54% vs 48%	0.75–3.59 | 1.69 (1.40–2.05)‡
high vs medium		54% vs 56%	0.69–1.53 | 1.17 (0.96–1.41)

**All results in this table are for observational studies except the data for age, which includes results for the separate meta-analysis of intervention studies.*
***References for studies pooled for meta-analyses of observational studies are provided in forest plots in Figures 3 and 4.*
†We focused on the age bands most commonly eligible in population-based programmes and did not analyse odds for those younger than age 50 or older than 69.
‡The odds ratios and confidence intervals for SES include all relevant observational studies. We also performed a sensitivity analysis by removing the large study from France by DeBorde et. al,[18] which found that women with high or medium SES

For peer review only - http://bmjopen.bmj.com/site/about/guidelines.xhtml

were both more likely to attend compared to women of lower SES (OR 1.84, 95% CI: 1.55–2.17, p < 0.001; and OR 1.49, 95% CI: 1.27–1.76, p < 0.001, respectively).

We compared the odds of attending mammographic screening by the age bands most commonly eligible for national screening programmes (60—69 and 50—59). There was no significant difference by age group in meta-analyses of observational studies (n = 16; OR 0.97, 95% CI: 0.88–1.08, p = 0.631, Figure 3) or intervention trials (n = 3; OR 0.78, 95% CI: 0.47–1.31, p = 0.354).

We grouped education data from six studies to approximate the United Nations Educational, Scientific and Cultural Organization (UNESCO) three-level classification: low (≤10 years), middle (11–15 years) and high (>15 years). Compared to women with a low level of education, women with a medium level were more likely to attend (OR 1.24, 95% CI: 1.09–1.41, p < 0.001). Results from comparisons of women with a high level of education versus low or medium levels were not statistically significant (Figure 4A).

The odds of attending mammographic screening were higher for homeowners than for tenants or non-owners (n = 3; OR 2.16, 95%CI: 2.08–2.23, p < 0.001, Figure 3).

Meta-analysis of participants' country of origin showed that people born in the study country (non-immigrants) were more likely to attend than immigrants (n = 7; OR 2.23, 95% CI: 2.00–2.48 , p < 0.001, Figure 3).

We meta-analysed attendance using two measures of SES. Data for overall SES from 11 studies were grouped into low, medium and high categories. Women with medium or high SES were more likely to attend than those with a low SES (medium vs low SES OR 1.45, 95% CI: 1.20–1.75, p < 0.001; high vs low SES OR 1.69, 95% CI: 1.40–2.05, p < 0.001, Figure 4B). One study from France (DeBorde)[18] (n = 4.8 million) reported that women with a higher SES were less likely to attend than those with either a low or intermediate SES. We conducted a sensitivity analysis excluding that study, but it made very little difference to the odds of attending: women with high or medium SES were both more likely to attend compared to women of lower SES (OR 1.84, 95% CI: 1.55–2.17, p < 0.001, and OR 1.49, 95% CI: 1.27–1.76, p < 0.001, respectively).

For peer review only - http://bmjopen.bmj.com/site/about/guidelines.xhtml

Data on income from five studies was grouped into low, intermediate and high categories. Women with an intermediate or high income were more likely to attend than those with low income (intermediate vs low income OR 1.96, 95% CI: 1.68–2.29, $p < 0.001$; high vs low OR 2.18, 95% CI: 1.86–2.56, $p < 0.001$; high vs intermediate OR 1.11, 95% CI: 0.95-1.30, p = 0.20, Figure 4C). For both income and SES there was no significant difference between women at intermediate and high levels, indicating that there was no statistically significant dose response effect for higher SES or income.

Women who were married or cohabiting were more likely to attend than their unmarried or non-cohabiting counterparts (n = 7; OR 1.86, 95% CI: 1.58–2.19 , $p <0.001$, Figure 3).

We analysed data separately for studies with samples made up only of women who had previously attended mammographic screening (i.e. rescreening studies). Six of these studies reported data on attendance based on the results of a previous mammogram. Women who had previously received a false-positive result were less likely to attend than those with a normal result (OR 0.78, 95%CI: 0.68–0.88, $p <0.001$, Figure 3).

There was no statistically significant difference in attendance among women living in rural compared to urban areas (n = 3; OR 1.12, 95% CI: 0.76–1.66, , p = 0.557).

Narrative synthesis

Factors that could not be meta-analysed (because they were reported in fewer than three studies or could not be pooled) are reported in Table 2 with odds ratios.

These studies include a variety of factors associated with reduced attendance clustered around sociodemographic, accessibility and logistics (living in crowded housing and being unemployed, receiving disability benefits, lack of access to a vehicle), and spoken language not English.

Associations with women's health status, behaviours, attitudes and knowledge showed a mixed picture. There was some evidence that good general health, lack of comorbidity and not taking hormone replacement therapy (HRT) were all associated with higher attendance, but studies also reported higher attendance among women with a higher numbers of previous clinic visits, depression and heart disease. A previous negative attitude to breast

For peer review only

screening, limiting long-term illness, schizophrenia, non-work-related stress and current smoking were associated with lower attendance.

Factors that did not show any statistical difference included body mass index (BMI) and service provision factors. No difference in women's attendance was found according to availability of general practitioners or radiologists or physician years since graduation, and there were mixed results according to distance to screening centre and diabetes.

Table 2. Likelihood of attending screening by factors not suitable for meta-analysis in observational studies

Variable	N*	Included studies	% uptake: variable vs reference category	Odds ratio (95% confidence interval)
**Less likely to attend**				
No access to vehicle	144181	Jensen 2012b	61% vs 82%	0.33 (0.32–0.34)
	37059	O'Reilly 2012	60% vs 78%	0.43 (0.41–0.46)
Negative attitude about breast screening	497	Kee 1993	53% vs 60%	0.44 (0.35–0.55)
Receiving disability benefits	885979	Le 2019	69% vs 76%	0.70 (0.70–0.71)
First invitation to screening	742786	Renshaw 2010	40% vs 76%	0.22 (0.21–0.22)
Spoken/preferred language not English	18851	Blanchard 2004	62% vs 83%	0.33 (0.28–0.39)
	43819	Tatla 2003	60% vs 78%	0.43 (0.41–0.46)
Long-term limiting illness	37059	O'Reilly 2012	71% vs 77%	0.71 (0.68–0.75)
	144264	Jensen 2015b	71% vs 80%	0.64 (0.61–0.66)
Smoking (current)	28874	Katz 2018	84% vs 88%	0.72 (0.65–0.79)
Living in crowded housing conditions	31948	Zackrisson 2004	37% vs 66%	0.29 (0.24–0.36)
Employment status				
Outside workforce vs employed/self-employed	640843	Le 2019	63% vs 77%	0.51 (0.50–0.51)
	119269	Jensen 2012b	77% vs 83%	0.66 (0.64–0.68)
Unemployed vs employed/self-employed	481911	Le 2019	61% vs 77%	0.47 (0.45–0.49)
	102178	Jensen 2012b	67% vs 83%	0.41 (0.40–0.43)

For peer review only - http://bmjopen.bmj.com/site/about/guidelines.xhtml

For peer review only

Number of childbirths	46041	Lagerlund 2002		
0 vs 1–2			82% vs 91%	0.44 (0.40–0.48)
3+ vs 1–2			90% vs 91%	0.81 (0.75–0.87)
No family history of BC	119502	O'Byrne 2000	85% vs 86%	0.90 (0.86–0.94)
Type of clinic (mobile vs fixed)	119502	O'Byrne 2000	84% vs 85%	0.93 (0.88–0.98)
Schizophrenia	110240	Chochinov 2009	45% vs 58%	0.58 (0.52–0.64)
**More likely to attend**				
No comorbidities	76520	Larsen 2018	82% vs 75%	1.53 (1.46–1.60)
60+ primary care visits during 6-year study period (vs <60)	43968	Katz 2018	91% vs 79%	2.70 (2.55–2.86)
Depression	38823	Katz 2018	86% vs 85%	1.12 (1.02–1.23)
Good general health	37059	O'Reilly 2012	77% vs 68%	1.55 (1.46–1.64)
Heart disease	6501	Katz 2018	90% vs 85%	1.75 (1.61–1.91)
Not living in capital city	885979	Le 2019	76% vs 62%	1.94 (1.91–1.97)
Previous attender	11664	Taylor-Phillips 2013	73% vs 45%	3.32 (3.05–3.61)
Citizen of country	885979	Le 2019	75% vs 51%	2.88 (2.82–2.94)
Member of majority racial/ethnic group	17997	Blanchard 2004	85% vs 75%	1.70 (1.52–1.89)
Religion				
Catholic vs none	37140	O'Reilly 2012	74% vs 68%	1.40 (1.25–1.47)
Protestant vs none		O'Reilly 2012	77% vs 68%	1.57 (1.46–1.70)
Never HRT use	119502	O'Byrne 2000	16% vs 14%	1.13 (1.09–1.17)
Referral by health professional	56420	Tatla 2003	77% vs 76%	1.05 (1.00–1.10)
**No difference in attendance or mixed results**				
BMI	19168	Katz 2018	87% vs 87%	0.95 (0.87–1.04)
>0 GPs per 100,000 inhabitants	4865	Pornet 2010	55% vs 56%	0.96 (0.85–1.08)
>0 radiologists per 100,000 inhabitants	4865	Pornet 2010	52% vs 56%	0.87 (0.72–1.05)
Diabetes	9849	Katz 2018	87% vs 84%	1.25 (1.17–1.33)

For peer review only

	504288	Chan 2014	60% vs 66%	0.79 (0.78–0.80)
Distance to screening centre	137419	Jensen 2012b	77% vs 80%	0.86 (0.84–0.88)
	833856	St-Jacques 2013	53% vs 52%	1.02 (1.01–1.03)
	13260	Ouédraogo 2014	54% vs 50%	0.85 (0.79–0.91)
Physician years since graduation	105575	Makedonov 2015	74% vs 75%	1.03 (0.99–1.06)

**Reflects the number of participants analysed for each factor, which can differ for different factors in the same study depending on data availability.*

DISCUSSION

We undertook a comprehensive review of the current evidence on patient-level factors associated with breast cancer (mammographic) screening attendance. Where appropriate, meta-analyses were performed to determine the strength of association.

Main findings

In line with other systematic reviews, we found that in general higher SES status, higher income [14], being born in the country of residence (i.e. non-immigrant),[12] and home ownership (compared to renting) predicted mammographic screening attendance. However, it appears that women with a higher SES or income were not more likely to attend than those with an intermediate level. We hypothesize that women with a higher SES may be more likely to use alternative screening services (i.e. opportunistic or privately funded screening) compared to women with a low or intermediate SES, thus their attendance would not be apparent in studies using data from national screening programmes. This was suggested as a limitation by many of the included studies in this review, most notably the large study from France[18] (n = 4.8 million), which was the only study to find that women with a higher SES were less likely to attend than those with either a low or intermediate SES. The authors of that study note the high levels of opportunistic screening available to women with a high SES in France. We conducted a sensitivity analysis excluding that study, but it made very little difference to the odds ratios for attendance.

A medium level of education was also associated with screening attendance when compared to a low level, but a higher level of education was not associated with increased attendance compared to either medium or lower levels. As with the analyses of SES, it is

For peer review only - http://bmjopen.bmj.com/site/about/guidelines.xhtml

possible that women with the highest levels of education are more likely to use alternative screening services not reflected in data from public screening programmes.

We hypothesized that some variation in relation to education or SES might be due to changes in women's attitudes to breast screening as a result of concerns about its overall benefits,[65, 88] perhaps related to the informed-choice agenda.[4] However, we found no population screening studies investigating this.

Our results also support previous research indicating that marital status is associated with attendance at mammography,[65, 88-91] with women who were married or cohabiting more likely to attend than their unmarried or non-cohabiting counterparts. Previous literature indicates lower uptake among women from minority-ethnic backgrounds.[92, 93] While our data were not sufficient to meta-analyse ethnicity, we did find that immigrant women were less likely to attend screening than non-immigrants.

We did not find a significant effect of age. There was very high heterogeneity here, with individual large studies finding highly statistically significant results in both directions. We hypothesized that attendance may be higher among older women because they have been invited to breast screening for at least two decades, and attendance may have become more routine in this cohort, and possibly less likely to be affected by recent debates around the risks and benefits of screening. To explore this, we did a post-hoc analysis of the effect of age on attendance by the year of study completion. We found that older women were more likely to attend compared to younger women in more recent studies (i.e. those completed since 2010), but that the opposite was true in older studies, particularly those published before 2005.

Women who received a false-positive result at a previous screening were less likely to attend than those with a normal result, confirming previous findings.[94]

Strengths and limitations

This review has many strengths. The large number of studies included (n = 66), involving more than 22 million women, represents a comprehensive overview of available evidence. Studies included in the meta-analysis were judged to have a low risk of bias on most domains and included large numbers of women. At least two reviewers were involved at all

For peer review only - http://bmjopen.bmj.com/site/about/guidelines.xhtml

stages to reduce the risk of errors and bias. This study was undertaken from the perspective of population-based breast cancer screening programmes and we were strict in our eligibility criteria in including only those studies. Studies where the sampling frame was restricted to population subgroups (and not based on population-based screening programmes) were excluded. We also excluded studies that relied on self-reported attendance (though it is important to note that self-report is essential for some factors, such as ethnicity and attitudes to screening).

A limitation is that most studies reported cross-sectional attendance data, which included mixed groups of those who were attending for the first time and some who had previously attended. Also, we inevitably had to make choices of categories for meta-analysis which may affect meta-analytic results; where possible we used independent sources to select appropriate categorizations.

The main limitation of this review is significant between-study heterogeneity. Although we used random effect models throughout, our results should be considered in light of this. We chose random effects models as almost all of our analyses contained heterogeneity and it is also expected that there would be differences in attendance across the different study populations. Studies with larger sample sizes are assumed to contain the least uncertainty and are given higher weightings than smaller studies. For analyses of small numbers of studies, the random effects analysis may struggle to correctly estimate uncertainty, but any meta-analysis performed on few studies would have its limitations, and the use of random effects analysis maintained consistency with the other analyses.

Heterogeneity may in part be due to differences between health systems and the organization of mammographic screening, as well as differences in the culture and attitudes of the populations served. We conducted sensitivity analysis to determine the impact of a very large study with an extreme effect size[18] on the meta-analysis of SES. For some outcomes (such as age) the heterogeneity encompasses studies with highly significant results in both directions, and here the results of the meta-analysis should be interpreted with great caution. For other variables (such as re-attendance after false-positive results) the high I^2 simply reflects that there were very large studies with very small confidence intervals, which all had point estimates of different magnitude in the same direction. Here

For peer review only - http://bmjopen.bmj.com/site/about/guidelines.xhtml

the meta-analysis results show a consistent effect, with some disagreement between studies on the exact size of effect.

Another limitation is that we extracted univariable associations with uptake. In practice, many of the variables investigated will be highly correlated, and there will be complex interactions and confounding which we have not been able to account for. While some studies did report multivariable models, these were varied in structure, methods and variables included, so would have been difficult to combine in any meaningful way. We were therefore unable to undertake multivariable meta-regression analysis, examining the effects of individual attendance factors on overall attendance.

For the studies included in the narrative analysis, large numbers of women were also often involved, but these studies should be treated with caution as they are potentially subject to bias. The risk of confounding was found to be high in these studies using the QUIPs tool. However, confounding is inherent in the design of population-based observational and especially ecological designs.

To investigate the risk of reporting bias, we conducted funnel plots (Supplementary file F), which demonstrated the high level of heterogeneity present between the studies in our analyses. Age was the only analysis where the studies disagree over the direction of attendance, however the disagreement is among larger studies, suggesting this is unlikely to be associated with biased reporting and instead down to the study heterogeneity. All other analyses, while having studies which disagree on the point estimate, have agreement as to which group is more or less likely to attend mammographic screening. Overall, we are not concerned about reporting bias.

Finally, we have not included health insurance (or lack of health insurance) as a factor in the narrative analysis because of the problems of comparison between countries.

CONCLUSIONS

A wide variety of factors affect a woman’s decision to attend breast screening. Our main findings are that attendance was lower in women with lower SES, those who were immigrants, non-homeowners and those with previous false-positive results. Based on our current findings, if screening programmes wish to improve equity of access to breast

For peer review only - http://bmjopen.bmj.com/site/about/guidelines.xhtml

screening services they should concentrate on women facing access (practical, physical, psychological and financial) barriers.

Future research in this area would also need to systematically assess the effects of interventions to reduce the impact of access barriers to screening attendance.

DEVIATIONS FROM STUDY PROTOCOL

To assess RoB, the QUIPS tool was used rather than the Quality Assessment Tool; and for data synthesis, despite significant heterogeneity, meta-analysis was possible for some predictors. In addition, we clarified our inclusion criteria to include only studies with data from routine population-based mammography screening programmes in order to ensure generalizability.

AUTHOR CONTRIBUTIONS

RM conceived the study as part of her PhD Dissertation, and it was further refined in collaboration with AC and STP. AC, CS and WK further developed the inclusion and exclusion criteria. SJ undertook database searches and AC, HF, RM, LAK, SW and WK reviewed titles and abstracts. Each study retained for full-text review was reviewed by RM and WK. Discrepancies regarding inclusion and exclusion were resolved by AC and CS. RM and WK did data extraction, and data were checked by OU and CN. Studies were critically appraised by AA, AT, CS and WK. Meta-analyses were conducted by DG. Thematic synthesis was done by AC, STP and WK. All authors contributed to the manuscript and approved the final version.

ACKNOWLEDGEMENTS

The authors would like to thank Magdalena Skrybant, Patient and Public Involvement and Engagement Lead, Applied Research Centre West Midlands (formerly Centres for Leadership in Applied Health and Care, West Midlands) for her support in coordinating public engagement in this project.

FUNDING

This research was funded by the National Institute for Health Research Collaboration for Leadership in Applied Health Research and Care West Midlands (NIHR CLAHRC WM), now

For peer review only - http://bmjopen.bmj.com/site/about/guidelines.xhtml

recommissioned as NIHR Applied Research Collaboration West Midlands (NIHR ARC WM). Rebecca Mottram, Lena Al-Khudairy, Sian Taylor-Phillips, Sian Williamson, Hannah Fraser, Chris Stinton, and Aileen Clarke were all supported by the NIHR CLAHRC WM and Wendy Knerr, Abimbola Ayorinde, Aileen Clarke and Lena Al-Khudairy are all partly supported by the NIHR ARC WM.

Olalekan Uthman is supported by the NIHR using Official Development Assistance (ODA) funding. Sian Taylor-Phillips is supported by an NIHR Career Development Fellowship (CDF-2016-09-018). The views expressed in this publication are those of the author(s) and not necessarily those of the UK National Health Service (NHS), the NIHR or the Department of Health and Social Care.

COMPETING INTERESTS

Dr. Mottram reports personal fees from the National Institute for Health Research (NIHR) Centre for Leadership in Applied Research and Health Care (CLARHC) West Midlands during the conduct of the study. Dr. Al-Khudairy reports grants from the NIHR during the conduct of the study. Dr. Taylor-Phillips reports grants from NIHR outside the submitted work. Dr. Clarke reports grants from the NIHR for the NIHR Applied Research Centre (ARC) West Midlands and previously from the NIHR CLARHC West Midlands, which supported her and researchers working on this project. Dr. Clarke also received grants from Public Health England (PHE) outside the submitted work. All other authors report no competing interests.

DATA SHARING STATEMENT

No new data have been created in the preparation of this report and therefore there is nothing available for access and further sharing. All queries should be submitted to the corresponding author.

REFERENCES

1. Sung H, Ferlay J, Siegel RL, et al. Global cancer statistics 2020: GLOBOCAN estimates of incidence and mortality worldwide for 36 cancers in 185 countries. *CA Cancer J Clin*.n/a(n/a).
2. Malvezzi M, Carioli G, Bertuccio P, et al. European cancer mortality predictions for the year 2019 with focus on breast cancer. *Ann Oncol*. 2019;30(5):781-7.
3. Marmot MG, Altman DG, Cameron DA, et al. The benefits and harms of breast cancer screening: an independent review. *Br J Cancer*. 2013;108(11):2205-40.

For peer review only - http://bmjopen.bmj.com/site/about/guidelines.xhtml

4. Martínez-Alonso M, Carles-Lavila M, Pérez-Lacasta MJ, et al. Assessment of the effects of decision aids about breast cancer screening: a systematic review and meta-analysis. *BMJ Open*. 2017;7(10):e016894.
5. Reder M, Berens E-M, Spallek J, et al. Development of the Informed Choice in Mammography Screening Questionnaire (IMQ): factor structure, reliability, and validity. *BMC Psychology*. 2019;7(1):17.
6. Coyle C, Kinnear H, Rosato M, et al. Do women who intermittently attend breast screening differ from those who attend every invitation and those who never attend? *J Med Screen*. 2012;19(2):98-103.
7. Ross NA, Rosenberg MW, Pross DC, et al. Contradictions in women's health care provision: A case study of attendance for breast cancer screening. *Soc Sci Med*. 1994;39(8):1015-25.
8. Aarts MJ, Voogd AC, Duijm LEM, et al. Socioeconomic inequalities in attending the mass screening for breast cancer in the south of the Netherlands-associations with stage at diagnosis and survival. *Breast Cancer Res Treat*. 2011;128(2):517-25.
9. Ackerson K, Preston SD. A decision theory perspective on why women do or do not decide to have cancer screening: systematic review. *J Adv Nurs*. 2009;65(6):1130-40.
10. Crosby R. Predictors of uptake of screening mammography. Coventry: University of Warwick; 2018.
11. Schueler KM, Chu PW, Smith-Bindman R. Factors associated with mammography utilization: a systematic quantitative review of the literature. *J Womens Health (Larchmt)*. 2008;17(9):1477-98.
12. Bhargava S, Moen K, Qureshi SA, et al. Mammographic screening attendance among immigrant and minority women: a systematic review and meta-analysis. *Acta Radiol*. 2018;59(11):1285-91.
13. Mathioudakis AG, Salakari M, Pylkkanen L, et al. Systematic review on women's values and preferences concerning breast cancer screening and diagnostic services. *Psychooncology*. 2019;28(5):939-47.
14. Smith D, Thomson K, Bambra C, et al. The breast cancer paradox: A systematic review of the association between area-level deprivation and breast cancer screening uptake in Europe. *Cancer Epidemiol*. 2019;60:77-85.
15. Crosby R, Williamson S, Stinton C, et al. A systematic review to identify the worldwide predictors of breast screening uptake PROSPERO International prospective register of systematic reviews: University of York; 2016 [Available from: http://www.crd.york.ac.uk/PROSPERO/display_record.asp?ID=CRD42016051597.
16. Hayden JA, van der Windt DA, Cartwright JL, et al. Assessing bias in studies of prognostic factors. *Ann Intern Med*. 2013;158(4):280-6.
17. Valentine J, Pigott T, Rothstein H. How many studies do you need? A primer on statistical power for meta-analysis. *Journal of Educational and Behavioural Statistics*. 2010;35(2):215-47.
18. Deborde T, Chatignoux E, Quintin C, et al. Breast cancer screening programme participation and socioeconomic deprivation in France. *Prev Med*. 2018;115:53-60.
19. Liberati A, Altman DG, Tetzlaff J, et al. The PRISMA Statement for Reporting Systematic Reviews and Meta-Analyses of Studies That Evaluate Health Care Interventions: Explanation and Elaboration. *PLoS Med*. 2009;6(7):e1000100.
20. Allgood PC, Maxwell AJ, Hudson S, et al. A randomised trial of the effect of postal reminders on attendance for breast screening. *Br J Cancer*. 2016;114(2):171-6.
21. Andersen SB, Vejborg I, von Euler-Chelpin M. Participation behaviour following a false positive test in the Copenhagen mammography screening programme. *Acta Oncol*. 2008;47(4):550-5.
22. Barlow WE, Beaber EF, Geller BM, et al. Evaluating Screening Participation, Follow-up, and Outcomes for Breast, Cervical, and Colorectal Cancer in the PROSPR Consortium. *J Natl Cancer Inst*. 2020;112(3):238-46.

For peer review only - http://bmjopen.bmj.com/site/about/guidelines.xhtml

23. Berens EM, Stahl L, Yilmaz-Aslan Y, et al. Participation in breast cancer screening among women of Turkish origin in Germany - a register-based study. *BMC Womens Health*. 2014;14:24.
24. Blanchard K, Colbert JA, Puri D, et al. Mammographic screening: patterns of use and estimated impact on breast carcinoma survival. *Cancer*. 2004;101(3):495-507.
25. Bourmaud A, Soler-Michel P, Oriol M, et al. Decision aid on breast cancer screening reduces attendance rate: results of a large-scale, randomized, controlled study by the DECIDEO group. *Oncotarget*. 2016;7(11):12885-92.
26. Chiarelli AM, Mai V, Moravan V, et al. False-Positive Result and Reattendance in the Ontario Breast Screening Program. *J Med Screen*. 2003;10(3):129-33.
27. Douglas E, Waller J, Duffy SW, et al. Socioeconomic inequalities in breast and cervical screening coverage in England: are we closing the gap? *J Med Screen*. 2016;23(2):98-103.
28. Finney Rutten LJ, Ebbert JO, Jacobson DJ, et al. Changes in U.S. Preventive Services Task Force recommendations: effect on mammography screening in Olmsted County, MN 2004-2013. *Prev Med*. 2014;69:235-8.
29. Gatrell A, Garnett S, Rigby J, et al. Uptake of screening for breast cancer in South Lancashire. *Public Health*. 1998;112(5):297-301.
30. Goldzahl L, Hollard G, Jusot F. Increasing breast-cancer screening uptake: A randomized controlled experiment. *J Health Econ*. 2018;58:228-52.
31. Hyndman JC, Holman CD, Dawes VP. Effect of distance and social disadvantage on the response to invitations to attend mammography screening. *J Med Screen*. 2000;7(3):141-5.
32. Jensen LF, Pedersen AF, Andersen B, et al. Identifying specific non-attending groups in breast cancer screening--population-based registry study of participation and socio-demography. *BMC Cancer*. 2012b;12:518.
33. Jensen LF, Pedersen AF, Andersen B, et al. Non-participation in breast cancer screening for women with chronic diseases and multimorbidity: a population-based cohort study. *BMC Cancer*. 2015b;15:798.
34. Katz D, Tengekyon AJ, Kahan NR, et al. Patient and physician characteristics affect adherence to screening mammography: A population-based cohort study. *PLoS One*. 2018;13(3).
35. Kee F, Telford AM, Donaghy P, et al. Enhancing mammography uptake: who do women listen to? *Eur J Cancer Prev*. 1993;2(1):37-42.
36. Lagerlund M, Maxwell AE, Bastani R, et al. Sociodemographic predictors of non-attendance at invitational mammography screening--a population-based register study (Sweden). *Cancer Causes Control*. 2002;13(1):73-82.
37. Lagerlund M, Merlo J, Vicente RP, et al. Does the Neighborhood Area of Residence Influence Non-Attendance in an Urban Mammography Screening Program? A Multilevel Study in a Swedish City. *PLoS One*. 2015;10(10):e0140244.
38. Lagerlund M, Sontrop JM, Zackrisson S. Psychosocial factors and attendance at a population-based mammography screening program in a cohort of Swedish women. BMC Womens Health [Internet]. 2014 2014; 14(1):[33 p.]. Available from: http://europepmc.org/abstract/MED/24565263.
39. Larsen SH, Virgilsen LF, Kristiansen BK, et al. Strong association between cervical and breast cancer screening behaviour among Danish women; A register-based cohort study. *Preventive Medicine Reports*. 2018;12:349-54.
40. Le M, Hofvind S, Tsuruda K, et al. Lower attendance rates in BreastScreen Norway among immigrants across all levels of socio-demographic factors: a population-based study. *Journal of Public Health (Germany)*. 2019;27(2):229-40.
41. Leung J, Macleod C, McLaughlin D, et al. Screening mammography uptake within Australia and Scotland in rural and urban populations. *Prev Med Rep*. 2015;2:559-62.
42. Lim SM, Lee HY, Choi KS, et al. Trends of mammography use in a national breast cancer screening program, 2004-2008. *Cancer Res Treat*. 2010;42(4):199-202.
43. Luckmann R, Costanza ME, White MJ, et al. A 4-year randomized trial comparing three outreach interventions to promote screening mammograms. *Transl Behav Med*. 2019;9(2):328-35.

For peer review only - http://bmjopen.bmj.com/site/about/guidelines.xhtml

44. Makedonov I, Gu S, Paszat LF. Organized breast screening improves reattendance compared to physician referral: A case control study. *BMC Cancer*. 2015;15 (1) (no pagination)(315).
45. Matson S, Andersson I, Berglund G, et al. Nonattendance in mammographic screening: a study of intraurban differences in Malmo, Sweden, 1990-1994. *Cancer Detect Prev*. 2001;25(2):132-7.
46. Maxwell AJ, Beattie C, Lavelle J, et al. The effect of false positive breast screening examinations on subsequent attendance: retrospective cohort study. *J Med Screen*. 2013;20(2):91-8.
47. Mayer JA, Lewis EC, Slymen DJ, et al. Patient reminder letters to promote annual mammograms: a randomized controlled trial. *Prev Med*. 2000;31(4):315-22.
48. McCann J, Stockton D, Godward S. Impact of false-positive mammography on subsequent screening attendance and risk of cancer. *Breast Cancer Res*. 2002;4 (5) (no pagination)(R11).
49. Meldrum P, Turnbull D, Dobson HM, et al. Tailored written invitations for second round breast cancer screening: a randomised controlled trial. *J Med Screen*. 1994;1(4):245-8.
50. Merrick EL, Hodgkin D, Horgan CM, et al. Testing novel patient financial incentives to increase breast cancer screening. *Am J Manag Care*. 2015;21(11):771-9.
51. Moss SM, Brown J, Garvican L, et al. Routine breast screening for women aged 65-69: results from evaluation of the demonstration sites. *Br J Cancer*. 2001;85(9):1289-94.
52. O'Byrne AM, Kavanagh AM, Ugoni A, et al. Predictors of non-attendance for second round mammography in an Australian mammographic screening programme. *J Med Screen*. 2000;7(4):190-4.
53. Offman J, Wilson M, Lamont M, et al. A randomised trial of weekend and evening breast screening appointments. *Br J Cancer*. 2013;109(3):597-602.
54. Oh D, Jung DW, Jun JK, et al. On-schedule mammography rescreening in the National Cancer Screening Program for breast cancer in Korea. *Asian Pac J Cancer Prev*. 2011;12(11):2865-70.
55. Ore L, Hagoel L, Shifroni G, et al. Compliance with mammography screening in Israeli women: the impact of a pre-scheduled appointment and of the letter-style. *Isr J Med Sci*. 1997;33(2):103-11.
56. O'Reilly D, Kinnear H, Rosato M, et al. Using record linkage to monitor equity and variation in screening programmes. *BMC Med Res Methodol*. 2012;12:59.
57. Ouédraogo S, Dabakuyo-Yonli TS, Roussot A, et al. European transnational ecological deprivation index and participation in population-based breast cancer screening programmes in France. *Prev Med*. 2014;63:103-8.
58. Peeters PH, Beckers CG, Hogervorst JM, et al. Effect on breast cancer screening response in The Netherlands of inviting women for an additional scientific investigation. *J Epidemiol Community Health*. 1994;48(2):175-7.
59. Pelfrene E, Bleyen L, De Backer G. Uptake in breast cancer screening: A sociogeographical analysis. *The European Journal of Public Health*. 1998;8.
60. Pinckney RG, Geller BM, Burman M, et al. Effect of false-positive mammograms on return for subsequent screening mammography. *Am J Med*. 2003;114(2):120-5.
61. Pornet C, Dejardin O, Morlais F, et al. Socioeconomic and healthcare supply statistical determinants of compliance to mammography screening programs: A multilevel analysis in Calvados, France. *Cancer Epidemiol*. 2010;34(3):309-15.
62. Renshaw C, Jack RH, Dixon S, et al. Estimating attendance for breast cancer screening in ethnic groups in London. *BMC Public Health*. 2010;10:157.
63. Richards SH, Bankhead C, Peters TJ, et al. Cluster randomised controlled trial comparing the effectiveness and cost-effectiveness of two primary care interventions aimed at improving attendance for breast screening. *J Med Screen*. 2001;8(2):91-8.
64. Rodriguez C, Plasencia A, Schroeder DG. Predictive factors of enrollment and adherence in a breast cancer screening program in Barcelona (Spain). *Soc Sci Med*. 1995;40(8):1155-60.
65. Scaf-Klomp W, van Sonderen FL, Stewart R, et al. Compliance after 17 years of breast cancer screening. *J Med Screen*. 1995;2(4):195-9.

For peer review only - http://bmjopen.bmj.com/site/about/guidelines.xhtml

66. Segnan N, Senore C, Giordano L, et al. Promoting participation in a population screening program for breast and cervical cancer: a randomized trial of different invitation strategies. *Tumori*. 1998;84(3):348-53.
67. Sim MJ, Siva SP, Ramli IS, et al. Effect of false-positive screening mammograms on rescreening in Western Australia. *Med J Aust*. 2012;196(11):693-5.
68. Simon MS, Gimotty PA, Moncrease A, et al. The effect of patient reminders on the use of screening mammography in an urban health department primary care setting. *Breast Cancer Res Treat*. 2001;65(1):63-70.
69. St-Jacques S, Philibert MD, Langlois A, et al. Geographic access to mammography screening centre and participation of women in the Quebec Breast Cancer Screening Programme. *J Epidemiol Community Health*. 2013;67(10):861-7.
70. Sutradhar R, Gu S, Glazier RH, et al. The association between visiting a primary care provider and uptake of periodic mammograms as women get older. *J Med Screen*. 2016;23(2):83-8.
71. Szczepura A, Price C, Gumber A. Breast and bowel cancer screening uptake patterns over 15 years for UK south Asian ethnic minority populations, corrected for differences in socio-demographic characteristics. *BMC Public Health*. 2008;8:346.
72. Taplin SH, Anderman C, Grothaus L, et al. Using physician correspondence and postcard reminders to promote mammography use. *Am J Public Health*. 1994;84(4):571-4.
73. Tatla RK, Paszat LF, Bondy SJ, et al. Socioeconomic status & returning for a second screen in the Ontario breast screening program. *Breast*. 2003;12(4):237-46.
74. Taylor V, Thompson B, Lessler D, et al. A clinic-based mammography intervention targeting inner-city women. *J Gen Intern Med*. 1999;14(2):104-11.
75. Taylor-Phillips S, O'Sullivan E, Kearins O, et al. The effects of a UK review of Breast Cancer Screening on Uptake: an observational before/after study. *J Med Screen*. 2013;20(2):86-90.
76. Vermeer B, Van den Muijsenbergh ME. The attendance of migrant women at the national breast cancer screening in the Netherlands 1997-2008. *Eur J Cancer Prev*. 2010;19(3):195-8.
77. Vidal C, Garcia M, Benito L, et al. Use of text-message reminders to improve participation in a population-based breast cancer screening program. *J Med Syst*. 2014;38(9):118.
78. Visser O, van Peppen AM, Ory FG, et al. Results of breast cancer screening in first generation migrants in Northwest Netherlands. *Eur J Cancer Prev*. 2005;14(3):251-5.
79. von Euler-Chelpin M, Olsen AH, Njor S, et al. Socio-demographic determinants of participation in mammography screening. *Int J Cancer*. 2008;122(2):418-23.
80. Wilf-Miron R, Peled R, Yaari E, et al. The association between socio-demographic characteristics and adherence to breast and colorectal cancer screening: Analysis of large sub populations. *BMC Cancer*. 2011;11(1):376.
81. Williams EM, Vessey MP. Randomised trial of two strategies offering women mobile screening for breast cancer. *BMJ*. 1989;299(6692):158-9.
82. Yarnall KS, Michener JL, Broadhead WE, et al. Increasing compliance with mammography recommendations: health assessment forms. *J Fam Pract*. 1993;36(1):59-64.
83. Zackrisson S, Lindström M, Moghaddassi M, et al. Social predictors of non-attendance in an urban mammographic screening programme: A multilevel analysis. *Scand J Soc Med*. 2007;35(5):548-54.
84. Zidar MN, Larm P, Tillgren P, et al. Non-attendance of mammographic screening: the roles of age and municipality in a population-based Swedish sample. *International Journal for Equity in Health*. 2015;14:157.
85. Chan W, Yun L, Austin PC, et al. Impact of socio-economic status on breast cancer screening in women with diabetes: a population-based study. *Diabet Med*. 2014;31(7):806-12.
86. Chochinov HM, Martens PJ, Prior HJ, et al. Does a diagnosis of schizophrenia reduce rates of mammography screening? A Manitoba population-based study. *Schizophr Res*. 2009;113(1):95-100.

For peer review only - http://bmjopen.bmj.com/site/about/guidelines.xhtml

87. Vigod SN, Kurdyak PA, Stewart DE, et al. Depressive symptoms as a determinant of breast and cervical cancer screening in women: a population-based study in Ontario, Canada. *Archives of Women's Mental Health*. 2011;14(2):159-68.
88. Aro AR, Koning HJ, Absetz P, et al. Psychosocial predictors of first attendance for organised mammography screening. J Med Screen [Internet]. 1999; 6(2):[82-8 pp.]. Available from: http://onlinelibrary.wiley.com/o/cochrane/clcentral/articles/380/CN-00166380/frame.html.
89. Jensen LF, Pedersen AF, Andersen B, et al. Social support and non-participation in breast cancer screening: a Danish cohort study. *Journal of Public Health*. 2016;38(2):335-42.
90. Lagerlund M, Sparén P, Thurfjell E, et al. Predictors of non-attendance in a population-based mammography screening programme; socio-demographic factors and aspects of behaviour. *Eur J Can Prev*. 2000;9.
91. Ulcickas Yood M, McCarthy BD, Lee NC, et al. Patterns and characteristics of repeat mammography among women 50 years and older. *Cancer Epidemiol Biomarkers Prev*. 1999;8(7):595-9.
92. Dryden R, Williams B, McCowan C, et al. What do we know about who does and does not attend general health checks? Findings from a narrative scoping review. *BMC Public Health*. 2012;12(1):723.
93. Thomas VN, Saleem T, Abraham R. Barriers to effective uptake of cancer screening among Black and minority ethnic groups. *Int J Palliat Nurs*. 2005;11(11):562, 4-71.
94. Long H, Brooks JM, Harvie M, et al. How do women experience a false-positive test result from breast screening? A systematic review and thematic synthesis of qualitative studies. *Br J Cancer*. 2019;121(4):351-8.

FIGURE LEGEND

Figure 1. PRISMA flow diagram: Shows the process of study flow and reasons for exclusion. The searches of electronic databases identified 11,949 unique publications (after deduplication), published between January 1987 and June 2019, of which 11,618 were excluded at the level of abstract/title screening, leaving 331 records for full text review. Of the 331 full texts, 66 unique studies reported in 67 publications were included.

Figure 2. Overall summary of QUIPS risk of bias scores: Risk of bias (RoB) of all included studies was appraised by two independent reviewers using the Quality in Prognosis Studies (QUIPS) tool. The QUIPS tool covers six RoB domains (participation, attrition, prognostic factor, confounding factors, outcome measurement and analysis and reporting), each of which includes multiple items that are judged separately. A conclusive judgment for each RoB domain is reached and expressed on a three-grade scale (high, moderate or low RoB). RoB across studies was generally low on all domains.

Figure 3. Meta-analyses
Legend: OR=odds ratio; CI=confidence interval; I^2=I-squared
This figure shows comparisons of the odds of attending mammographic screening, using random effects analysis, in observational studies by the following variables. Points to the left of the centre line (<1) suggest a lower likelihood of attending screening, while points to the right of the centre line (>1) indicate a higher likelihood of attending.

Age bands: we compared the age bands most commonly eligible for national screening programmes (60—69 and 50—59); there was no significant difference by age group (n = 16; OR 0.97, 95% CI: 0.88–1.08, p = 0.631);

For peer review only - http://bmjopen.bmj.com/site/about/guidelines.xhtml

For peer review only

Home ownership: we compared people who own their homes to those who are tenants or do not own their homes; the odds of attending were higher for homeowners than for tenants or non-owners (n = 3; OR 2.16, 95%CI: 2.08–2.23, p < 0.001);

Immigrant status: we compared screening attendance of people born in the country in which the study took place (non-immigrants) to those born in another country (immigrants); non-immigrants were more likely to attend than immigrants (n = 7; OR 2.23, 95% CI: 2.00–2.48 , p < 0.001).

Marital status: we compared women who were married or cohabiting to those who were unmarried or not cohabiting: women where were married/cohabiting were more likely to attend than their unmarried/non-cohabiting counterparts (n = 7; OR 1.86, 95% CI: 1.58–2.19 , p <0.001).

Reattendance; using data from studies with samples made up only of women who had previously attended mammographic screening, we compared women who had previously received a false-positive to those who had had a normal result; those with a previous false-positive result were less likely to reattend (OR 0.78, 95%CI: 0.68–0.88, p <0.001).

Figure 4. Meta-analyses of attendance by educational level, socioeconomic status (SES) and income

Legend: SES=socioeconomic status
These figures show random effects meta-analyses of screening attendance by educational level and socioeconomic status in observational studies. Points to the left of the centre line (<1) suggest a lower likelihood of attending screening, while points to the right of the centre line (>1) indicate a higher likelihood of attending.

Figure 4A shows the effects of different levels of education on screening attendance. We grouped education data to approximate the United Nations Educational, Scientific and Cultural Organization (UNESCO) three-level classification: low (≤10 years), middle (11–15 years) and high (>15 years). Compared to women with a low level of education, women with a medium level were more likely to attend (OR 1.24, 95% CI: 1.09–1.41, p < 0.001). Results from comparisons of women with a high level of education versus low or medium levels were not statistically significant (Figure 4A).

Figure 4B shows the meta-analysis of attendance by overall SES. Studies were grouped into low, medium and high categories. Women with medium or high SES were more likely to attend than those with a low SES (medium vs low SES OR 1.45, 95% CI: 1.20–1.75, p < 0.001; high vs low SES OR 1.69, 95% CI: 1.40–2.05, p < 0.001, Figure 4B).

Figure 4C shows the meta-analysis of screening attendance by income. Studies were grouped into low, intermediate and high categories. Women with an intermediate or high income were more likely to attend than those with low income (intermediate vs low income OR 1.96, 95% CI: 1.68–2.29, p < 0.001; high vs low OR 2.18, 95% CI: 1.86–2.56, p < 0.001; high vs intermediate OR 1.11, 95% CI: 0.95-1.30, p = 0.20, Figure 4C).

For both income and SES there was no significant difference between women at intermediate and high levels, indicating that there was no statistically significant dose response effect for higher SES or income.

For peer review only - http://bmjopen.bmj.com/site/about/guidelines.xhtml

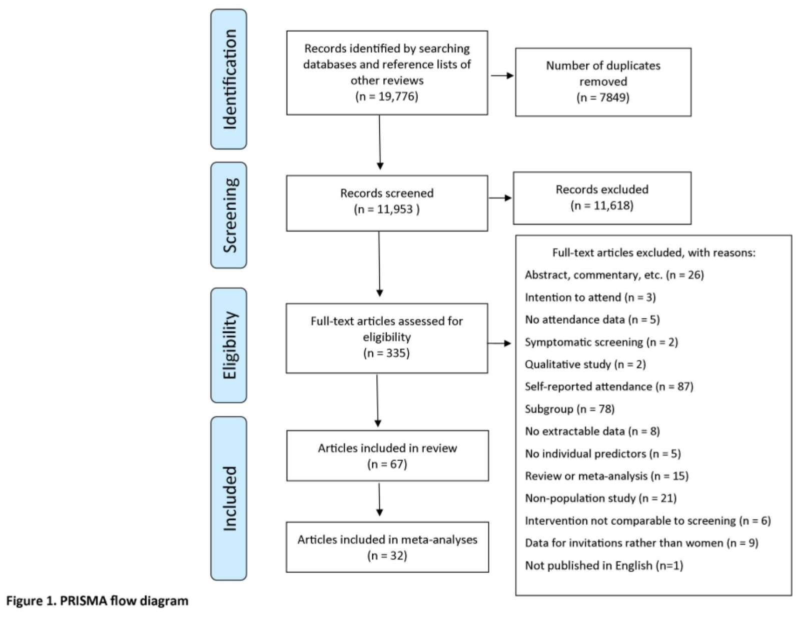

Figure 1. PRISMA flow diagram: Shows the process of study flow and reasons for exclusion. The searches of electronic databases identified 11,949 unique publications (after deduplication), published between January 1987 and June 2019, of which 11,618 were excluded at the level of abstract/title screening, leaving 331 records for full text review. Of the 331 full texts, 66 unique studies reported in 67 publications were included.

297x209mm (300 x 300 DPI)

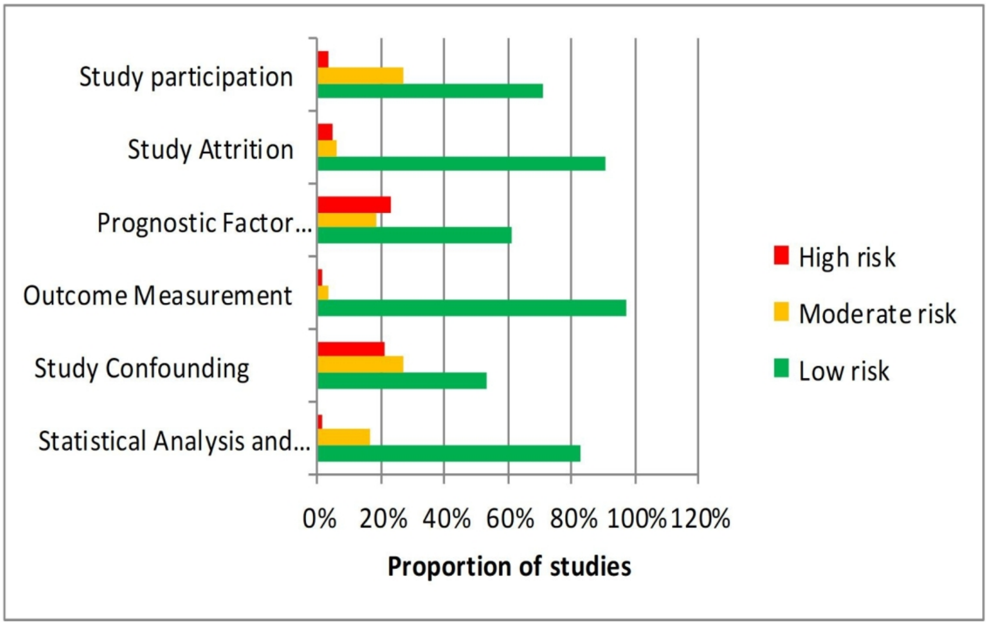

Figure 2. Overall summary of QUIPs risk of bias scores

Figure 2. Overall summary of QUIPS risk of bias scores: Risk of bias (RoB) of all included studies was appraised by two independent reviewers using the Quality in Prognosis Studies (QUIPS) tool. The QUIPS tool covers six RoB domains (participation, attrition, prognostic factor, confounding factors, outcome measurement and analysis and reporting), each of which includes multiple items that are judged separately. A conclusive judgment for each RoB domain is reached and expressed on a three-grade scale (high, moderate or low RoB). RoB across studies was generally low on all domains.

146x102mm (300 x 300 DPI)

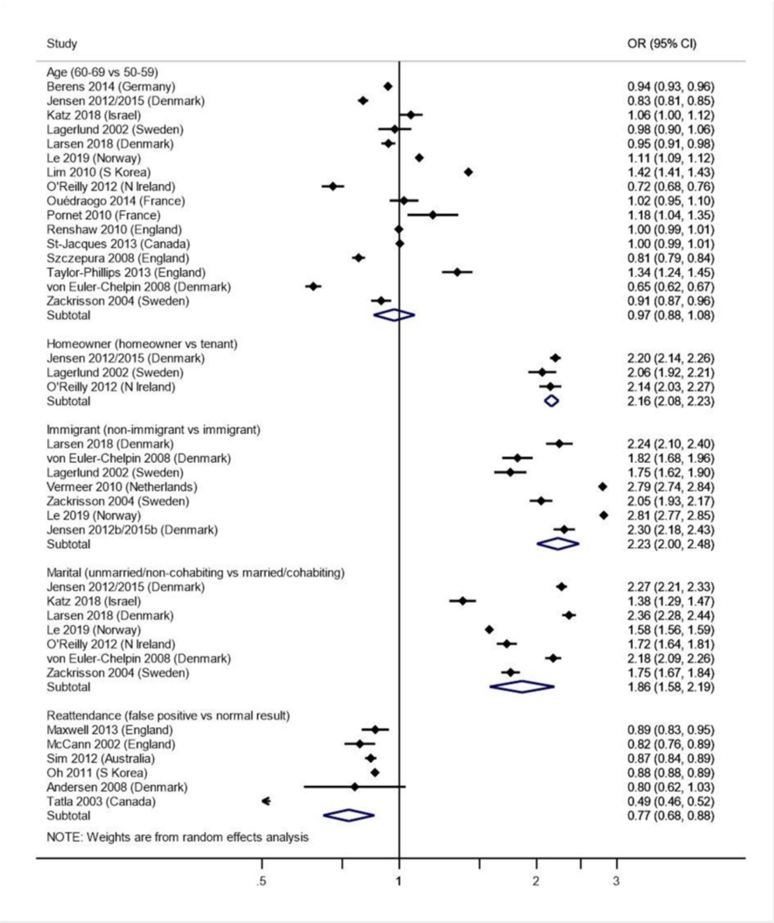

Figure 3. Meta-analyses: attendance by age, home ownership, immigrant status, marriage and re-attendance

Figure 3. Meta-analyses
Legend: OR=odds ratio; CI=confidence interval; I2=I-squared
This figure shows comparisons of the odds of attending mammographic screening, using random effects analysis, in observational studies by the following variables. Points to the left of the centre line (<1) suggest a lower likelihood of attending screening, while points to the right of the centre line (>1) indicate a higher likelihood of attending.
Age bands: we compared the age bands most commonly eligible for national screening programmes (60—69 and 50—59); there was no significant difference by age group (n = 16; OR 0.97, 95% CI: 0.88–1.08, p = 0.631);
Home ownership: we compared people who own their homes to those who are tenants or do not own their homes; the odds of attending were higher for homeowners than for tenants or non-owners (n = 3; OR 2.16, 95%CI: 2.08–2.23, p < 0.001);
Immigrant status: we compared screening attendance of people born in the country in which the study took place (non-immigrants) to those born in another country (immigrants); non-immigrants were more likely to

attend than immigrants (n = 7; OR 2.23, 95% CI: 2.00–2.48 , $p < 0.001$).
Marital status: we compared women who were married or cohabiting to those who were unmarried or not cohabiting: women where were married/cohabiting were more likely to attend than their unmarried/non-cohabiting counterparts (n = 7; OR 1.86, 95% CI: 1.58–2.19 , $p < 0.001$).
Reattendance; using data from studies with samples made up only of women who had previously attended mammographic screening, we compared women who had previously received a false-positive to those who had had a normal result; those with a previous false-positive result were less likely to reattend (OR 0.78, 95%CI: 0.68–0.88, $p < 0.001$).

209x297mm (300 x 300 DPI)

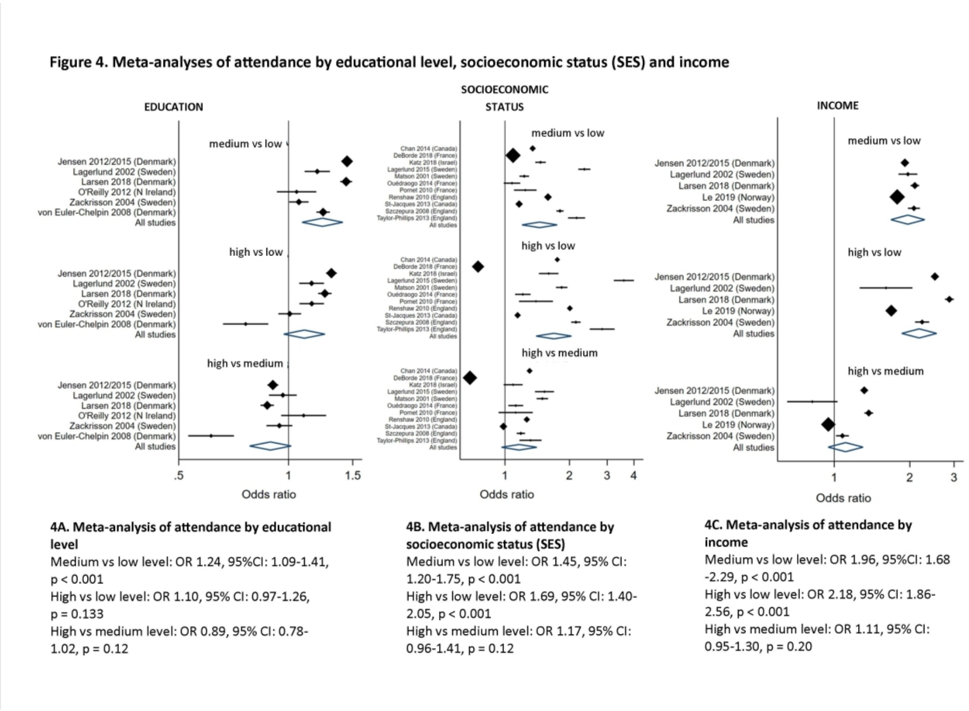

Figure 4. Meta-analyses of attendance by educational level, socioeconomic status (SES) and income
Legend: SES=socioeconomic status
These figures show random effects meta-analyses of screening attendance by educational level and socioeconomic status in observational studies. Points to the left of the centre line (<1) suggest a lower likelihood of attending screening, while points to the right of the centre line (>1) indicate a higher likelihood of attending.
Figure 4A shows the effects of different levels of education on screening attendance. We grouped education data to approximate the United Nations Educational, Scientific and Cultural Organization (UNESCO) three-level classification: low (≤10 years), middle (11–15 years) and high (>15 years). Compared to women with a low level of education, women with a medium level were more likely to attend (OR 1.24, 95% CI: 1.09–1.41, p < 0.001). Results from comparisons of women with a high level of education versus low or medium levels were not statistically significant (Figure 4A).
Figure 4B shows the meta-analysis of attendance by overall SES. Studies were grouped into low, medium and high categories. Women with medium or high SES were more likely to attend than those with a low SES (medium vs low SES OR 1.45, 95% CI: 1.20–1.75, p < 0.001; high vs low SES OR 1.69, 95% CI: 1.40–2.05, p < 0.001, Figure 4B).
Figure 4C shows the meta-analysis of screening attendance by income. Studies were grouped into low, intermediate and high categories. Women with an intermediate or high income were more likely to attend than those with low income (intermediate vs low income OR 1.96, 95% CI: 1.68–2.29, p < 0.001; high vs low OR 2.18, 95% CI: 1.86–2.56, p < 0.001; high vs intermediate OR 1.11, 95% CI: 0.95-1.30, p = 0.20, Figure 4C).
For both income and SES there was no significant difference between women at intermediate and high levels, indicating that there was no statistically significant dose response effect for higher SES or income.

297x209mm (300 x 300 DPI)

PROSPERO
International prospective register of systematic reviews

A systematic review to identify the worldwide predictors of breast screening uptake

Rebecca Crosby, Sian Williamson, Chris Stinton, Aileen Clarke, Sian Taylor-Phillips

Citation

Rebecca Crosby, Sian Williamson, Chris Stinton, Aileen Clarke, Sian Taylor-Phillips. A systematic review to identify the worldwide predictors of breast screening uptake. PROSPERO 2016 CRD42016051597 Available from: https://www.crd.york.ac.uk/prospero/display_record.php?ID=CRD42016051597

Review question

What are the predictors of breast cancer screening uptake worldwide?

Searches

The electronic databases that will be searched to identify published studies are:
EMBASE (via Ovid), MEDLINE (via Ovid), CINAHL (The Cumulative Index to Nursing and Allied Health Literature), PsycINFO, Cochrane Library (Wiley) including the Cochrane Database of Systematic Reviews and Thomson Reuters Web of Science (all databases including Science Citation Index, Conference Proceedings and Science Citation Index Expanded and Social Sciences Index).
Reference lists of included papers and relevant reviews will be searched for papers that were not identified by the electronic search.
Experts in the field will be contacted to identify further significant papers.

Types of study to be included

Included:Any quantitative study type that mentions uptake rates of breast screening;Study must include at least one predictor of uptake to be included.Excluded:Case studies, editorials, letters and commentaries.

Condition or domain being studied

Breast screening. Predictors of uptake.

Participants/population

Inclusion:
Women of screening age (variable worldwide).
Exclusion:
Women with previously diagnosed breast cancer;
Women attending diagnostic screening;
Non-human studies.

Intervention(s), exposure(s)

Any intervention related to uptake of breast screening will be included in the review. Studies will be included where they mention uptake rates of breast screening - either current, previous or changes. The study must mention at least one predictor variable of uptake.

Comparator(s)/control

Not applicable.

Main outcome(s)

Worldwide predictors of uptake of breast screening.

* Measures of effect

The outcomes will be measured in terms of uptake, i.e. rate or percentages.

Additional outcome(s)

None.

For peer review only

* Measures of effect

Not applicable.

Data extraction (selection and coding)

A two-step process will be used to identify relevant studies at abstract and title stage and then at full text stage using pre-defined screening criteria. Two researchers will screening the titles and abstracts against inclusion and inclusion criteria independently using the results from the search. if a decision cannot be made on the title and abstract a full text review will be performed.
Where there are disagreements between the two researchers, a third reviewer will be contacted until a consensus is reached.
The full texts of the included studies will be obtained and undergo a second screen by two researchers and again any discrepancies resolved by the third reviewer.
Reasons for inclusion and exclusion will be stated where appropriate. The PRISMA flow diagram will be provided in the review.
Data extraction will take place after the full text review and will include:
General - authors, year, publication journal, study title, article type, stated aims, period of study;
Study characteristics - country, setting, screening programme style in this country;
Study design - cohort, case-control, prospective, retrospective, randomised controlled trial, etc.;
Participants - population;
Outcomes - primary and secondary outcomes definitions, validity of measures used, data collection method;
Predictors - number of predictors, type of predictors, definition of predictors;
overall results;
Overview - strengths and limitations of the study, Was the study blinded?; source(s) of research funding, potential conflicts of interest.
The domains involved in data extraction are broad and comprehensive due to the variability of the potential studies to be included within this review. A piloted data extraction form will be used by the two researchers to test. Any discrepancies will be discussed and a third reviewer will be involved where necessary to reach a consensus.

Risk of bias (quality) assessment

Extracted data will be stored in tabular format on Microsoft Access spreadsheet to complete the methodological quality assessment/risk of bias scoring.
Quality assessment of the included studies will be completed using the quality assessment tool.

Strategy for data synthesis

Descriptive analysis will be presented in tabular format to describe the included studies. Significant hetergeneity is expected to be found amongst the included papers considering the differences between screening programmes internationally. Therefore pooling data in a meta-regression would not be appropriate. Instead, a narrative synthesis will be adopted to explain and summarise results by predictor. This narrative synthesis will analyse the population characteristics, predictor variables and their effects on uptake rate.

Analysis of subgroups or subsets

None planned.

Contact details for further information

Rebecca Crosby
R.Crosby@warwick.ac.uk

Organisational affiliation of the review

University of Warwick

Review team members and their organisational affiliations

Miss Rebecca Crosby. University of Warwick
Miss Sian Williamson. University of Warwick
Dr Chris Stinton. University of Warwick
Professor Aileen Clarke. University of Warwick

For peer review only

Dr Sian Taylor-Phillips. University of Warwick

Type and method of review
Systematic review

Anticipated or actual start date
21 November 2016

Anticipated completion date
31 July 2017

Funding sources/sponsors
This systematic review presents independent research funded by the NIHR Collaboration for Leadership in Applied Health Research and Care West Midlands (CLAHRC-WM) initiative. The views expressed are those of the author(s) and not necessarily those of the NHS, the NIHR or the Department of Health.

Conflicts of interest
None known

Language
English

Country
England

Stage of review
Review Ongoing

Subject index terms status
Subject indexing assigned by CRD

Subject index terms
Breast Neoplasms; Developed Countries; Developing Countries; Early Detection of Cancer; Early Diagnosis; Healthcare Disparities; Humans; Mass Screening; Patient Acceptance of Health Care; Socioeconomic Factors

Date of registration in PROSPERO
17 November 2016

Date of first submission

Stage of review at time of this submission

Stage	Started	Completed
Preliminary searches	No	No
Piloting of the study selection process	No	No
Formal screening of search results against eligibility criteria	No	No
Data extraction	No	No
Risk of bias (quality) assessment	No	No
Data analysis	No	No

Revision note
Updated the prospero registration to be more accurate. Updated inclusion criteria to only include quantitative studies due to the number of results found after sifting.

For peer review only

The record owner confirms that the information they have supplied for this submission is accurate and complete and they understand that deliberate provision of inaccurate information or omission of data may be construed as scientific misconduct.

The record owner confirms that they will update the status of the review when it is completed and will add publication details in due course.

Versions

17 November 2016
23 June 2017

PROSPERO
This information has been provided by the named contact for this review. CRD has accepted this information in good faith and registered the review in PROSPERO. The registrant confirms that the information supplied for this submission is accurate and complete. CRD bears no responsibility or liability for the content of this registration record, any associated files or external websites.

For peer review only

Supplementary file B: Search strategy

Database: Ovid MEDLINE(R) <1946 onwards>

Search Strategy:

----

1 exp Breast/ or breast*.mp.

2 (screen* or early detection or mammogra* or mass screening or screening program* or mammogra* screen* or direct to consumer or health screen*).mp. [mp=title, abstract, original title, name of substance word, subject heading word, floating sub-heading word, keyword heading word, organism supplementary concept word, protocol supplementary concept word, rare disease supplementary concept word, unique identifier, synonyms]

3 (uptake or adheren* or complian* or patient acceptance of healthcare or patient acceptance or patient access or attend*).mp. [mp=title, abstract, original title, name of substance word, subject heading word, floating sub-heading word, keyword heading word, organism supplementary concept word, protocol supplementary concept word, rare disease supplementary concept word, unique identifier, synonyms]

4 1 and 2 and 3

5 limit 4 to humans

6 limit 5 to english language

Database: Ovid MEDLINE(R) Daily Update, Ovid MEDLINE(R) Epub Ahead of Print and In-Process, In-Data-Review & Other Non-Indexed Citations

Search Strategy:

----

1 exp Breast/ or breast*.mp.

2 (screen* or early detection or mammogra* or mass screening or screening program* or mammogra* screen* or direct to consumer or health screen*).mp. [mp=title, abstract, original title, name of substance word, subject heading word, floating sub-heading word, keyword heading word, organism supplementary concept word, protocol supplementary concept word, rare disease supplementary concept word, unique identifier, synonyms]

3 (uptake or adheren* or complian* or patient acceptance of healthcare or patient acceptance or patient access or attend*).mp. [mp=title, abstract, original title, name of substance word, subject heading word, floating sub-heading word, keyword heading word, organism supplementary concept

For peer review only

word, protocol supplementary concept word, rare disease supplementary concept word, unique identifier, synonyms]

4 1 and 2 and 3

Database: Embase Classic+Embase <1947 onwards>

Search Strategy:

----

1 exp Breast/ or breast*.mp.

2 (screen* or early detection or mammogra* or mass screening or screening program* or mammogra* screen* or direct to consumer or health screen*).mp. [mp=title, abstract, heading word, drug trade name, original title, device manufacturer, drug manufacturer, device trade name, keyword, floating subheading word, candidate term word]

3 (uptake or adheren* or complian* or patient acceptance of healthcare or patient acceptance or patient access or attend*).mp. [mp=title, abstract, heading word, drug trade name, original title, device manufacturer, drug manufacturer, device trade name, keyword, floating subheading word, candidate term word]

4 1 and 2 and 3

5 limit 4 to humans

6 limit 5 to english language

Database: APA PsycInfo <1806 onwards>

Search Strategy:

----

1 breast.mp. or exp Breast/

2 (screen* or early detection or mammogra* or mass screening or screening program* or mammogra* screen* or direct to consumer or health screen*).mp. [mp=title, abstract, heading word, table of contents, key concepts, original title, tests & measures, mesh] (132904)

3 (uptake or adheren* or complian* or "patient acceptance of healthcare" or "patient acceptance" or attend*).mp. [mp=title, abstract, heading word, table of contents, key concepts, original title, tests & measures, mesh]

4 1 and 2 and 3

For peer review only

5 limit 4 to yr="2017 -Current"

6 limit 5 to human

7 limit 6 to english language

Database: Web of Science, 1900 onwards

TOPIC: (breast*) *AND* **TOPIC:** (screen* or "early detection" or mammogra* or "mass screening" or "screening program*" or "mammogra* screen*" or "direct to consumer" or "health screen") *AND* **TOPIC:** (uptake or adheren* or complian* or "patient acceptance of healthcare" or "patient acceptance" or "patient access" or attend*)

Database: CINAHL (Limiters - Publication Year: 1987-June 2019)

S1. TX breast’

S2. TX screen’ OR early detection OR TX mammogra’ Or TX mass screening OR TX screening program’ OR TX mammogra’ screen’ OR TX direct to consumer OR TX health screen’

S3. TX uptake OR TX adheren’ OR TX complian’ OR TX patient acceptance of health care OR TX patient acceptance OR TX patient access OR TX health screen’

S4. S1 AND S2 AND S3

Database: Cochrane Library

S1. TX breast’

S2. TX screen’ OR early detection OR TX mammogra’ Or TX mass screening OR TX screening program’ OR TX mammogra’ screen’ OR TX direct to consumer OR TX health screen’

S3. TX uptake OR TX adheren’ OR TX complian’ OR TX patient acceptance of health care OR TX patient acceptance OR TX patient access OR TX health screen’

S4. S1 AND S2 AND S3

For peer review only

PRISMA 2009 Checklist

Section/topic	#	Checklist item	Reported on page #
**TITLE**			
Title	1	Identify the report as a systematic review, meta-analysis, or both.	1
**ABSTRACT**			
Structured summary	2	Provide a structured summary including, as applicable: background; objectives; data sources; study eligibility criteria, participants, and interventions; study appraisal and synthesis methods; results; limitations; conclusions and implications of key findings; systematic review registration number.	2
**INTRODUCTION**			
Rationale	3	Describe the rationale for the review in the context of what is already known.	4
Objectives	4	Provide an explicit statement of questions being addressed with reference to participants, interventions, comparisons, outcomes, and study design (PICOS).	5
**METHODS**			
Protocol and registration	5	Indicate if a review protocol exists, if and where it can be accessed (e.g., Web address), and, if available, provide registration information including registration number.	4-5
Eligibility criteria	6	Specify study characteristics (e.g., PICOS, length of follow-up) and report characteristics (e.g., years considered, language, publication status) used as criteria for eligibility, giving rationale.	5
Information sources	7	Describe all information sources (e.g., databases with dates of coverage, contact with study authors to identify additional studies) in the search and date last searched.	5
Search	8	Present full electronic search strategy for at least one database, including any limits used, such that it could be repeated.	Supplementary file B
Study selection	9	State the process for selecting studies (i.e., screening, eligibility, included in systematic review, and, if applicable, included in the meta-analysis).	5
Data collection process	10	Describe method of data extraction from reports (e.g., piloted forms, independently, in duplicate) and any processes for obtaining and confirming data from investigators.	5–6
Data items	11	List and define all variables for which data were sought (e.g., PICOS, funding sources) and any assumptions and simplifications made.	5–6
Risk of bias in individual studies	12	Describe methods used for assessing risk of bias of individual studies (including specification of whether this was done at the study or outcome level), and how this information is to be used in any data synthesis.	6
Summary measures	13	State the principal summary measures (e.g., risk ratio, difference in means).	6
Synthesis of results	14	Describe the methods of handling data and combining results of studies, if done, including measures of consistency (e.g., I^2) for each meta-analysis.	6

For peer review only

For peer review only - http://bmjopen.bmj.com/site/about/guidelines.xhtml

PRISMA 2009 Checklist

Section/topic	#	Checklist item	Reported on page #
Risk of bias across studies	15	Specify any assessment of risk of bias that may affect the cumulative evidence (e.g., publication bias, selective reporting within studies).	6
Additional analyses	16	Describe methods of additional analyses (e.g., sensitivity or subgroup analyses, meta-regression), if done, indicating which were pre-specified.	6
**RESULTS**			
Study selection	17	Give numbers of studies screened, assessed for eligibility, and included in the review, with reasons for exclusions at each stage, ideally with a flow diagram.	7, Figure 1
Study characteristics	18	For each study, present characteristics for which data were extracted (e.g., study size, PICOS, follow-up period) and provide the citations.	Supplementary file D
Risk of bias within studies	19	Present data on risk of bias of each study and, if available, any outcome level assessment (see item 12).	8, Figure 2
Results of individual studies	20	For all outcomes considered (benefits or harms), present, for each study: (a) simple summary data for each intervention group (b) effect estimates and confidence intervals, ideally with a forest plot.	Table 1, Figures 3 & 4, Table 2
Synthesis of results	21	Present results of each meta-analysis done, including confidence intervals and measures of consistency.	Table 1
Risk of bias across studies	22	Present results of any assessment of risk of bias across studies (see Item 15).	8
Additional analysis	23	Give results of additional analyses, if done (e.g., sensitivity or subgroup analyses, meta-regression [see Item 16]).	10, 14
**DISCUSSION**			
Summary of evidence	24	Summarize the main findings including the strength of evidence for each main outcome; consider their relevance to key groups (e.g., healthcare providers, users, and policy makers).	14–15
Limitations	25	Discuss limitations at study and outcome level (e.g., risk of bias), and at review-level (e.g., incomplete retrieval of identified research, reporting bias).	16–17
Conclusions	26	Provide a general interpretation of the results in the context of other evidence, and implications for future research.	17–18
**FUNDING**			
Funding	27	Describe sources of funding for the systematic review and other support (e.g., supply of data); role of funders for the systematic review.	18–19

From: Moher D, Liberati A, Tetzlaff J, Altman DG, The PRISMA Group (2009). Preferred Reporting Items for Systematic Reviews and Meta-Analyses: The PRISMA Statement. PLoS Med 6(7): e1000097. doi:10.1371/journal.pmed1000097

For peer review only - http://bmjopen.bmj.com/site/about/guidelines.xhtml

For peer review only

Supplementary file D: Characteristics of included studies

*indicates studies that could be included in meta-analysis or narrative analysis

Author	Publication year	Study country	N in analysis	Study start	Study end	Study design	Factors analysed	Odds ratio (95%CI)
Allgood	2016	UK	22828	unclear	unclear	RCT	NA	NA
Andersen*	2008	Denmark	22653	1999	2001	retrospective cohort	Previous result of mammogram	0.80 (0.62–1.03)
Barlow	2019	USA	305568	2012	2013	cohort	Age	NA
Berens*	2014	Germany	423649	2010	2011	cohort	Age	0.94 (0.93–0.96)
Blanchard*	2004	USA	18851	1985	2002	retrospective cohort	Non-English language spoken<br><br>Member of majority racial/ethnic group	0.33 (0.28–0.39)<br><br>1.70 (1.52–1.89)
Bourmaud	2016	France	15844	2009	2009	RCT	NA	NA
Chan*	2014	Canada	504288	1999	2010	retrospective cohort	SES:<br>Medium vs low<br>High vs low<br>Medium vs high<br><br>Diabetes	 1.35 (1.33–1.37)<br>1.76 (1.72–1.79)<br>1.30 (1.28–1.32)<br><br>0.79 (0.78–0.80)
Chiarelli*	2003	Canada	125250	1990	1995	retrospective cohort	Age at initial screen	0.99 (0.96–1.02)
Chochinov*	2009	Canada	110240	2002	2004	retrospective cohort	Schizophrenia	0.58 (0.52–0.64)
DeBorde*	2018	France	4805390	2013	2014	cohort	SES:<br>Medium vs low<br>High vs low<br>Medium vs high	 1.09 (1.09–1.10)<br>0.75 (0.74–0.75)<br>0.69 (0.68–0.69)
Douglas	2016	UK	NA	2012	2012	cohort	NA	NA

For peer review only - http://bmjopen.bmj.com/site/about/guidelines.xhtml

Finney Rutten	2014	USA	62754	2004	2005	cohort	NA	NA
Gatrell	1998	UK	24000	1988	1995	cohort	NA	NA
Goldzahl	2018	France	26495	2015	2015	RCT	NA	NA
Hyndman	2000	Australia	5968	1991	1996	retrospective cohort	NA	NA
Jensen*	2012b/2015b	Denmark	144264	2008	2009	cohort	Education:	
							Medium vs low	1.45 (1.41–1.49)
							High vs low	1.31 (1.27–1.36)
							Medium vs high	0.91 (0.88–0.94)
							Housing tenure (homeowner vs non)	2.20 (2.14–2.26)
							Marital status (non-married/non-cohabiting vs married/cohabiting)	2.27 (2.21–2.33)
							Income:	
							Intermediate vs low	1.91 (1.85–1.97)
							High vs low	2.52 (2.44–2.60)
							Intermediate vs high	1.32 (1.27–1.36)
Katz*	2018	Israel	44318	2008	2014	cohort	Age	1.06 (1.00–1.12)
							Current smoker	0.72 (0.65–0.79)
							60+ primary care visits during 6-year study period (vs <60)	2.70 (2.55–2.86)
							Depression	1.12 (1.02–1.23)
							Diabetes	1.25 (1.17–1.33)

For peer review only - http://bmjopen.bmj.com/site/about/guidelines.xhtml

For peer review only

							Heart disease	1.75 (1.61–1.91)
							BMI	0.95 (0.87–1.04)
Kee*	1993	Northern Ireland	600	1991	1991	cross-sectional	Negative attitude about breast screening	0.44 (0.35–0.55)
Lagerlund*	2002	Sweden	46041	1988	1997	cohort	Age	0.98 (0.90–1.06)
							Country of origin (immigrant vs non)	1.75 (1.62–1.90)
							Education:	
							Medium vs low	1.20 (1.11–1.30)
							High vs low	1.16 (1.07–1.25)
							Medium vs high	0.96 (0.88–1.05)
							Housing tenure (homeowner vs non)	2.06 (1.92–2.21)
							Income:	
							Intermediate vs low	1.96 (1.80–2.14)
							High vs low	1.61 (1.26–2.05)
							Intermediate vs high	0.82 (0.65–1.03)
							Number of childbirths:	
							0 vs 1–2	0.44 (0.40–0.48)
							3+ vs 1–2	0.81 (0.75–0.87)
Lagerlund*	2015	Sweden	46041	2005	2009	cohort	SES:	
							Medium vs low	2.35 (2.20–2.51)
							High vs low	3.59 (3.22–4.00)
							Medium vs high	1.53 (1.38–1.69)
Larsen*	2018	Denmark	91517	2008	2009	cohort	Age	0.95 (0.91–0.98)

For peer review only - http://bmjopen.bmj.com/site/about/guidelines.xhtml

							Country of origin (immigrant vs non)	2.24 (2.10–2.40)
							Education:	
							Medium vs low	1.44 (1.39–1.49)
							High vs low	1.26 (1.21–1.31)
							Medium vs high	0.87 (0.84–0.91)
							Income:	
							Intermediate vs low	2.09 (2.01–2.18)
							High vs low	2.87 (2.76–2.99)
							Intermediate vs high	1.37 (1.32–1.43)
							No comorbidities	1.53 (1.46–1.60)
Le*	2019	Norway	885979	1996	2015	cohort	Age	1.11 (1.09–1.12)
							Country of origin (immigrant vs non)	2.81 2.77–2.85)
							Marital status (non-married/non-cohabiting vs married/cohabiting)	1.58 (1.56–1.59)
							Income:	
							Intermediate vs low	1.78 (1.76–1.81)
							High vs low	1.69 (1.65–1.72)
							Intermediate vs high	0.95 (0.93–0.96)
							Receiving disability benefits	0.70 (0.70–0.71)
							Employment status (vs employed/self-employed):	

For peer review only

Breast cancer screening attendance factors: Systematic review

							Outside workforce	0.51 (0.50–0.51)
							Unemployed	0.47 (0.45–0.49)
							Not living in capital city	1.94 (1.91–1.97)
							Citizen of country	2.88 (2.82–2.94)
Leung*	2015	UK	27416	2008	20101	cohort	Residence (rural vs urban)	1.11 (1.04–1.19)
Lim*	2010	South Korea	3705246	2008	2008	cohort	Age	1.42 (1.41–1.43)
Luckman	2019	USA	10063	2010	2014	RCT	NA	NA
Makedonov*	2015	Canada	105665	2005	2011	case-control	Age at initial screen	1.05 (1.02–1.08)
							Physician years since graduation	1.03 (0.99–1.06)
Matson*	2001	Sweden	32605	1990	1994	cohort	SES:	
							Medium vs low	1.23 (1.17–1.30)
							High vs low	1.84 (1.73–1.96)
							Medium vs high	1.49 (1.41–1.59)
Maxwell*	2013	UK	253017	2005	2008	retrospective cohort	Previous result of mammogram	0.89 (0.83–0.95)
Mayer	2000	USA	1562	1995	1998	RCT	NA	NA
McCann*	2002	UK	113409	1989	1991	retrospective cohort	Previous result of mammogram	0.82 (0.76–0.89)
Meldrum	1994	UK	3083	1992	1993	RCT	NA	NA
Merrick	2015	USA	4427	2010	2012	RCT	NA	NA
Moss	2001	UK	210939	1996	unclear	retrospective cohort	NA	NA
O'Byrne*	2000	Australia	119502	1995	1996	retrospective cohort	No family history of breast cancer	0.90 (0.86–0.94)
							Type of clinic (mobile vs fixed)	0.93 (0.88–0.98)

For peer review only - http://bmjopen.bmj.com/site/about/guidelines.xhtml

							Never HRT use	1.13 (1.09–1.17)
Offman	2013	UK	12929	2010	2011	RCT	NA	NA
Oh*	2011	South Korea	2511976	2005	2008	retrospective cohort	Previous result of mammogram	0.88 (0.88–0.89)
Ore*	1997	Israel	736	1994	1994	RCT	Age	1.16 (0.83–1.62)
O'Reilly*	2012	Northern Ireland	37059	2001	2004	cohort	Age	0.72 (0.68–0.76)
							Education:	
							Medium vs low	1.05 (0.93–1.19)
							High vs low	1.16 (1.07–1.25)
							Medium vs high	1.10 (0.95–1.27)
							Housing tenure (homeowner vs non)	2.14 (2.03–2.27)
							Marital status (non-married/non-cohabiting vs married/cohabiting)	1.72 (1.64–1.81)
							Residence (rural vs urban)	1.59 (1.50–1.68)
							No access to vehicle	0.43 (0.41–0.46)
							Long-term limiting illness	0.71 (0.68–0.75)
							Good general health	1.55 (1.46–1.64)
							Religion:	
							Catholic vs none	1.40 (1.25–1.47)
							Protestant vs none	1.57 (1.46–1.70)
Ouédraogo*	2014	France	13565	2010	2011	cohort	Age	1.02 (0.95–1.10)
							Residence (rural vs urban)	0.80 (0.75–0.86)

For peer review only

							SES:	
							Medium vs low	1.08 (0.99–1.18)
							High vs low	1.21 (1.11–1.32)
							Medium vs high	1.12 (1.04–1.22)
							Distance to screening centre	0.85 (0.79–0.91)
Peeters*	1994	Netherlands	1863	1992	1992	RCT	Age	0.69 (0.57–0.84)
Pelfrene	1998	Belgium	40713	1992	1992	cohort	NA	NA
Pinckney	2003	USA	41844	1996	1997	retrospective cohort	NA	NA
Pornet*	2010	France	4865	2004	2006	cohort	Age	1.18 (1.04–1.35)
							SES:	
							Medium vs low	1.24 (1.10–1.40)
							High vs low	1.40 (1.16–1.67)
							Medium vs high	1.12 (0.93–1.35)
							>0 GPs per 100,000 inhabitants	0.96 (0.85–1.08)
							>0 radiologists per 100,000 inhabitants	0.87 (0.72–1.05)
Renshaw*	2010	UK	742786	2004	2007	cohort	Age	1.00 (0.99–1.01)
							SES:	
							Medium vs low	1.59 (1.57–1.61)
							High vs low	2.01 (1.98–2.04)
							Medium vs high	1.26 (1.25–1.28)
							First invitation to screening	0.22 (0.21–0.22)

For peer review only - http://bmjopen.bmj.com/site/about/guidelines.xhtml

Richards	2001	UK	5732	1997	1998	RCT	NA	NA
Rodriguez	1995	Spain	1859	1989	1989	cohort	NA	NA
Scaf-Klomp	1995	Netherlands	6898	1975	1990	cohort	NA	NA
Segnan	1998	Italy	8069	1993	1993	RCT	NA	NA
Sim*	2012	Australia	582729	1995	2007	retrospective cohort	Previous result of mammogram	0.87 (0.84–0.89)
Simon	2001	USA	1718	1992	1993	RCT	NA	NA
St-Jacques*	2013	Canada	833856	2006	2008	cohort	Age<br><br>SES:<br>Medium vs low<br>High vs low<br>Medium vs high<br><br>Distance to screening centre	1.00 (0.99–1.01)<br><br><br>1.16 (1.15–1.18)<br>1.15 (1.13–1.16)<br>0.98 (0.97–0.99)<br><br>1.02 (1.01–1.03)
Sutradhar	2016	Canada	2389889	2001	2010	retrospective cohort	NA	NA
Szczepura*	2008	UK	86211	2001	2004	cohort	Age<br><br>SES:<br>Medium vs low<br>High vs low<br>Medium vs high	0.81 (0.79–0.84)<br><br><br>1.81 (1.74–1.88)<br>2.14 (2.04–2.25)<br>1.19 (1.13–1.24)
Taplin	1994	USA	1322	unclear	unclear	RCT	NA	NA
Tatla*	2003	Canada	57201	1995	2005	retrospective cohort	Age at initial screen<br><br>Previous result of mammogram	0.93 (0.90–0.97)<br><br>0.49 (0.46–0.52)
Taylor*	1999	USA	82	1995	1996	RCT	Age	0.24 (0.05–1.13)
Taylor-Phillips*	2013	UK	11664	2012	2012	cohort	Age	1.34 (1.24–1.45)

For peer review only - http://bmjopen.bmj.com/site/about/guidelines.xhtml

							SES:<br>Medium vs low<br>High vs low<br>Medium vs high	 2.16 (1.97–2.37)<br>2.84 (2.49–3.25)<br>1.32 (1.17–1.48)
							Previous attender	3.32 (3.05–3.61)
Vermeer*	2010	Netherlands	1279982	2007	2008	cohort	Country of origin (immigrant vs non)	2.79 (2.74–2.84)
Vidal	2014	Spain	12475	2011	2011	quasi-experimental	NA	NA
Visser	2005	Netherlands	825523	1995	2002	retrospective cohort	NA	NA
von Euler-Chelpin*	2008	Denmark	73416	1991	1999	cohort	Age	0.65 (0.62–0.67)
							Country of origin (immigrant vs non)	1.82 (1.68–1.96)
							Education:<br>Medium vs low<br>High vs low<br>Medium vs high	 1.24 (1.19–1.30)<br>0.76 (0.66–0.88)<br>0.61 (0.53–0.71)
							Marital status (non-married/non-cohabiting vs married/cohabiting)	2.18 (2.09–2.27)
Wilf-Miron	2011	Israel	157928	2008	2008	retrospective cohort	NA	NA
Williams	1989	UK	392	unclear	unclear	RCT	NA	NA
Yarnall	1993	USA	unclear	1985	1988	case-control	NA	NA
Zackrisson*	2004	Sweden	33627	1990	1993	cohort	Age	0.91 (0.88–1.08)
							Country of origin (immigrant vs non)	2.05 (1.93–2.17)

For peer review only - http://bmjopen.bmj.com/site/about/guidelines.xhtml

							Education:	
							Medium vs low	1.07 (1.00–1.14)
							High vs low	1.01 (0.94–1.08)
							Medium vs high	0.94 (0.87–1.02)
							Marital status (non-married/non-cohabiting vs married/cohabiting)	1.75 (1.67–1.84)
							Income:	
							Intermediate vs low	2.08 (1.96–2.19)
							High vs low	2.24 (2.10–2.39)
							Intermediate vs high	1.08 (1.02–1.14)
Zidar	2015	Sweden	52541	2011	2012	cross-sectional	NA	NA

SES: socioeconomic status/deprivation

For peer review only

Supplementary file E: Studies included in review but not in meta-analyses or narrative synthesis

Author	Publication year	N in analysis	Study design	Analysis status	Explanation
Allgood	2016	22828	RCT	Intervention was confounder	Control-group-only data not calculable for other factors
Barlow	2019	305568	cohort	No useable data	Age categories could not be pooled
Bourmaud	2016	15844	RCT	Intervention was confounder	Control-group-only data not calculable for other factors
Douglas	2016	NA	cohort	No useable data	Percentages reported, but not Ns per category
Finney Rutten	2014	62754	cohort	No useable factors	Age categories could not be pooled
Gatrell	1998	24000	cohort	No useable factor	Reports uptake by health provider characteristic(s)*
Goldzahl	2018	26495	RCT	Intervention was confounder	Control-group-only data not calculable for other factors
Hyndman	2000	5316	cohort	No useable data	Could not isolate data for different factors
Luckman	2019	10063	RCT	Intervention was confounder	Control-group data for age was reported, but categories could not be pooled
Mayer	2000	1562	RCT	Intervention was confounder	Control-group-only data not calculable for other factors
Meldrum	1994	3083	RCT	Intervention was confounder	Control-group-only data not calculable for other factors
Merrick	2015	4427	RCT	Intervention was	Control-group-only data not

For peer review only - http://bmjopen.bmj.com/site/about/guidelines.xhtml

				confounder	calculable for other factors
Moss	2001	210939	Cohort	No useable data	Age categories could not be pooled
Offman	2013	12929	RCT	Intervention was confounder	Control-group data for age was reported, but categories could not be pooled
Pelfrene	1998	40713	cohort	No useable data	Percentages reported, but not Ns per category
Pinckney	2003	41844	cohort	No useable data	Age data could not be pooled; attendance data by other factors could not be calculated
Richards	2001	5732	RCT	Intervention was confounder	Control-group-only data not calculable for other factors
Rodriguez	1995	1859	cohort	No useable data	Inadequate data reported
Scaf-Klomp	1995	6898	cohort	No useable data	Risk of double-counting participants; time-series data
Segnan	1998	8069	RCT	Intervention was confounder	Control-group-only data not calculable for other factors
Simon	2001	1718	RCT	Intervention was confounder	Control-group-only data not calculable for other factors
Sutradhar	2016	2389889	cohort	No useable data	Does not report data adequate for calculating ORs
Taplin	1994	1322	RCT	Intervention was confounder	Control-group-only data not calculable for other factors
Vidal	2014	12475	quasi-experimental	Intervention was confounder	Control-group-only data not calculable for other factors
Visser	2005	825523	Cohort	No useable	Country of origin

For peer review only

				factors	data could not be pooled
Wilf-Miron	2011	157928	cohort	No useable data	Ns and % attendance not reported
Williams	1989	392	RCT	Intervention was confounder	Control-group-only data not calculable for other factors
Yarnall	1993	unclear	case-control	No useable factor	Reports uptake by health provider characteristic(s)*
Zidar	2015	52541	cross-sectional	No useable data	Reports % non-attendance by age group, but no N per category

*Attendance was measured based on a characteristic or behaviour of the medical provider or facility, not a characteristic of the patient, for example, physician's gender, health centre's use of a special assessment form, or the social deprivation status of the health centre (rather than the patient).

For peer review only

Supplementary file E: Funnel plots

Funnel Plots (note the central estimates come from fixed effects analyses, as these are the only way to assess bias, but means they will not match with the random effects estimates in the main paper)

Age

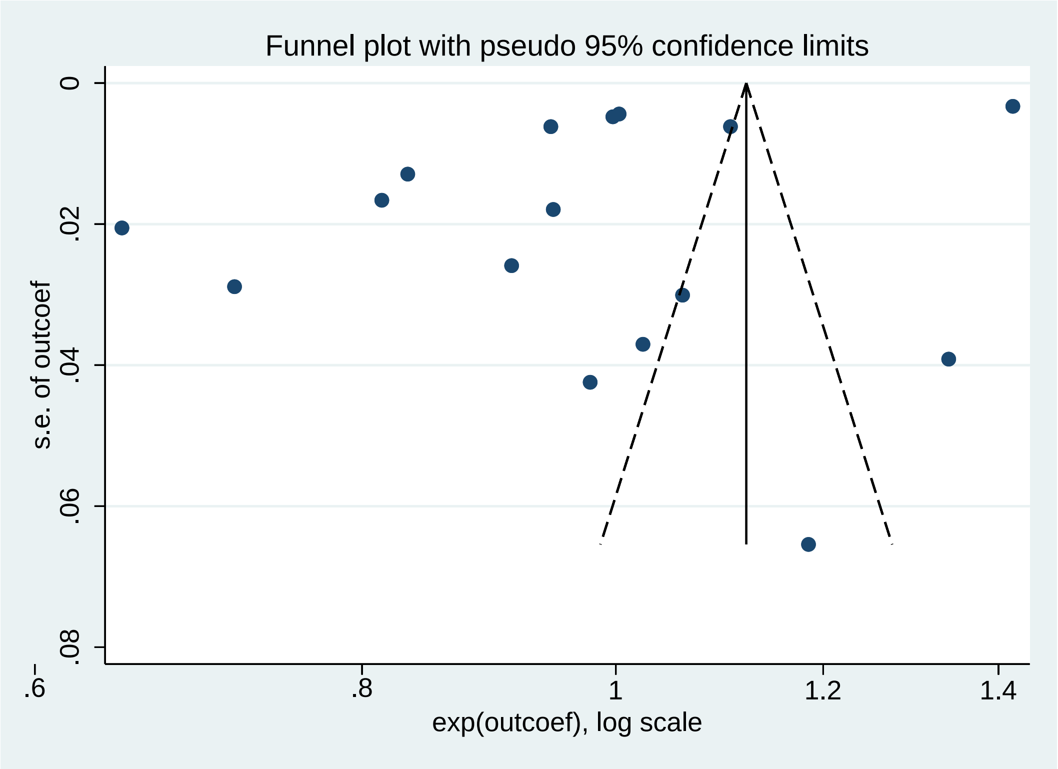

Homeowner

For peer review only

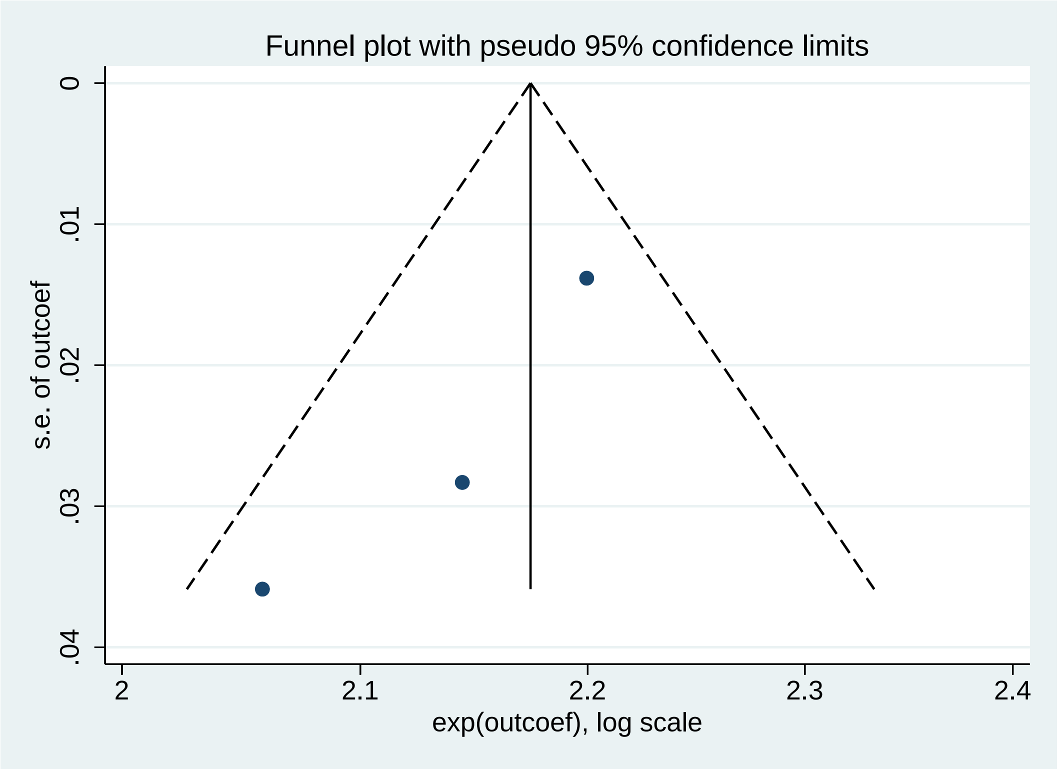

Marital status

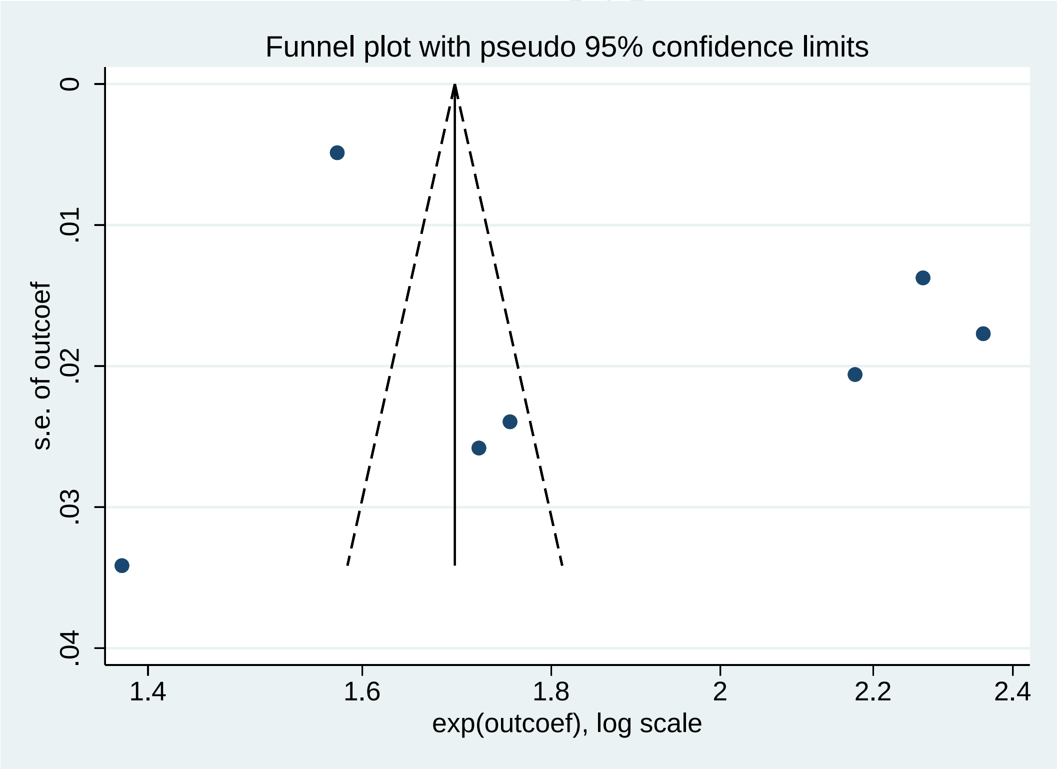

Immigrant status

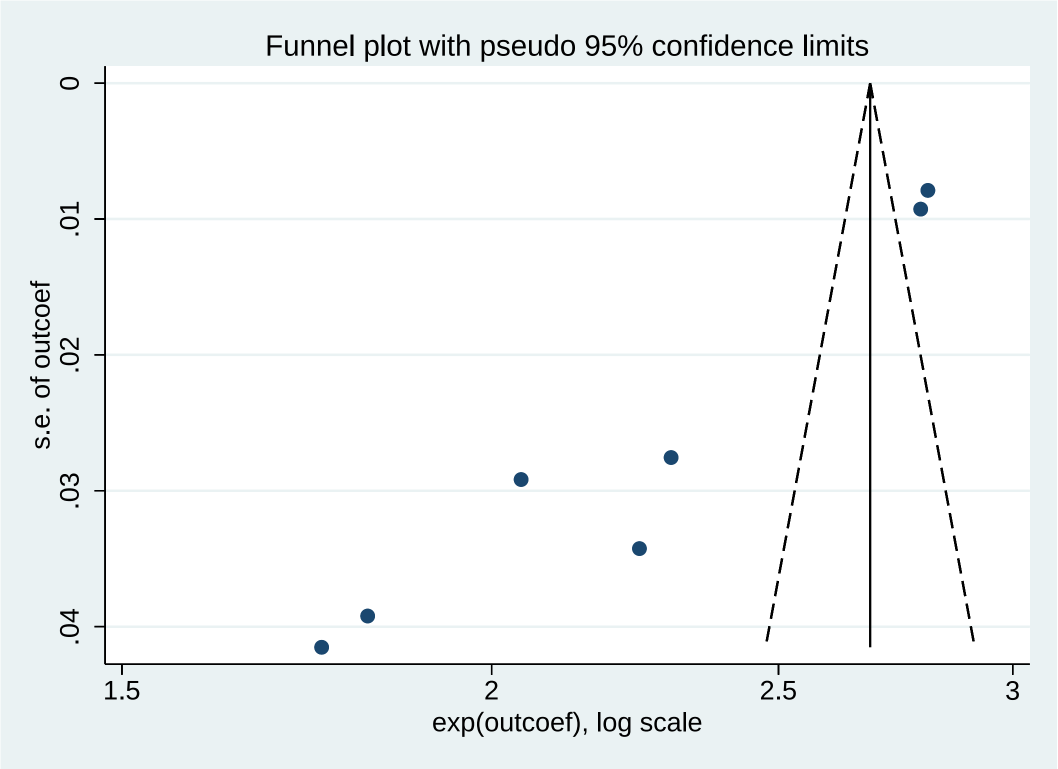

Reattendance

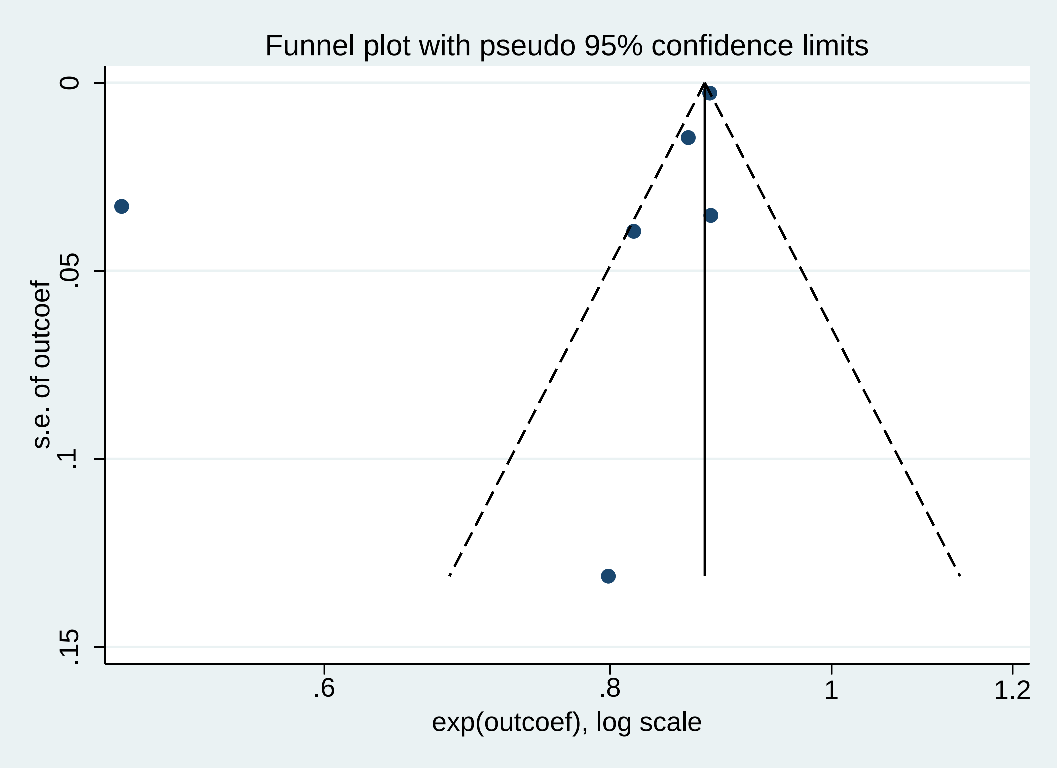

These graphs demonstrate the high level of heterogeneity present between the studies in these analyses.

Age is the only analysis where the studies disagree over the direction of attendance, however the disagreement is among larger studies, suggesting this is unlikely to be associated with biased reporting and instead down to the study heterogeneity. All other analyses, whilst having studies which disagree on the point estimate, have agreement as to which group is more or less likely to attend breast cancer screening.

Overall, we are not concerned about reporting bias.

For peer review only
